# Assessing the order of magnitude of outcomes in single-arm cohorts through systematic comparison with corresponding cohorts: An example from the AMOS study

**DOI:** 10.1186/1471-2288-8-11

**Published:** 2008-03-19

**Authors:** Harald J Hamre, Anja Glockmann, Wilfried Tröger, Gunver S Kienle, Helmut Kiene

**Affiliations:** 1 Institute for Applied Epistemology and Medical Methodology, Freiburg, Germany; 2 Director, Clinical Research Dr Tröger, Freiburg, Germany

## Abstract

**Background:**

When a therapy has been evaluated in the first clinical study, the outcome is often compared descriptively to outcomes in corresponding cohorts receiving other treatments. Such comparisons are often limited to selected studies, and often mix different outcomes and follow-up periods. Here we give an example of a systematic comparison to all cohorts with identical outcomes and follow-up periods.

**Methods:**

The therapy to be compared (anthroposophic medicine, a complementary therapy system) had been evaluated in one single-arm cohort study: the Anthroposophic Medicine Outcomes Study (AMOS). The five largest AMOS diagnosis groups (A-cohorts: asthma, depression, low back pain, migraine, neck pain) were compared to all retrievable corresponding cohorts (C-cohorts) receiving other therapies with identical outcomes (SF-36 scales or summary measures) and identical follow-up periods (3, 6 or 12 months). Between-group differences (pre-post difference in an A-cohort minus pre-post difference in the respective C-cohort) were divided with the standard deviation (SD) of the baseline score of the A-cohort.

**Results:**

A-cohorts (5 cohorts with 392 patients) were similar to C-cohorts (84 cohorts with 16,167 patients) regarding age, disease duration, baseline affection and follow-up rates. A-cohorts had ≥ 0.50 SD larger improvements than C-cohorts in 13.5% (70/517) of comparisons; improvements of the same order of magnitude (small or minimal differences: -0.49 to 0.49 SD) were found in 80.1% of comparisons; and C-cohorts had ≥ 0.50 SD larger improvements than A-cohorts in 6.4% of comparisons. Analyses stratified by diagnosis had similar results. Sensitivity analyses, restricting the comparisons to C-cohorts with similar study design (observational studies), setting (primary care) or interventions (drugs, physical therapies, mixed), or restricting comparisons to SF-36 scales with small baseline differences between A- and C-cohorts (-0.49 to 0.49 SD) also had similar results.

**Conclusion:**

In this descriptive analysis, anthroposophic therapy was associated with SF-36 improvements largely of the same order of magnitude as improvements following other treatments. Although these non-concurrent comparisons cannot assess comparative effectiveness, they suggest that improvements in health status following anthroposophic therapy can be clinically meaningful. The analysis also demonstrates the value of a systematic approach when comparing a therapy cohort to corresponding therapy cohorts.

## Background

In the early phase of the clinical evaluation of a therapy, when first study results are published, it can be desirable to assess outcomes of this reference therapy relative to outcomes of other treatments for the disease in question. At this stage, a systematic review of controlled studies of the reference therapy vs. other treatments will give limited information, because few such studies will be available. An alternative is to compare the reference cohort (or cohorts) to all corresponding cohorts, i. e. to single-arm cohorts and therapy arms in controlled studies, receiving other treatments. Although such 'all corresponding cohorts comparisons' cannot assess comparative effectiveness, they nevertheless yield information about the order of magnitude of treatment outcomes. For therapies which have been evaluated exclusively in single-arm studies (e. g. many drugs [1-4], surgery [5-8], other procedures [9,10]), 'corresponding cohort comparisons' remain the only possibility.

Brief comparisons with corresponding cohorts are often presented in discussion sections of papers (e. g. [11,12]), and are often unsystematic (limited to selected studies) and imprecise (mixing different outcomes and follow-up periods). Here we give an example of a systematic comparative review, restricted to cohorts with identical outcome measures and comparable follow-up periods. Since the cohorts compared are derived from different studies, the comparisons are necessarily explorative and the analyses descriptive: results are not pooled, but are ordered in increasing magnitude.

## Methods

### Reason for the review

The reference therapy (anthroposophic medicine, a physician-provided complementary therapy system including counselling, medication, art and movement exercises, and massage) had been evaluated in a large single-arm cohort study: the Anthroposophic Medicine Outcomes Study (AMOS) [13]. AMOS was conducted in 1998–2005 in Germany. Outpatients with chronic disorders were enrolled before starting anthroposophic therapy and followed up for four years [14-17].

For the five largest AMOS diagnosis groups in adult patients (asthma, depression [18], low back pain [19], migraine, and neck pain), AMOS is so far the only outpatient study of anthroposophic therapy for the respective diagnoses [20]. In all five groups, changes in health status had been evaluated with the SF-36 Health Survey, which is widely used [21], enabling comparisons to other cohorts. We conducted a systematic review, comparing these five cohorts (A-cohorts, 392 patients from 90 medical practices, enrolled up to 31 December 2005) to all retrievable patient cohorts (C-cohorts) with corresponding diagnoses, outcome measure (SF-36), and follow-up periods.

### Objective

The objective of this systematic review was to assess the comparative order of magnitude of pre-post changes in health status in adult patients receiving anthroposophic therapy for one of five chronic diseases.

### Eligible comparison studies

For comparison to A-cohorts, we considered prospective studies from any setting in any country with any therapeutic intervention including treatment-as-usual, and with a cohort of at least 20 evaluable patients, published in Danish, English, German, French, Italian, Norwegian, Russian, Spanish or Swedish.

Studies were eligible if at least 80% of participants of the study or of a defined subgroup had one of the following five diagnoses occurring in at least 20 adult AMOS patients: asthma, depression, low back pain, migraine, and neck pain. No requirements of diagnostic criteria were made. Low back pain cohorts with more than 25% patients with congenital spinal malformations, spinal infectious or malignant disease, ankylosing spondylitis, Behcet's Syndrome, Reiter's Syndrome, osteoporosis with vertebral fracture, spinal stenosis, spondylolysis, spondylolisthesis, fibromyalgia, traumatic vertebral fracture or previous spinal operations were excluded from the analysis (these diagnoses were excluded from the corresponding A-cohort). Studies with all persons aged ≥ 60 years were also excluded (only 12% of A-patients were aged ≥ 60 years).

Studies were required to have at least one of the following ten outcomes from the SF-36 Health Survey, four-week version: eight SF-36 scales (Physical Function, Role Physical, Role Emotional, Social Functioning, Mental Health, Bodily Pain, Vitality, General Health), SF-36 Physical Component Summary Measure or SF-36 Mental Component Summary Measure. The outcome was included if the arithmetic mean was presented with a number or could be estimated from a figure (a) before commencement of any study intervention, and (b) after three, six, or 12 months (± 20%).

### Literature search

We searched Ovid MEDLINE, Ovid MEDLINE In-Process & Other Non-Indexed Citations, Journals@Ovid full text, BIOSIS Previews, Cochrane Database of Systematic Review, American College of Physicians Journal Club, Database of Abstracts of Reviews of Effects, Cochrane Central Register of Controlled Trials, Psychlit, the online SF-36 database [22], literature references of retrieved articles and our own literature archive. Articles published up to December 2005 were considered.

The general search strategy was: "SF-36 keyword" in title, abstract or keyword AND "disease keyword" in title. SF-36 keywords were "SF-36" OR "Short-Form" OR "Medical Outcomes" OR "Quality of Life" OR "Disability" OR "Outcome Assessment (Health Care)". Disease keywords were "Asthma" OR "Depress*" OR "Dysthymic disorder" OR "Low back pain" OR "Spinal diseas*" OR "Migraine" OR "Neck Pain" OR ["Spinal Diseas*" AND "Neck"].

Articles were read and assessed for provisional inclusion by one reviewer. All provisionally included articles were re-assessed for fulfilment of eligibility criteria by a second reviewer. Disagreements about fulfilment of eligibility criteria were resolved by discussion. Data of included articles were extracted and entered into Microsoft Excel data files by one person; all entered data were subject to source document verification by another person.

### Analysis

Units of analysis were individual SF-36 outcomes of the smallest statistically independent cohorts. Data from cohorts which were not statistically independent (e. g. patients randomised to drug therapy with or without information booklet, outcomes not differing between the two treatment groups) were pooled prior to analysis.

For each C-cohort, the following study characteristics were extracted and tabulated: diagnosis, publication year, evaluable SF-36 outcomes, sample size at baseline, last evaluable follow-up, follow-up rates, design, setting, country, age, gender, disease duration, and study treatment.

The statistical analysis (SPSS 14.0) was descriptive. For each evaluable SF-36 outcome of a C-cohort, the pre-post difference (mean score at last evaluable follow-up minus mean baseline score) was subtracted from the corresponding difference of the respective A-cohort, yielding a mean outcome difference ((Mean_A-FU _- Mean_A-0_) - (Mean_C-FU _- Mean_C-0_)). For each of the ten SF-36 outcomes, mean outcome differences were analysed with summary statistics of distribution of the differences. In order to aggregate all differences of all outcomes, the differences were also expressed as between-group effect sizes through division by the standard deviation (SD) of the baseline score of the A-cohort ((Mean_A-FU _- Mean_A-0_) - (Mean_C-FU _- Mean_C-0_)/SD_A-0_). The baseline SD of the A-cohort was used instead of the SD of the C-cohort or a pooled SD from A- and C-cohorts because the SD was not available for many C-cohorts. To avoid redundancy when aggregating differences across SF-36 outcomes, comparisons of SF-36 Physical and Mental Component Summary Measures were not included if, for the C-cohort in question, all the eight SF-36 scales were evaluable for comparison. Effect sizes and baseline differences were classified as large (≥ 0.80), medium (0.50–0.79), small (0.20–0.49) and minimal (0.00–0.19) [23,24]. An improvement of the same order of magnitude was defined as a minimal-to-small effect size (range -0.49 to 0.49). Due to the descriptive nature of this analysis, no hypothesis testing was performed.

Analyses were performed for all comparisons and stratified by SF-36 outcomes, by diagnoses, and both. In addition, four sensitivity analyses (SA1-4) were performed in order to study effects of reducing the heterogeneity of the comparisons. In each SA, between-group effect sizes were reanalysed, restricting the number of comparisons according to study design, setting, intervention or baseline status: In SA1, study designs of C-cohorts were restricted to observational studies (non-randomised comparative studies and single-arm cohorts), i.e. excluding randomised trials, because the randomisation prerequisite might lead to a selection of patients with different characteristics, compared to observational studies such as AMOS. In SA2, settings of C-cohorts were restricted to primary care or health maintenance organizations, because most A-patients were recruited in primary care. In SA3, treatments of C-cohorts were restricted to drugs, physiotherapy or other physical therapies or mixed treatments, because these interventions were deemed to be most similar to the AMOS treatment modalities. In SA4, comparisons were restricted to SF-36 scales with small baseline differences (maximum 0.49 SD) between the respective A- and C-cohorts, because scales with large baseline differences may have differing room for improvement following therapy, for regression to the mean etc.

## Results

### Excluded publications

A total of 530 publications were excluded from this review for the following reasons: diagnosis not fulfilling eligibility criteria (n = 192 publications), no follow-up data (n = 129), no SF-36 data (n = 55), multiple publications (n = 43), SF-36 data presented without means (n = 31), cohort with all patients ≥ 60 years (n = 27), duration of follow-up differing > 20% from three, six, and 12 months, respectively (n = 20), no baseline SF-36 data (n = 12), cohort with < 20 patients (n = 8), SF-36 acute form only (n = 3), modified SF-36 (n = 3), language not fulfilling eligibility criteria (n = 2), no trial (n = 1), other (n = 4). A table of excluded publications with reasons for exclusion is provided in Additional file 1.

### Description of AMOS cohorts and corresponding cohorts

#### All diagnoses analysed together

The five A-cohorts with a total of 392 patients were compared to 84 C-cohorts with 16,167 patients. These 84 C-cohorts were presented in 63 publications (Table 1, for details see also Additional file 2). Diagnoses are described below and in Additional file 3. Seven of the 84 C-cohorts were published in the period 1994–1996, 15 in 1997–1999, 23 in 2000–2002 and 39 were published in 2003–2005. Evaluable outcomes of C-cohorts were: all eight SF-36 scales (n = 40 of 84 C-cohorts), SF-36 Physical or Mental Component Summary Measures or both (n = 11), all eight SF-36 scales plus SF-36 Physical or Mental Component Summary Measures or both (n = 20), less than all eight SF-36 scales (n = 13).

**Table 1:** Overview of cohorts, patients and diagnoses

**Diagnosis**	**AMOS cohorts**				**Corresponding cohorts**				
	
	**Cohorts**	**Patients**		**Patients per cohort**	**Publications**	**Cohorts**	**Patients**		**Patients per cohort**
	
	**N**	**N**	**Percent**	**Mean**	**N**	**N**	**N**	**Percent**	**Mean**
Asthma	1	56	14.3%	56	11	12	2,030	12.6%	169
Depression	1	174	44.4%	174	13	19	4,440	27.5%	234
Low back pain	1	80	20.4%	80	24	30	4,701	29.1%	157
Migraine	1	42	10.7%	42	13	21	4,240	26.2%	202
Neck pain	1	40	10.2%	40	2	2	756	4.7%	378
Total	5	392	100.0%	78	63	84	16,167	100.0%	192

Median sample size per cohort was 56 patients (interquartile range (IQR) 41–127 patients) for A-cohorts and 137 patients (IQR 65–244) for C-cohorts. The last evaluable follow-up ensued after three months in 23 of 84 C-cohorts, after six months in 32 C-cohorts and after 12 months in 29 C-cohorts. Three-month-follow-up rates were 87.5% and 83.0% in A- and C-cohorts, respectively; six-month rates were 82.1% and 79.1%; and 12-month rates were 78.8% and 72.2%.

Study designs of C-cohorts were randomised controlled trials (n = 40 of 84 C-cohorts), non-randomised comparative studies (n = 9) and single-arm cohort studies (n = 35). Study settings of A-patients were primary care practice (85.5%, 337 of 389 evaluable A-patients), referral practice (10.5%), and outpatient clinic (2.8%). Study settings of C-cohorts were primary care or health maintenance organization (n = 27 C-cohorts, 33.6% (5427/16,167) of C-patients), non-academic hospital or outpatient clinic (n = 19 C-cohorts, 25.2% of C-patients), academic hospital or outpatient clinic (n = 27, 22.0%) and other or not specified (n = 11, 18.1%).

The 84 C-cohorts came from the USA (n = 37), Germany (n = 13), United Kingdom (n = 11), Canada (n = 4), Australia (n = 3), Japan (n = 3), Italy (n = 2), Spain (n = 2), from eight other countries (each C-cohort: n = 1) and from more than one country (n = 1).

Mean age, weighted for sample size, was 44.4 years (SD 11.6) in A-cohorts and 44.4 years in C-cohorts (evaluable in 76 of 84 C-cohorts). The percentage of women was 83.2% (326/392) in A-cohorts and 59.6% (8,897/14,927) in C-cohorts (evaluable in 77 C-cohorts). Mean disease duration, weighted for sample size, was 10.5 years (SD 12.8) in A-cohorts and 12.8 years in C-cohorts (evaluable for 14 of 84 C-cohorts).

Main anthroposophic treatment modalities in A-patients were: eurythmy therapy (45.4%, 178 of 392 A-patients), art therapy (26.5%), rhythmical massage therapy (11.5%), and physician-provided anthroposophic therapy (16.6%). Study treatments in C-cohorts were: drugs (n = 32 of 84 C-cohorts, 47.3%, 7,655 of 16,167 C-patients), treatment-as-usual (n = 17 C-cohorts, 14.9% of C-patients), surgery (n = 8, 9.0%), physiotherapy (n = 4, 10.1%), other physical therapy (n = 5, 1.4%), educational intervention (n = 7, 5.9%), and mixed or other therapy (n = 11, 11.4%).

#### Analyses stratified by diagnoses

Data on gender, age, study design, setting, disease duration at baseline, study treatments, last follow-up and follow-up rates, stratified by diagnosis, are presented in Additional file 3. The diagnosis neck pain had only two C-cohorts; therefore, the following description refers to the remaining diagnoses – asthma, depression, low back pain and migraine – with a range of 12–30 C-cohorts per diagnosis. In all four diagnoses, the percentage of women was higher in A-cohorts than in C-cohorts; absolute percent differences ranged from 9% (asthma: 70% and 61% women in A- and C-cohorts, respectively) to 44% (low back pain: 86% and 42% women). Age was similar in A- and C-cohorts. In C-cohorts the proportion of randomised trials was higher in depression (84%, 16 of 19 C-cohorts) than in other diagnoses (range 19%–43%). In A-patients the proportion recruited in primary care was lower in asthma (48%, 27 of 56 patients) than in other diagnoses (88%–97%). Correspondingly, the proportion of C-cohorts recruited in primary care/health maintenance organization settings was lowest for asthma (8%, 1 of 12 C-cohorts) and highest in depression (74%, 14 of 19 C-cohorts). In asthma, disease duration was similar in A- and C-cohorts (median 14.5 years and 14.5 years, respectively; n = 6 evaluable C-cohorts), the other diagnoses had only 1–3 cohorts with evaluable data on disease duration. Most frequent study treatments in C-cohorts were drugs (migraine, asthma, depression: 71%, 58% and 42% of C-cohorts, respectively) and treatment-as-usual (low back pain, 30% of C-cohorts). Follow-up-rates of A- and C-cohorts differed little across diagnoses.

### Comparisons between AMOS cohorts and corresponding cohorts

**Figure 1:**
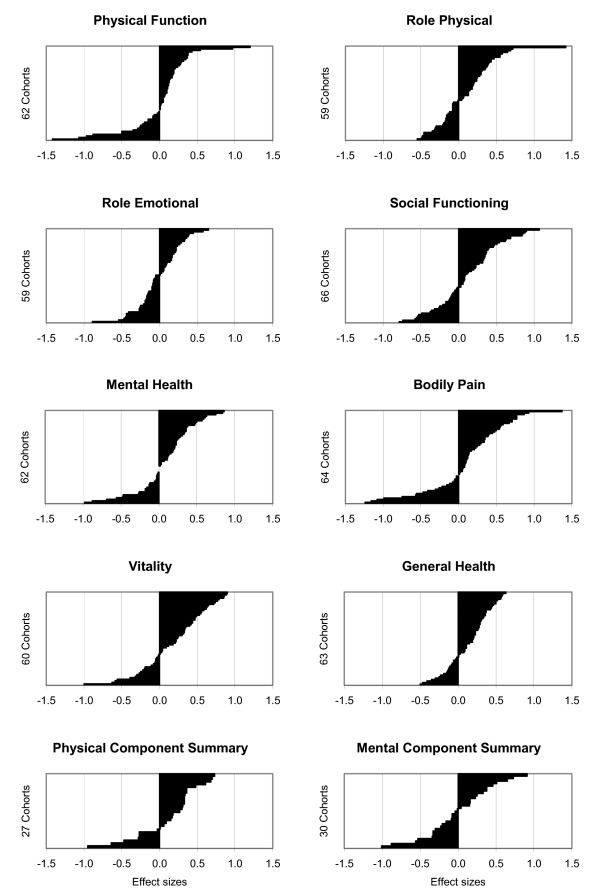
**Outcome comparisons stratified by individual SF-36 scales**. Differences between pre-post improvements of AMOS cohorts and improvements of corresponding cohorts for the eight SF-36 scales (0–100) and the SF-36 Physical and Mental Component Summary measures, expressed in effect sizes and ordered in increasing magnitude for each scale (altogether n = 552 comparisons). Positive differences indicate larger pre-post improvement in AMOS cohort than in corresponding cohort.

For separate analysis of individual SF-36 scales, a total of 552 comparisons between A-cohorts and C-cohorts were possible (Tables 2, 3, 4 Fig. 1). For aggregated analysis of all SF-36 scales, comparisons of SF-36 Physical and Mental Component Summary Measures were excluded for cohorts with all eight SF-36 scales evaluable (35 excluded comparisons), resulting in 517 comparisons.

**Table 2:** Baseline scores and outcome comparisons, stratified by SF-36 scales.

**SF-36 scale**	**Baseline**							**Follow-up**				
	
	**AMOS patients (N = 392 patients in 5 cohorts)**			**Corresponding cohorts (N = 552 cohorts)**				**Outcome differences (N = 552 comparisons)**				
	
	Mean *	SD *	Median *	**N**	Mean **	SD **	Median **	Mean ***	SD ***	25-per ***	Median ***	75-per ***
Physical Function	72.8	23.8	80.0	62	65.1	19.2	68.6	0.6	9.4	-2.0	2.0	4.4
Role Physical	37.4	38.5	25.0	59	40.0	18.3	43.4	4.1	14.1	-6.3	5.0	12.4
Role Emotional	42.4	41.5	33.3	59	59.2	16.6	62.2	-0.2	11.3	-7.5	-2.0	7.7
Social Functioning	54.8	25.1	50.0	66	61.0	12.9	60.7	2.6	9.2	-2.8	2.0	8.8
Mental Health	49.3	20.9	48.0	62	61.9	10.9	64.0	2.0	6.7	-0.9	2.2	6.4
Bodily Pain	48.8	28.1	41.0	64	45.8	15.5	44.8	2.7	10.0	-1.7	2.8	9.2
Vitality	34.3	18.0	35.0	60	45.6	9.6	47.4	3.4	6.9	-1.0	3.9	8.7
General Health	48.3	19.9	47.0	63	60.3	10.2	60.3	2.9	5.4	-1.3	4.0	6.9
Physical Component	41.7	10.2	41.3	27	37.3	7.6	37.7	1.5	3.9	-0.3	2.4	3.9
Mental Component	35.6	12.7	34.0	30	40.4	7.7	42.4	0.3	5.0	-3.6	-0.5	3.7

*of patients. **of cohorts. ***of differences. N: Number of cohorts. 25-per: 25-percentile. 75-per: 75-percentile. Each outcome difference refers to one SF-36 scale at the last evaluable follow-up of the corresponding cohort: Mean difference from baseline in AMOS cohort minus mean difference from baseline in corresponding cohort. A positive difference indicates that AMOS cohorts show larger improvements than corresponding cohorts.

**Table 3:** Baseline differences in standard deviations, stratified by SF-36 scales. Each comparison refers to one SF-36 scale at baseline: Mean score in AMOS cohort minus mean score in corresponding cohort, divided by standard deviation of score in AMOS cohort. A negative difference indicates that AMOS cohorts have worse health status than corresponding cohorts at baseline.

**SF-36 scale**	**Median**	**Interquartile range**	**Ranges: Number (percentage) of comparisons**							**N (total)**
				
			**≤ -0.8**	**-0.79 to -0.5**	**-0.49 to -0.2**	**-0.19 to 0.19**	**0.2 to 0.49**	**0.5 to 0.79**	**≥ 0.8**	
Physical Functioning	0.14	-0.20 to 0.72	0 (0)	0 (0)	16 (26)	19 (31)	9 (15)	7 (11)	11 (18)	62 (100)
Role Physical	-0.05	-0.40 to 0.30	3 (5)	6 (10)	15 (25)	15 (25)	18 (31)	2 (3)	0 (0)	59 (100)
Role Emotional	-0.14	-0.39 to 0.07	1 (2)	9 (15)	15 (25)	26 (44)	7 (12)	1 (2)	0 (0)	59 (100)
Social Functioning	-0.24	-0.56 to 0.18	7 (11)	14 (21)	15 (23)	14 (21)	10 (15)	5 (8)	1 (2)	66 (100)
Mental Health	-0.41	-0.59 to -0.19	7 (11)	17 (27)	22 (35)	13 (21)	2 (3)	1 (2)	0 (0)	62 (100)
Bodily Pain	-0.05	-0.41 to 0.13	7 (11)	7 (11)	10 (16)	29 (45)	6 (9)	4 (6)	1 (2)	64 (100)
Vitality	-0.41	-0.63 to -0.22	10 (17)	12 (20)	24 (40)	13 (22)	1 (2)	0 (0)	0 (0)	60 (100)
General Health	-0.54	-0.84 to -0.10	19 (30)	14 (22)	14 (22)	9 (14)	5 (8)	2 (3)	0 (0)	63 (100)
Physical Component Summary	0.40	-0.12 to 0.84	0 (0)	3 (11)	2 (7)	5 (19)	6 (22)	4 (15)	7 (26)	27 (100)
Mental Component Summary	-0.17	-0.55 to 0.06	2 (7)	8 (27)	4 (13)	13 (43)	3 (10)	0 (0)	0 (0)	30 (100)

**Table 4:** Outcome comparisons in effect sizes, stratified by SF-36 scales. Each comparison refers to one SF-36 scale at the last evaluable follow-up of the corresponding cohort: Mean difference from baseline in AMOS cohort minus mean difference from baseline in corresponding cohort, divided by standard deviation of baseline score of AMOS cohort. A positive difference indicates that AMOS cohorts show larger improvements than corresponding cohorts.

**SF-36 scale**	**Median**	**Interquartile range**	**Ranges: Number (percentage) of comparisons**							**N (total)**
				
			**≤ -0.8**	**-0.79 to -0.5**	**-0.49 to -0.2**	**-0.19 to 0.19**	**0.2 to 0.49**	**0.5 to 0.79**	**≥ 0.8**	
Physical Functioning	0.09	-0.09 to 0.20	4 (6)	2 (3)	6 (10)	35 (56)	12 (19)	1 (2)	2 (3)	62 (100)
Role Physical	0.13	-0.16 to 0.35	0 (0)	1 (2)	10 (17)	24 (41)	17 (29)	6 (10)	1 (2)	59 (100)
Role Emotional	-0.05	-0.18 to 0.21	1 (2)	1 (2)	10 (17)	32 (54)	13 (22)	2 (3)	0 (0)	59 (100)
Social Functioning	0.09	-0.12 to 0.37	0 (0)	6 (9)	7 (11)	24 (36)	18 (27)	6 (9)	5 (8)	66 (100)
Mental Health	0.12	-0.05 to 0.33	2 (3)	2 (3)	4 (6)	28 (45)	18 (29)	6 (10)	2 (3)	62 (100)
Bodily Pain	0.12	-0.06 to 0.44	4 (6)	3 (5)	5 (8)	25 (39)	13 (20)	11 (17)	3 (5)	64 (100)
Vitality	0.23	-0.06 to 0.52	1 (2)	3 (5)	7 (12)	16 (27)	18 (30)	11 (18)	4 (7)	60 (100)
General Health	0.20	-0.06 to 0.36	0 (0)	1 (2)	6 (10)	24 (38)	24 (38)	8 (13)	0 (0)	63 (100)
Physical Component Summary	0.27	-0.03 to 0.36	1 (4)	1 (4)	4 (15)	7 (26)	10 (37)	4 (15)	0 (0)	27 (100)
Mental Component Summary	-0.03	-0.31 to 0.34	2 (7)	2 (7)	6 (20)	10 (33)	6 (20)	3 (10)	1 (3)	30 (100)

#### Main analysis: all diagnoses and SF-36 scales analysed together (517 comparisons)

At baseline (Table 5), A-cohorts were slightly more severely affected than C-cohorts (median difference 0.22 SD, IQR -0.13 to +0.53); baseline differences between A- and C-cohorts were minimal or small (-0.49 SD to 0.49 SD) in 65.6% (339/517) of the comparisons; medium-to-large (≥ 0.50 SD) with A-cohorts more severely affected than C-cohorts in 26.5% of the comparisons; and medium-to-large with C-cohorts more severely affected in 7.9% of the comparisons.

**Table 5:** Baseline differences in standard deviations, stratified by diagnosis. Each comparison refers to one SF-36 scale at baseline: Mean score in AMOS cohort minus mean score in corresponding cohort, divided by standard deviation of score in AMOS cohort. A negative difference indicates that AMOS cohorts have worse health status than corresponding cohorts at baseline.

**Diagnosis**	**Median**	**Interquartile range**	**Ranges: Number (percentage) of comparisons**							**N (total)**
				
			**≤ -0.8**	**-0.79 to -0.5**	**-0.49 to -0.2**	**-0.19 to 0.19**	**0.2 to 0.49**	**0.5 to 0.79**	**≥ 0.8**	
Asthma	-0.02	-0.20 to +0.27	0 (0)	4 (5)	15 (20)	34 (44)	17 (22)	7 (9)	0 (0)	77 (100)
Depression	-0.25	-0.51 to +0.03	10 (13)	9 (12)	28 (36)	19 (24)	9 (12)	0 (0)	3 (4)	78 (100)
Low back pain	-0.05	-0.45 to +0.31	15 (7)	30 (15)	38 (19)	58 (29)	32 (16)	15 (7)	14 (7)	202 (100)
Migraine	-0.42	-0.64 to -0.17	28 (19)	36 (24)	48 (32)	31 (21)	6 (4)	1 (1)	0 (0	150 (100)
Neck pain	-0.37	-0.66 to -0.12	1 (10)	3 (30)	2 (20)	3 (30)	0 (0)	1 (10)	0 (0)	10 (100)
Total	-0.22	-0.53 to +0.13	55 (11)	82 (16)	131 (25)	144 (28)	64 (12)	24 (5)	17 (3)	517 (100)

**Figure 2:**
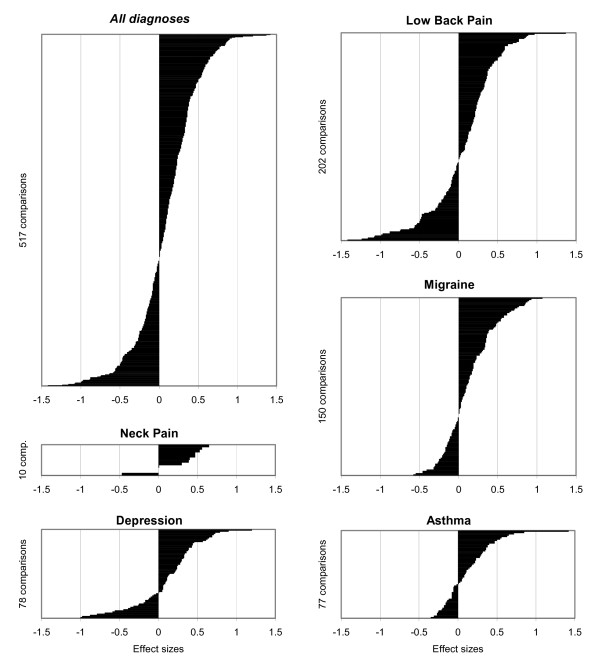
**Outcome comparisons stratified by diagnoses**. Differences between pre-post improvements of AMOS cohorts and improvements of corresponding cohorts for all SF-36 scales and summary measures, expressed in effect sizes and ordered in increasing magnitude: for all diagnoses and for individual diagnoses (altogether n = 517 comparisons). Positive effect sizes indicate larger pre-post improvement in AMOS cohort than in corresponding cohort.

At follow-up (Table 6, Fig. 2, All diagnoses), outcome comparisons showed effect sizes (pre-post improvements of A-cohorts minus pre-post improvements of C-cohorts divided by standard deviation of baseline score of A-cohorts) with a median of 0.11 (IQR -0.11 to 0.35).

**Table 6:** Outcome comparisons in effect sizes, stratified by diagnosis. Each comparison refers to one SF-36 scale at the last evaluable follow-up of the corresponding cohort: Mean difference from baseline in AMOS cohort minus mean difference from baseline in corresponding cohort, divided by standard deviation of baseline score of AMOS cohort. A positive difference indicates that AMOS cohorts show larger improvements than corresponding cohorts.

**Diagnosis**	**Median**	**Interquartile range**	**Ranges: Number (percentage) of comparisons**							**N (total)**
				
			**≤ -0.8**	**-0.79 to -0.5**	**-0.49 to -0.2**	**-0.19 to 0.19**	**0.2 to 0.49**	**0.5 to 0.79**	**≥ 0.8**	
Asthma	0.05	-0.18 to 0.30	1 (1)	4 (5)	13 (17)	33 (43)	18 (23)	6 (8)	2 (3)	77 (100)
Depression	0.17	-0.09 to 0.37	3 (4)	4 (5)	6 (8)	26 (33)	28 (36)	9 (12)	2 (3)	78 (100)
Low back pain	0.12	-0.12 to 0.33	9 (4)	10 (5)	22 (11)	77 (38)	57 (28)	21 (10)	6 (3)	202 (100)
Migraine	0.10	-0.08 to 0.35	0 (0)	2 (1)	16 (11)	77 (51)	34 (23)	14 (9)	7 (5)	150 (100)
Neck pain	0.44	0.23 to 0.54	0 (0)	0 (0)	1 (10)	1 (10)	5 (50)	3 (30)	0 (0)	10 (100)
Total	0.11	-0.11 to 0.35	13 (3)	20 (4)	58 (11)	214 (41)	142 (27)	53 (10)	17 (3)	517 (100)

• Effect sizes were positive, i. e. showing larger (≥ 0.20) improvements of A-cohorts than of C-cohorts in 41.0% (212/517) of the comparisons. These positive effect sizes were large (≥ 0.80) in 3.3% of the comparisons, medium (0.50–0.79) in 10.3% and small (0.20–0.49) in 27.5% of the comparisons.

• Effect sizes showed minimal differences (-0.19 to 0.19) between A- and C-cohorts in 41.4% of the comparisons.

• Effect sizes were negative, i. e. showing larger improvements of C-cohorts than of A-cohorts in 17.6% of the comparisons. These negative effect sizes were large (≥ 0.80) in 2.5% of the comparisons, medium (0.50–0.79) in 3.9% and small (0.20–0.49) in 11.2% of the comparisons.

The proportion of comparisons showing improvements in A- and C-cohorts of the same order of magnitude (minimal-to-small effect sizes, range -0.49 to 0.49) was 80.1% (414 of 517 comparisons).

#### Analyses stratified by SF-36 scales (552 comparisons)

Baseline scores of individual SF-36 scales in A- and C-cohorts are presented in Table 2, baseline between-group differences in standard deviations in Table 3. At baseline, A-cohorts were more severely affected than C-cohorts for 8 SF-36 scales, with median differences ranging from 0.05 SD (Role Physical, Bodily Pain) to 0.54 (General Health), while C-cohorts were more severely affected for 2 scales, with median differences of 0.14 (Physical Functioning) and 0.40 (Physical Component Summary). The proportion of baseline comparisons with minimal-to-small differences (-0.49 SD to 0.49 SD) ranged from 44% (General Health) to 81% (Role Physical, Role Emotional).

Outcome comparisons of individual SF-36 scales are presented in Table 2 (score differences) and in Table 4 and Fig. 1 (effect sizes). Median effect sizes ranged from -0.05 (Role Emotional) to 0.27 (Physical Component Summary), while the proportion of outcome comparisons with minimal-to-small differences (-0.49 to +0.49) ranged from 67% (Bodily Pain) to 93% (Role Emotional).

#### Analyses stratified by diagnosis (517 comparisons)

The diagnosis neck pain had only 10 comparisons; therefore the following description refers to the remaining diagnoses – asthma, depression, low back pain and migraine – with a range of 77–202 comparisons per diagnosis.

At baseline (Table 5), A-cohorts were more severely affected than C-cohorts in all four diagnoses: the median baseline difference in standard deviations (0.22 for all cohorts) ranged from 0.02 (asthma) to 0.42 (migraine), while the proportion of baseline comparisons with small baseline differences (-0.49 to +0.49 SD; 66% for all comparisons) ranged from 57% (migraine) to 86% (asthma).

Outcome comparisons in effect sizes (Table 6) showed very little variation: the median effect size (0.11 for all comparisons) ranged from 0.05 (asthma) to 0.17 (depression), while the proportion of comparisons with minimal-to-small differences (-0.49 to +0.49; 80% for all comparisons) ranged from 77% (depression and low back pain) to 85% (migraine).

#### Analyses stratified by SF-36 scales and diagnoses (552 comparisons)

These analyses are presented in Additional file 4.

#### Sensitivity analyses

Four sensitivity analyses were performed (Table 7, see Methods for details). SA1, SA2 and SA4 had very small effects on the outcome differences: in each analysis the median effect size was reduced from 0.11 to 0.08, while the proportion comparisons with minimal-to-small differences (-0.49 to +0.49 SD; 80% for all comparisons) ranged from 83% to 88%. In SA3, study settings of C-cohorts were restricted to primary care or health maintenance organizations, whereby the median effect size was increased from 0.11 to 0.24, while the proportion of comparisons with minimal-to-small differences was increased to 88%. The combination of SA1 + SA2 + SA3 + SA4 yielded only 5 evaluable cohorts with 16 comparisons, while results differed little from the main analysis (median effect size 0.07; minimal-to-small differences in 94% of comparisons).

**Table 7:** Outcome comparisons in effect sizes: Sensitivity analyses (SA)

**Analysis**	**Median**	**Interquartile range**	**Ranges: Number (percentage) of comparisons**							**N (total)**	**N**
			
			**≤ -0.8**	**-0.79 to -0.5**	**-0.49 to -0.2**	**-0.19 to 0.19**	**0.2 to 0.49**	**0.5 to 0.79**	**≥ 0.8**	**Comparisons**	**Cohorts**
Main analysis: any design, setting, intervention, and baseline status	0.11	-0.11 to 0.35	13 (3)	20 (4)	58 (11)	214 (41)	142 (27)	53 (10)	17 (3)	517 (100)	84
SA1: Design: only observational studies (excluding randomised controlled trials)	0.08	-0.14 to 0.28	6 (2)	11 (4)	40 (13)	140 (47)	77 (26)	21 (7)	6 (2)	301 (100)	44
SA2: Setting: only primary care/health maintenance organization	0.24	0.03 to 0.38	3 (2)	4 (3)	9 (7)	42 (30)	60 (43)	16 (12)	4 (3)	138 (100)	27
SA3: Intervention: only drugs, physiotherapy, other physical therapy, or mixed	0.08	-0.09 to 0.30	3 (1)	7 (2)	36 (11)	164 (50)	86 (26)	21 (6)	8 (2)	325 (100)	49
SA4: Baseline status: only comparisons with small baseline difference (< 0.50 standard deviation)	0.08	-0.13 to 0.28	5 (1)	14 (4)	41 (12)	161(47)	81 (24)	29 (9)	9 (3)	340 (100)	81
SA1 + SA2 + SA3 + SA4	0.07	-0.03 to 0.19	0 (0)	0 (0)	2 (13)	10 (63)	3 (19)	0 (0)	1 (6)	16 (100)	5

## Discussion

We have presented a systematic comparative review of SF-36 outcomes in five chronic conditions (asthma, depression, low back pain, migraine, neck pain). The review was prompted by the availability of results from the first study of a given therapy (the AMOS study of anthroposophic medicine in outpatients with various chronic diseases [13]). The objective was to assess the order of magnitude of AMOS outcomes, relative to outcomes of other therapies. For this purpose we compared AMOS diagnostic subgroups (A-cohorts) to all retrievable patient cohorts (C-cohorts) with corresponding diagnoses, outcome measures and follow-up periods. More than 500 comparisons of ten different SF-36 scales showed improvements largely of the same order of magnitude in corresponding A- and C-cohorts (minimal-to-small differences in 80% of the comparisons); with medium-to-large differences favouring A- and C-groups in 14% and 7% of the comparisons, respectively.

This systematic review has five characteristic features: 1) we compared one reference therapy to all other treatments for the respective indications; 2) each comparison was of corresponding cohorts from different studies; 3) comparisons were restricted to cohorts with identical outcome measure and comparable follow-up periods; 4) analyses were descriptive, with results ordered in increasing magnitude instead of being pooled; and 5) different outcomes were converted to a common metric, allowing for data synthesis into one variable. We are not aware of other systematic reviews combining these five features.

This type of review can be regarded as a systematic extension and upgrading of the common 'discussion-reviews' in publications presenting new therapies, where results of the first therapy study are compared descriptively to results of other treatments for the given indication. In contrast to such narrative reviews, the present review has the strengths of systematic, criteria-based literature selection and analysis.

For complementary and other complex therapy systems in widespread use regardless of whether evidence from randomised trials exists, it has been argued that the conventional drug research strategy – starting with studies of biological mechanisms and moving through Phase I, II and III clinical trials – should be replaced by a more appropriate strategy, moving from descriptive studies ('Phase 1') towards comparative studies of the whole system and its parts, and ending with studies of biological mechanisms ('Phase 5') [25]. In the context of this reversed strategy, the present review would represent an intermediate step between Phases 1–2 (studies of paradigms, utilization, perceived benefit and safety) and Phase 3 (comparative effectiveness studies).

Notably, the present review is limited to comparative order of magnitude. For our review, data from one single-arm study were available. Accordingly, each single comparison was of two cohorts derived from different studies. The only predefined criteria were comparable diagnosis and follow-up period, and identical outcome measure. A- and C-cohorts were found to be similar regarding age, disease duration, baseline affection and follow-up rates, and different regarding sample size and gender. Sensitivity analyses, restricting the number of comparisons to increase comparability of study design, settings, therapy, and baseline scores, had only small effects on the results. Notably, representative data on disease duration in C-cohorts was only available for one out of five diagnoses (asthma). Furthermore, only 15% of C-cohorts were from the same country as the A-cohorts (Germany). Other study characteristics of interest, including screening data, comorbidity and the use of adjunctive therapies, were only sparingly and heterogeneously documented in C-cohorts and hence were not evaluable. Because of the residual heterogeneity in these non-concurrent comparisons, the assessment could not be aimed at statistical precision. The analyses were purely descriptive, without attempting to pool data or to adjust for within-group differences (except simple adjustment for differences in baseline scores).

Our search strategy was limited to ten online databases, thus some eligible studies may have been missed. Sample sizes were less than 50 patients for A-cohorts with migraine and neck pain. Furthermore, only two cohorts with neck pain were available for comparison. Otherwise, a range of patient settings (primary care, clinic, academic hospital) and treatments (as-usual, drugs, physical therapies, educational intervention, surgery) were represented in the primary analyses of C-cohorts. Finally, the present review was restricted to a generic health status instrument (SF-36). Disease-specific comparisons might be more sensitive to relevant differences undetected by this review. On the other hand, anthroposophic therapy aims to improve a broad range of symptoms and functional limitations rather than only disease-specific symptoms [26], and broad instruments like the SF-36 may therefore be particularly appropriate [19].

Within the limits of non-concurrent comparisons, this review suggests that anthroposophic therapy for chronic asthma, back or neck pain, depression and migraine can be associated with improvements of SF-36 scales of largely the same order of magnitude as improvements following other treatments. This implication may sound trivial, but is not. If our analyses had shown mostly small improvements compared to other treatments, one might have concluded that further studies of SF-36 as outcome of anthroposophic therapy are not worthwhile. Had our analysis shown large differences favouring anthroposophic treatment, results would have appeared more impressive. Had our results been very heterogeneous (showing mostly large differences in both directions) it would have been necessary to compare C-cohorts with large positive and negative differences, respectively, to see if these two sets of cohorts differ systematically in other respects. The present results suggest that anthroposophic therapy can be associated with clinically meaningful improvements of health status.

The analysis also demonstrates the value of a systematic approach to corresponding cohort comparisons, which is particularly relevant for therapies evaluated exclusively or predominantly in single-arm studies. A relevant question in this respect is the appropriate range of C-cohorts to include and the associated workload (almost 600 publications were assessed for the present analysis). The starting point for the present comparative review was a reference cohort with many diagnoses; therefore we included the five largest evaluable diagnosis groups. In many other circumstances, it would be appropriate to analyse only one diagnosis. In the present review, we restricted comparisons to C-cohorts with similar follow-up period and identical outcome measure. Depending on the research question and the amount of available C-cohorts, further restrictions could be applied e. g. regarding design, setting, therapy and baseline status, as in the sensitivity analyses of the present review. Notably, in our analyses these restrictions, applied individually or simultaneously, had only small effects on the results: the maximum effect was an increase in the median effect size by 0.13 when the setting of C-cohorts was restricted to primary care/health maintenance organizations. Combined restrictions are possible, but will further reduce the number of C-cohorts. In this review, the combination of SA1 + SA2 + SA3 + SA4 resulted in only five evaluable C-cohorts with two diagnoses.

A problem when defining narrow inclusion criteria for any systematic review is that researchers familiar with the pool of potentially eligible studies might choose inclusion criteria that produce results favouring their research agenda (inclusion criteria bias) [27]. This problem can be prevented when broad criteria are used for the main analysis and restrictive criteria are applied secondarily, as in the present review. A general advantage of applying both broad and restricted inclusion criteria for one review is the additional information on how results change when inclusion criteria are altered.

Conversely, with some indications and outcome measures, the number of available C-cohorts may be very small. In such cases it may be necessary to widen the inclusion criteria, e. g. to include cohorts with other follow-up periods and outcome measures than in the reference study. Another scenario is the situation of having a body of C-cohorts from studies with very similar design, interventions and control groups (e. g. placebo-controlled randomised trials of one drug), which might enable researchers to incorporate data also from control groups, and to pool data and adjust for between-group differences [28]. Corresponding cohort comparisons may also be applied to safety aspects of medications and therapies, and should take into account setting (e. g. spontaneous reporting system, retrospective survey or prospective cohort) and outcome measure (e. g. adverse events, suspected adverse reactions or medically confirmed adverse reactions) [29].

## Conclusion

In this descriptive analysis, anthroposophic therapy was associated with SF-36 improvements largely of the same order of magnitude as improvements following other treatments. Although these non-concurrent comparisons cannot assess comparative effectiveness, they suggest that improvements in health status following anthroposophic therapy can be clinically meaningful. The analysis also demonstrates the value of a systematic approach when comparing a therapy cohort to corresponding therapy cohorts.

## Abbreviations

A-cohorts: AMOS cohorts; AMOS: Anthroposophic Medicine Outcomes Study; C-cohorts: corresponding cohorts; IQR: interquartile range, SD: standard deviation.

## Competing interests

The author(s) declare that they have no competing interests.

## Authors' contributions

HJH, GSK and HK designed the review. HJH and WT wrote the analysis plan. AG analysed data. HJH performed literature search, re-assessed provisionally included articles, was principal author of the paper, had full access to all data, and is guarantor. All authors contributed to manuscript drafting and revision and approved the final manuscript.

## Pre-publication history

The pre-publication history for this paper can be accessed here:



## Supplementary Material

Additional file 1 Excluded publications. List of excluded publications with reasons for exclusion Click here for file

Additional file 2 Included publications. List of included publications Click here for file

Additional file 3 Description of AMOS cohorts and corresponding cohorts, stratified by diagnosis. Descriptive data for AMOS cohorts and for corresponding cohorts on gender, age, study design, setting, disease duration at baseline, study treatment, last follow-up and follow-up rates Click here for file

Additional file 4 Comparative analyses, stratified by diagnosis and SF-36 scales. Baseline scores of AMOS cohorts and of corresponding cohorts as well as between-group outcome differences, stratified by diagnosis and SF-36 scales Click here for file
