# Supplementary material for: Assessing the order of magnitude of outcomes in single-arm cohorts through systematic comparison with corresponding cohorts: An example from the AMOS study
Source: BMC Med Res Methodol. 2008 Mar 19;8:11. doi: 10.1186/1471-2288-8-11 (PMC2323398; doi:10.1186/1471-2288-8-11)
Supplement: Additional file 1 — Excluded publications. List of excluded publications with reasons for exclusion [file 1471-2288-8-11-S1.pdf]

Excluded publications

Diagnosis	Publication	Reason for exclusion*
Asthma	Adams R, Ruffin R, Smith B. SF-36 is not well correlated with asthma quality of life questionnaire in hospital patients: Preliminary results from the Western Region Asthma Pilot Project (WRAPP). Eur Respir J - Suppl 1996; 9(23):374S.	No SF-36 data
Asthma	Adams R, Wakefield M, Wilson D, Parsons J, Campbell D, Smith B et al. Quality of life in asthma: a comparison of community and hospital asthma patients. J Asthma 2001; 38(3):205-214.	No follow-up data
Asthma	Alobid I, Benitez P, Bernal-Sprekelsen M, Guilemany JM, Picado C, Mullol J. The impact of asthma and aspirin sensitivity on quality of life of patients with nasal polyposis. Qual Life Res 2005; 14(3):789-793.	Diagnosis not fulfilling criteria
Asthma	Apter AJ, Reisine ST, Affleck G, Barrows E, ZuWallack RL. The influence of demographic and socioeconomic factors on health-related quality of life in asthma. J Allergy Clin Immunol 1999; 103(1 Pt 1):72-78.	No follow-up data
Asthma	Belenko LV, Leshchenko IV, Senkevich HI. [Effect of educational programs on the quality of life of patients with bronchial asthma]. [Russian]. Voen Med Zh 1996; 322(6):42-46.	No baseline SF-36 data
Asthma	Blumenschein K, Johannesson M. Relationship between quality of life instruments, health state utilities, and willingness to pay in patients with asthma. Ann Allergy Asthma Immunol 1998; 80(2):189-194.	SF-36 data without means
Asthma	Bonala SB, Pina D, Silverman BA, Amara S, Bassett CW, Schneider AT. Asthma severity, psychiatric morbidity, and quality of life: correlation with inhaled corticosteroid dose. J Asthma 2003; 40(6):691-699.	No follow-up data
Asthma	Bousquet J, Bullinger M, Fayol C, Marquis P, Valentin B, Burtin B. Assessment of quality of life in patients with perennial allergic rhinitis with the French version of the SF-36 Health Status Questionnaire. J Allergy Clin Immunol 1994; 94(2 Pt 1):182-188.	No follow-up data
Asthma	Caro JJ, Sr., Caro I, Caro J, Wouters F, Juniper EF. Does electronic implementation of questionnaires used in asthma alter responses compared to paper implementation? Qual Life Res 2001; 10(8):683-691.	No follow-up data
Asthma	Chan-Yeung M, Law B, Sheung SY, Lam CL. Internal consistency, reproducibility, responsiveness, and construct validity of the Chinese (HK) version of the asthma quality of life questionnaire. Qual Life Res 2001; 10(8):723-730.	No follow-up data
Asthma	Davis GE, Yueh B, Walker E, Katon W, Koepsell TD, Weymuller EA. Psychiatric distress amplifies symptoms after surgery for chronic rhinosinusitis. Otolaryngol Head Neck Surg 2005; 132(2):189-196.	Diagnosis not fulfilling criteria
Asthma	De Oliveira MA, Barbiere A, Santos LA, Faresin SM, Fernandes AL. Validation of a simplified quality-of-life questionnaire for socioeconomically deprived asthma patients. J Asthma 2005; 42(1):41-44.	Follow-up duration ± > 20%
Asthma	Dyer CA, Sinclair AJ. A hospital-based case-control study of quality of life in older asthmatics. Eur Respir J 1997; 10(2):337-341.	Patients > 60 years
Asthma	Eisner MD, Yelin EH, Henke J, Shiboski SC, Blanc PD. Environmental tobacco smoke and adult asthma. The impact of changing exposure status on health outcomes. Am J Respir Crit Care Med 1998; 158(1):170-175.	Other
Asthma	Erickson SR, Christian R Asthma D, Jr., Kirking DM, Halman LJ. Relationship between patient and disease characteristics, and health-related quality of life in adults with asthma. Respir Med 2002; 96(6):450-460.	No SF-36 data
Asthma	Erickson SR, Kirking DM. Comparison of the SF-12 and SF-36 summary scores in a managed care asthmatic population. Pharmacotherapy 1997;	No follow-up data

	17(5):1106-1107.	
Asthma	Espinosa De Los Monteros MJ, Alonso J, Ancochea J, Gonzalez A. [Quality of life in asthma: reliability and validity of the short form generic questionnaire (SF-36) applied to the population of asthmatics in a public health area]. [Spanish]. Arch Bronconeumol 2002; 38(1):4-9.	No follow-up data
Asthma	Fish JE, Karpel JP, Craig TJ, Bensch GW, Noonan M, Webb DR et al. Inhaled mometasone furoate reduces oral prednisone requirements while improving respiratory function and health-related quality of life in patients with severe persistent asthma. J Allergy Clin Immunol 2000; 106(5):852-860.	Multiple publication
Asthma	Hooi LN. What are the clinical factors that affect quality of life in adult asthmatics? Med J Malaysia 2003; 58(4):506-515.	No follow-up data
Asthma	Huang IC, Dominici F, Frangakis C, Diette GB, Damberg CL, Wu AW. Is risk-adjustor selection more important than statistical approach for provider profiling? Asthma as an example. Med Decis Making 2005; 25(1):20-34.	No follow-up data
Asthma	Huss K, Naumann PL, Mason PJ, Nanda JP, Huss RW, Smith CM et al. Asthma severity, atopic status, allergen exposure and quality of life in elderly persons. Ann Allergy Asthma Immunol 2001; 86(5):524-530.	Patients > 60 years
Asthma	Juniper Asthma EF, Guyatt GH, Cox FM, Ferrie PJ, King DR. Development and validation of the Mini Asthma Quality of Life Questionnaire. Eur Respir J 1999; 14(1):32-38.	Follow-up duration ± > 20%
Asthma	Juniper EF, Norman GR, Cox FM, Roberts JN. Comparison of the standard gamble, rating scale, AQLQ and SF-36 for measuring quality of life in asthma. Eur Respir J 2001; 18(1):38-44.	No follow-up data
Asthma	Katz PP, Alfieri WS. Satisfaction with abilities and well-being: development and validation of a questionnaire for use among persons with rheumatoid arthritis. Arthritis Care Res 1997; 10(2):89-98.	Other
Asthma	Keller SD, Bayliss MS, Ware JE, Hsu MA, Damiano AM, Goss TF. Comparison of responses to SF-36 Health Survey questions with one-week and four-week recall periods. Health Serv Res 1997; 32(3):367-384.	No follow-up data
Asthma	Knoell DL, Pierson JF, Marsh CB, Allen JN, Pathak DS. Measurement of outcomes in adults receiving pharmaceutical care in a comprehensive asthma outpatient clinic. Pharmacotherapy 1998; 18(6):1365-1374.	No SF-36 data
Asthma	Lee TA, Hollingworth W, Sullivan SD. Directly elicited preferences compared to preferences derived from the sf-36 in adult asthmatics. [Abstract]. Value Health 2002; 5(3):134-135.	No SF-36 data
Asthma	Leidy KN, Chan KS, Coughlin C. Is the asthma quality of life questionnaire a useful measure for low-income asthmatics? Am J Respir Crit Care Med 1998; 158(4):1082-1090.	No follow-up data
Asthma	Leynaert B, Neukirch C, Liard R, Bousquet J, Neukirch F. Quality of life in allergic rhinitis and asthma. A population-based study of young adults. Am J Respir Crit Care Med 2000; 162(4 Pt 1):1391-1396.	No follow-up data
Asthma	Mahajan P, Okamoto LJ, Schaberg A, Kellerman D, Schoenwetter WF. Impact of fluticasone propionate powder on health-related quality of life in patients with moderate asthma. J Asthma 1997; 34(3):227-234.	Multiple publication
Asthma	Mancuso CA, Rincon M, McCulloch CE, Charlson ME. Self-efficacy, depressive symptoms, and patients' expectations predict outcomes in asthma. Med Care 2001; 39(12):1326-1338.	No follow-up data
Asthma	Mancuso CA, Peterson MG. Different methods to assess quality of life from multiple follow-ups in a longitudinal asthma study. J Clin Epidemiol 2004; 57(1):45-54.	No baseline SF-36 data
Asthma	Matheson M, Raven J, Woods RK, Thien F, Walters EH, Abramson M. Wheeze not current asthma affects quality of life in young adults with asthma. Thorax 2002; 57(2):165-167.	No follow-up data

Asthma	Nishimura K, Hajiro T, Oga T, Tsukino M, Sato S, Ikeda A. A comparison of two simple measures to evaluate the health status of asthmatics: the Asthma Bother Profile and the Airways Questionnaire 20. J Asthma 2004; 41(2):141-146.	No SF-36 data
Asthma	Nishimura K, Hajiro T, Oga T, Tsukino M, Ikeda A. Health-related quality of life in stable asthma: what are remaining quality of life problems in patients with well-controlled asthma? J Asthma 2004; 41(1):57-65.	Follow-up duration ± > 20%
Asthma	Noonan M, Chervinsky P, Busse WW, Weisberg SC, Pinnas J, de Boisblanc BP et al. Fluticasone propionate reduces oral prednisone use while it improves asthma control and quality of life. Am J Respir Crit Care Med 1995; 152(5 Pt 1):1467-1473.	No baseline SF-36 data
Asthma	Osborne ML, Vollmer WM, Linton KL, Buist AS. Characteristics of patients with asthma within a large HMO: a comparison by age and gender. Am J Respir Crit Care Med 1998; 157(1):123-128.	No follow-up data
Asthma	Osman LM, Calder C, Robertson R, Friend JA, Legge JS, Douglas JG. Symptoms, quality of life, and health service contact among young adults with mild asthma Am J Respir Crit Care Med 2000; 161(2 Pt 1):498-503.	No follow-up data
Asthma	Ried LD, Nau DP, T.J. Evaluation of patient's Health-Related Quality of Life using a modified and shortened version of the Living With Asthma Questionnaire (ms-LWAQ) and the medical outcomes study, Short-Form 36 (SF-36). Qual Life Res 1999; 8(6):491-499.	No follow-up data
Asthma	Ringsberg KC, Timpka T. Clinical health education for patients with asthma-like symptoms but negative asthma tests. Allergy 2001; 56(11):1049-1054.	Diagnosis not fulfilling criteria
Asthma	Rydman RJ, Isola ML, Roberts RR, Zalenski RJ, McDermott MF, Murphy DG et al. Emergency Department Observation Unit versus hospital inpatient care for a chronic asthmatic population: a randomized trial of health status outcome and cost. Med Care 1998; 36(4):599-609.	Diagnosis not fulfilling criteria
Asthma	Sandez E, Vazquez MI, Romero-Frais E, Blanco-Aparicio M, Otero I, Verea H. Depression, Panic-Fear, and Quality of Life in Near-Fatal Asthma Patients. J Clin Psychol Med Settings 2005; 12(2):175-184.	No follow-up data
Asthma	Stavem K. Reliability and validity of the asthma quality of life questionnaire (AQLQ) and short form 36 (SF-36) in chronic obstructive pulmonary disease. Eur Respir J - Supplement 1996; 9(23):102S.	Diagnosis not fulfilling criteria
Asthma	Stavem K, Lossius MI, Kvien TK, Guldvog B. The health-related quality of life of patients with epilepsy compared with angina pectoris, rheumatoid arthritis, asthma and chronic obstructive pulmonary disease. Qual Life Res 2000; 9(7):865-871.	No follow-up data
Asthma	Steen N, Hutchinson A, McColl E, Eccles MP, Hewison J, Meadows KA et al. Development of a symptom based outcome measure for asthma. BMJ 1994; 309(6961):1065-1068.	SF-36 data without means
Asthma	Strine TW, Kobau R, Chapman DP, Thurman DJ, Price P, Balluz LS. Psychological distress, comorbidities, and health behaviors among U.S. adults with seizures: results from the 2002 National Health Interview Survey. Epilepsia 2005; 46(7):1133-1139.	Diagnosis not fulfilling criteria
Asthma	Szende A, Svensson K, Stahl E, Meszaros A, Berta GY. Psychometric and utility-based measures of health status of asthmatic patients with different disease control level. Pharmacoeconomics 2004; 22(8):537-547.	No follow-up data
Asthma	Terreehorst I, Duivenvoorden HJ, Tempels-Pavlica Z, Oosting AJ, de Monchy JG, Bruijnzeel-Koomen CA et al. The unfavorable effects of concomitant asthma and sleeplessness due to the atopic eczema/dermatitis syndrome (AEDS) on quality of life in subjects allergic to house-dust mites. Allergy 2002; 57(10):919-925.	Diagnosis not fulfilling criteria
Asthma	Terreehorst I, Duivenvoorden HJ, Tempels-Pavlica Z, Oosting AJ, de Monchy JG, Bruijnzeel-Koomen CA et al. The effect of encasings on quality of life in adult house dust mite allergic patients with rhinitis,	Diagnosis not fulfilling criteria

	asthma and/or atopic dermatitis. Allergy 2005; 60(7):888-893.	
Asthma	van der Molen T, Postma DS, Schreurs AJ, Bosveld HE, Sears MR, Meyboom-de Jong B. Discriminative aspects of two generic and two asthma-specific instruments: relation with symptoms, bronchodilator use and lung function in patients with mild asthma. Qual Life Res 1997; 6(4):353-361.	No follow-up data
Asthma	van der Molen T, Sears MR, de Graaff CS, Postma DS, Meyboom-de Jong B. Quality of life during formoterol treatment: comparison between asthma-specific and generic questionnaires. Canadian and the Dutch Formoterol Investigators. Eur Respir J 1998; 12(1):30-34.	Follow-up duration ± > 20%
Asthma	Viramontes JL, O'Brien B. Relationship between symptoms and health-related quality of life in chronic lung disease. J Gen Intern Med 1994; 9(1):46-48.	Diagnosis not fulfilling criteria
Asthma	Vollmer WM, O'Hollaren M, Ettinger KM, Stibolt T, Wilkins J, Buist AS et al. Specialty differences in the management of asthma. A cross-sectional assessment of allergists' patients and generalists' patients in a large HMO. Arch Intern Med 1997; 157(11):1201-1208.	No follow-up data
Asthma	Vondra V, Reisova M. [Quality of life in patients with chronic obstructive pulmonary disease and bronchial asthma]. [Czech]. Cas Lek Cesk 1998; 137(15):455-459.	Diagnosis not fulfilling criteria
Asthma	Ware JE, Kemp JP, Buchner DA, Singer AE, Nolop KB, Goss TF. The responsiveness of disease-specific and generic health measures to changes in the severity of asthma among adults. Qual Life Res 1998; 7(3):235-244.	Multiple publication
Asthma	Ware JE, Jr., Kemp JP, Buchner DA, Singer AE, Nolop KB, Goss TF. The responsiveness of disease-specific and generic health measures to changes in the severity of asthma among adults. Qual Life Res 1998; 7(3):235-244.	SF-36 acute form only
Asthma	Wyrwich KW, Nelson HS, Tierney WM, Babu AN, Kroenke K, Wolinsky FD. Clinically important differences in health-related quality of life for patients with asthma: an expert consensus panel report. Allergy Asthma Immunol 2003; 91(2):148-153.	No SF-36 data
Asthma	Yamaoka K, Iguchi E, Kubota S, Tashimo H, Kuramochi M, Wakabayashi H et al. [Usefulness of patient education and quality of life (QOL) evaluation in asthma patients]. [Japanese]. Arerugi - Japanese Journal of Allergology 2002; 51(12):1170-1176.	Language not fulfilling criteria
Asthma	Zillich AJ, Blumenschein K, Johannesson M, Freeman P. Assessment of the relationship between measures of disease severity, quality of life, and willingness to pay in asthma. Pharmacoeconomics 2002; 20(4):257-265.	No follow-up data
Depression	A nurse-led intervention reduced risk factors, anxiety, and depression in patients waiting for CABG. ACP Journal Club March/April, 2002;(2).	Diagnosis not fulfilling criteria
Depression	Achat H, Kawachi I, Spiro A, III, DeMolles DA, Sparrow D. Optimism and depression as predictors of physical and mental health functioning: the Normative Aging Study. Ann Behav Med 2000; 22(2):127-130.	Diagnosis not fulfilling criteria
Depression	Akashiba T, Kawahara S, Akahoshi T, Omori C, Saito O, Majima T et al. Relationship between quality of life and mood or depression in patients with severe obstructive sleep apnea syndrome. Chest 2002; 122(3):861-865.	Diagnosis not fulfilling criteria
Depression	Antunes HK, Stella SG, Santos RF, Bueno OF, de Mello MT. Depression, anxiety and quality of life scores in seniors after an endurance exercise program. Rev Bras Psiquiatr 2005; 27(4):266-271.	Patients > 60 years
Depression	Arroyo C, Hu FB, Ryan LM, Kawachi I, Colditz GA, Speizer FE et al. Depressive symptoms and risk of type 2 diabetes in women. Diabetes Care 2004; 27(1):129-133.	Diagnosis not fulfilling criteria
Depression	Ayuso-Mateos JL, Lasa L, Vazquez-Barquero JL, Oviedo A, Diez-	Diagnosis not

	Manrique JF. Measuring health status in psychiatric community surveys: internal and external validity of the Spanish version of the SF-36. Acta Psychiatr Scand 1999; 99(1):26-32.	fulfilling criteria
Depression	Barbui C, Garattini L, Krulichova I, Apolone G, Negri E, Ricci E et al. Health status, resource consumption, and costs of dysthymic patients in Italian primary care. Epidemiol Psichiatr Soc 2004; 13(2):120-125.	No follow-up data
Depression	Barrett JE, Williams JW, Jr., Oxman TE, Frank E, Katon W, Sullivan M et al. Treatment of dysthymia and minor depression in primary care: a randomized trial in patients aged 18 to 59 years. J Fam Pract 2001; 50(5):405-412.	No follow-up data
Depression	Bech P. Social functioning: should it become an endpoint in trials of antidepressants? CNS Drugs 2005; 19(4):313-324.	No Trial
Depression	Beghi E, Roncolato M, Visona G. Depression and altered quality of life in women with epilepsy of childbearing age. Epilepsia 2004; 45(1):64-70.	Diagnosis not fulfilling criteria
Depression	Benrud-Larson LM, Sandroni P, Schrag A, Low PA. Depressive symptoms and life satisfaction in patients with multiple system atrophy. Mov Disord 2005; 20(8):951-957.	Diagnosis not fulfilling criteria
Depression	Bettencourt N, Dias C, Mateus P, Sampaio F, Santos L, Adao L et al. Impact of cardiac rehabilitation on quality of life and depression after acute coronary syndrome. Rev Port Cardiol 2005; 24(5):687-696.	Diagnosis not fulfilling criteria
Depression	Beusterien KM, Nissenson AR, Port FK, Kelly M, Steinwald B, Ware JE, Jr. The effects of recombinant human erythropoietin on functional health and well-being in chronic dialysis patients. Clin J Am Soc Nephrol 1996; 7(5):763-773.	Patients > 60 years
Depression	Beusterien KM, Steinwald B, Ware JE, Jr. Usefulness of the SF-36 Health Survey in measuring health outcomes in the depressed elderly. J Geriatr Psychiatry Neurol 1996; 9(1):13-21.	Diagnosis not fulfilling criteria
Depression	Birks Y, Roebuck A, Thompson DR. A validation study of the Cardiac Depression Scale (CDS) in a UK population. Br J Health Psychol 2004; 9(Pt 1):15-24.	Diagnosis not fulfilling criteria
Depression	Bobes J, Gonzalez MP, Bascaran MT, Arango C, Saiz PA, Bousono M. Quality of life and disability in patients with obsessive-compulsive disorder. Eur Psychiatry 2001; 16(4):239-245.	Diagnosis not fulfilling criteria
Depression	Brown C, Schulberg HC, Shear MK. Phenomenology and severity of major depression and comorbid lifetime anxiety disorders in primary medical care practice. Anxiety 1996; 2(5):210-218.	Multiple publication
Depression	Brown C, Schulberg HC, Madonia MJ, Shear MK, Houck PR. Treatment outcomes for primary care patients with major depression and lifetime anxiety disorders. Am J Psychiatry 1996; 153(10):1293-1300.	Multiple publication
Depression	Buist-Bouwman MA, Ormel J, de Graaf R, Vollebergh WA. Functioning after a major depressive episode: complete or incomplete recovery? J Affect Disord 2004; 82(3):363-371.	Diagnosis not fulfilling criteria
Depression	Burton HJ, Kline SA, Cooper BS, Rabinowitz A, Dodek A. Assessing risk for major depression on patients selected for percutaneous transluminal coronary angioplasty: is it a worthwhile venture? Gen Hosp Psychiatry 2003; 25(3):200-208.	Diagnosis not fulfilling criteria
Depression	Buskila D. Fibromyalgia, chronic fatigue syndrome, and myofascial pain syndrome. Curr Opin Rheumatol March 2001;13(2):117-127 2001;(2):117-127.	Diagnosis not fulfilling criteria
Depression	Caap-Ahlgren M, Dehlin OA. Insomnia and depressive symptoms in patients with Parkinson's disease: Relationship to health-related quality of life. An interview study of patients living at home. Arch Gerontol Geriatr 2001; 32(1):23-33.	Diagnosis not fulfilling criteria
Depression	Callahan EJ, Bertakis KD, Azari R, Helms LJ, Robbins J, Miller J. Depression in primary care: patient factors that influence recognition.	SF-36 data without means

	Fam Med 1997; 29(3):172-176.	
Depression	Carson AJ, Ringbauer B, Stone J, McKenzie L, Warlow C, Sharpe M. Do medically unexplained symptoms matter? A prospective cohort study of 300 new referrals to neurology outpatient clinics. J Neurol Neurosurg Psychiatry 2000; 68(2):207-210.	Diagnosis not fulfilling criteria
Depression	Carson AJ, Postma K, Stone J, Warlow C, Sharpe M. The outcome of depressive disorders in neurology patients: a prospective cohort study. J Neurol Neurosurg Psychiatry 2003; 74(7):893-896.	Diagnosis not fulfilling criteria
Depression	Cass AR, Volk RJ, Nease DE. Health-related quality of life in primary care patients with recognized and unrecognized mood and anxiety disorders. Int J Psychiatry Med 1999; 29(3):293-309.	No follow-up data
Depression	Chengappa KR, Hennen J, Baldessarini RJ, Kupfer DJ, Yatham LN, Gershon S et al. Recovery and functional outcomes following olanzapine treatment for bipolar I mania. Bipolar Disord 2005; 7(1):68-76.	Diagnosis not fulfilling criteria
Depression	Chilvers C, Dewey M, Fielding K, Gretton V, Miller P, Palmer B et al. Antidepressant drugs and generic counselling for treatment of major depression in primary care: randomised trial with patient preference arms. BMJ 2001; 322(7289):772-775.	No SF-36 data
Depression	Choul FH, Chou P, Lin C, Su TT, Ou-Yang WC, Chien IC et al. The relationship between quality of life and psychiatric impairment for a Taiwanese community post-earthquake. Qual Life Res 2004; 13(6):1089-1097.	Diagnosis not fulfilling criteria
Depression	Chwastiak LA, von Korff M. Disability in depression and back pain: evaluation of the World Health Organization Disability Assessment Schedule (WHO DAS II) in a primary care setting. J Clin Epidemiol 2003; 56(6):507-514.	Diagnosis not fulfilling criteria
Depression	Coulehan JL, Schulberg HC, Block MR, Madonia MJ, Rodriguez E. Treating depressed primary care patients improves their physical, mental, and social functioning. Arch Intern Med 1997; 157(10):1113-1120.	No follow-up data
Depression	Cuijpers P, van Lammeren P. Secondary prevention of depressive symptoms in elderly inhabitants of residential homes. Int J Geriatr Psychiatry 2001; 16(7):702-708.	Patients > 60 years
Depression	Danieli E, Airo P, Bettoni L, Cinquini M, Antonioli CM, Cavazzana I et al. Health-related quality of life measured by the Short Form 36 (SF-36) in systemic sclerosis: correlations with indexes of disease activity and severity, disability, and depressive symptoms. Clin Rheumatol 2005; 24(1):48-54.	Diagnosis not fulfilling criteria
Depression	DeBattista C, Doghramji K, Menza MA, Rosenthal MH, Fieve RR, Modafinil in Depression Study Group. Adjunct modafinil for the short-term treatment of fatigue and sleepiness in patients with major depressive disorder: a preliminary double-blind, placebo-controlled study. J Clin Psychiatry 2003; 64(9):1057-1064.	No SF-36 data
Depression	Demin AA, Aksenova EA. [Efficacy of isosorbide-5-mononitrate retard in patients with stable exertional angina]. [Russian]. Klin Med (Mosk) 2005; 83(9):53-56.	Diagnosis not fulfilling criteria
Depression	Dennis CL. Influence of depressive symptomatology on maternal health service utilization and general health. Arch Womens Ment Health 2004; 7(3):183-191.	Diagnosis not fulfilling criteria
Depression	Dickens K. Randomised controlled trial and economic evaluation of screening for post-natal depression - RESPoND. National Research Register 2002.	Diagnosis not fulfilling criteria
Depression	Dogan E, Erkoc R, Eryonucu B, Sayarlioglu H, Agargun MY. Relation between depression, some laboratory parameters, and quality of life in hemodialysis patients. Ren Fail 2005; 27(6):695-699.	Diagnosis not fulfilling criteria
Depression	Doraiswamy PM, Khan ZM, Donahue RM, Richard NE. The spectrum of quality-of-life impairments in recurrent geriatric depression. J Gerontol A	Patients > 60 years

	Biol Sci Med Sci 2002; 57(2):M134-M137.	
Depression	Dowrick C, Casey P, Dalgard O, Hosman C, Lehtinen V, Vazquez-Barquero JL et al. Outcomes of Depression International Network (ODIN). Background, methods and field trials. ODIN Group. Br J Psychiatry 1998; 172:359-363.	SF-36 data without means
Depression	Dowrick C, Dunn G, Ayuso-Mateos JL, Dalgard OS, Page H, Lehtinen V et al. Problem solving treatment and group psychoeducation for depression: multicentre randomised controlled trial. BMJ 2000; 321(7274):1450-1454.	SF-36 data without means
Depression	Duggan C, Chilvers C, Dewey M, Fielding K, Gretton V. How disabling is depression? Evidence from a primary care sample. The Counselling Versus Antidepressants In Primary Care Study Group. Br J Gen Pract 1999; 49(439):95-98.	No follow-up data
Depression	Dyer CA, Hill SL, Stockley RA, Sinclair AJ. Quality of life in elderly subjects with a diagnostic label of asthma from general practice registers. Eur Respir J 1999; 14(1):39-45.	Diagnosis not fulfilling criteria
Depression	Elliott TE, Renier CM, Palcher JA. Chronic pain, depression, and quality of life: correlations and predictive value of the SF-36. Pain Med 2003; 4(4):331-339.	No follow-up data
Depression	Escalante A, del R, I, Mulrow CD. Symptoms of depression and psychological distress among Hispanics with rheumatoid arthritis. Arthritis Care Res 2000; 13(3):156-167.	Diagnosis not fulfilling criteria
Depression	Ettinger A, Reed M, Cramer J, Epilepsy Impact Project Group. Depression and comorbidity in community-based patients with epilepsy or asthma. Neurology 2004; 63(6):1008-1014.	Diagnosis not fulfilling criteria
Depression	Fifer SK, Buesching DP, Henke CJ, Potter LP, Mathias SD, Schonfeld WH et al. Functional status and somatization as predictors of medical offset in anxious and depressed patients. Value Health 2003; 6(1):40-50.	Diagnosis not fulfilling criteria
Depression	Fisher LJ, Goldney RD, Furze PF, Williams JL, Mattner J, McCleave DJ. Electroconvulsive therapy, depression, and cognitive outcomes: an Australian audit. J ECT 2004; 20(3):174-178.	No follow-up data
Depression	Fontana RJ, Moyer CA, Sonnad S, Lok ASF, Sneed-Pee N, Walsh J et al. Comorbidities and quality of life in patients with interferon-refractory chronic hepatitis C. Am J Gastroenterol 2001; 96(1):170-178.	Diagnosis not fulfilling criteria
Depression	Fossa SD, Dahl AA. Short Form 36 and Hospital Anxiety and Depression Scale. A comparison based on patients with testicular cancer. J Psychosom Res 2002; 52(2):79-87.	Diagnosis not fulfilling criteria
Depression	Franco GP, Barros AL, Nogueira-Martins LA. [Quality of life and depressive symptoms in nursing residents]. [Portuguese]. Rev Lat Am Enfermagem 2005; 13(2):139-144.	No follow-up data
Depression	Frank LB, Matza LS, Revicki DA, Chung JY. Depression and health-related quality of life for low-income African-American women in the U.S. Qual Life Res 2005; 14(10):2293-2301.	No follow-up data
Depression	Frayne SM, Seaver MR, Loveland S, Christiansen CL, Spiro A, III, Parker VA et al. Burden of medical illness in women with depression and posttraumatic stress disorder. Arch Intern Med 2004; 164(12):1306-1312.	No follow-up data
Depression	Friedman B, Heisel M, Delavan R. Validity of the SF-36 five-item Mental Health Index for major depression in functionally impaired, community-dwelling elderly patients. J Am Geriatr Soc 2005; 53(11):1978-1985.	Patients > 60 years
Depression	Fukuhara S, Ware JE, Kosinski M, Wada S, Gandek B. Psychometric and clinical tests of validity of the Japanese SF-36 Health Survey. J Clin Epidemiol 1998; 51(11):1045-1053.	No follow-up data
Depression	Fumaz CR, Barcelo M, Romeu E, Tuldra A, Ferrer M, Ballesteros A et al. Depression and quality of life in HIV-1+ patients with chronic hepatitis C virus infection treated with interferon and ribavirin. Abstracts of the Interscience Conference on Antimicrobial Agents & Chemotherapy 2002;	Diagnosis not fulfilling criteria

	42 2002~ 277:2777.	
Depression	Fusar-Poli PARA, E-mail:, Martinelli V, Klersy C, Campana C, Callegari A et al. Depression and quality of life in patients living 10 to 18 years beyond heart transplantation. J Heart Lung Transplant 2005; 24(12):2269-2278.	Diagnosis not fulfilling criteria
Depression	Gallegos-Orozco JF, Fuentes AP, Gerardo AJ, Perez-Pruna C, Hinojosa-Becerril C, Sixtos-Alonso MS et al. Health-related quality of life and depression in patients with chronic hepatitis C. Arch Med Res 2003; 34(2):124-129.	Diagnosis not fulfilling criteria
Depression	Gensichen J, Torge M, Peitz M, Wendt-Hermainski H, Beyer M, Rosemann T et al. Case management for the treatment of patients with major depression in general practices--rationale, design and conduct of a cluster randomized controlled trial--PRoMPT (Primary care Monitoring for depressive Patient's Trial) [ISRCTN66386086]- study protocol. BMC Public Health 2005; 5:101, 2005.	Multiple publication
Depression	Gilbody SM, House AO, Sheldon TA. Routinely administered questionnaires for depression and anxiety: systematic review. BMJ 2001; 322(7283):406-409.	No SF-36 data
Depression	Gleason OC, Yates WR, Isbell MD, Philipsen MA. An open-label trial of citalopram for major depression in patients with hepatitis C. J Clin Psychiatry 2002; 63(3):194-198.	Diagnosis not fulfilling criteria
Depression	Gleason OC, Yates WR, Philipsen MA. Major depressive disorder in hepatitis C: an open-label trial of escitalopram. Prim Care Companion J Clin Psychiatry 2005; 7(5):225-230.	Diagnosis not fulfilling criteria
Depression	Goldney RD, Ruffin R, Fisher LJ, Wilson DH. Asthma symptoms associated with depression and lower quality of life: a population survey. Med J Aust 2003; 178(9):437-441.	Diagnosis not fulfilling criteria
Depression	Goldney RD, Phillips PJ, Fisher LJ, Wilson DH. Diabetes, depression, and quality of life: a population study. Diabetes Care 2004; 27(5):1066-1070.	No follow-up data
Depression	Goldney RD, Fisher LJ, Dal Grande E, Taylor AW. Subsyndromal depression: prevalence, use of health services and quality of life in an Australian population. Soc Psychiatry Psychiatr Epidemiol 2004; 39(4):293-298.	No follow-up data
Depression	Goldney RD, Fisher LJ. Double depression in an Australian population. Soc Psychiatry Psychiatr Epidemiol 2004; 39(11):921-926.	No follow-up data
Depression	Goodacre S, Nicholl J. A randomised controlled trial to measure the effect of chest pain unit care upon anxiety, depression, and health-related quality of life [ISRCTN85078221]. Health Qual Life Outcomes 2004; 2(1):39.	Diagnosis not fulfilling criteria
Depression	Grov EK, Dahl AA, Moum T, Fossa SD. Anxiety, depression, and quality of life in caregivers of patients with cancer in late palliative phase. Ann Oncol 2005; 16(7):1185-1191.	Diagnosis not fulfilling criteria
Depression	Gual A, Balcells M, Torres M, Madrigal M, Diez T, Serrano L. Sertraline for the prevention of relapse in detoxicated alcohol dependent patients with a comorbid depressive disorder: a randomized controlled trial. Alcohol Alcohol 2003; 38(6):619-625.	Diagnosis not fulfilling criteria
Depression	Gulseren L, Gulseren S, Hekimsoy Z, Mete L. Comparison of fluoxetine and paroxetine in type II diabetes mellitus patients. Arch Med Res 2005; 36(2):159-165.	Diagnosis not fulfilling criteria
Depression	Hart S, Fonareva I, Merluzzi N, Mohr DC. Treatment for depression and its relationship to improvement in quality of life and psychological well-being in multiple sclerosis patients. Qual Life Res 2005; 14(3):695-703.	Diagnosis not fulfilling criteria
Depression	Hays RD, Sherbourne CD, Mazel RM. The RAND 36-Item Health Survey 1.0. Health Econ 1993; 2(3):217-227.	Multiple publication
Depression	Hays RD, Wells KB, Sherbourne CD, Rogers W, Spritzer K. Functioning	Follow-up

	and well-being outcomes of patients with depression compared with chronic general medical illnesses. Arch Gen Psychiatry 1995; 52(1):11-19.	duration ± > 20%
Depression	Hegarty K, Gunn J, Chondros P, Small R. Association between depression and abuse by partners of women attending general practice: descriptive, cross sectional survey. BMJ 2004; 328(7440):621-624.	Diagnosis not fulfilling criteria
Depression	Heiligenstein JH, Ware JE, Jr., Beusterien KM, Roback PJ, Andrejasich C, Tollefson GD. Acute effects of fluoxetine versus placebo on functional health and well-being in late-life depression. Int Psychogeriatr 1995; 7 Suppl:125-37, 1995.:-37.	Patients > 60 years
Depression	Hensley ML, Robson ME, Kauff ND, Korytowsky B, Castiel M, Ostroff J et al. Pre- and postmenopausal high-risk women undergoing screening for ovarian cancer: anxiety, risk perceptions, and quality of life. Gynecol Oncol 2003; 89(3):440-446.	Diagnosis not fulfilling criteria
Depression	Heymen S, Whitehead WE, Scarlett Y. Elevated beck depression inventory (Bdi) Scores predict biofeedback treatment failure for fecal incontinence and constipation. Digestive Disease Week Abstracts & Itinerary Planner 2003; Abstract No W15111.	Diagnosis not fulfilling criteria
Depression	Inder WJ, Prickett TCR, Mulder RT. Normal opioid tone and hypothalamic-pituitary-adrenal axis function in chronic fatigue syndrome despite marked functional impairment. Clin Endocrinol 2005; 62(3):343-348.	Diagnosis not fulfilling criteria
Depression	Irwin M, Pike J, Cole J, Oxman M. Effects of a Behavioral Intervention, Tai Chi Chih, on Varicella-Zoster Virus Specific Immunity and Health Functioning in Older Adults. Psychosom Med 2003;65(5):824-830.	Patients > 60 years
Depression	Isacson D, Bingefors K, von Knorring L. The impact of depression is unevenly distributed in the population. Eur Psychiatry 2005; 20(3):205-212.	No follow-up data
Depression	Jackson-Triche ME, Greer SJ, Wells KB, Rogers W, Camp P, Mazel R. Depression and health-related quality of life in ethnic minorities seeking care in general medical settings. J Affect Disord 2000; 58(2):89-97.	Multiple publication
Depression	Janssens AC, Van Doorn PA, de Boer JB, Van Der Meche FG, Passchier J, Hintzen RQ. Impact of recently diagnosed multiple sclerosis on quality of life, anxiety, depression and distress of patients and partners. Acta Neurol Scand 2003; 108(6):389-395.	Diagnosis not fulfilling criteria
Depression	Janssens AC, Van Doorn PA, de Boer JB, Kalkers NF, Van Der Meche FG, Passchier J et al. Anxiety and depression influence the relation between disability status and quality of life in multiple sclerosis. Mult Scler 2003; 9(4):397-403.	Diagnosis not fulfilling criteria
Depression	Jarema M, Konieczynska Z, Glowczak M, Szaniawska A, Meder J, Jakubiak A. [The evaluation of subjective quality of life in patients with schizophrenia or depression]. [Polish]. Psychiatr Pol 1995; 29(5):641-653.	Diagnosis not fulfilling criteria
Depression	Jensen IB, Bergstrom G, Ljungquist T, Bodin L. A 3-year follow-up of a multidisciplinary rehabilitation programme for back and neck pain. Pain 2005; 115(3):273-283.	Follow-up duration ± > 20%
Depression	Jones R, Yates WR, Williams S, Zhou M, Hardman L. Outcome for adjustment disorder with depressed mood: comparison with other mood disorders. J Affect Disord 1999; 55(1):55-61.	Other
Depression	Judd F, Komiti A, Chua P, Mijch A, Hoy J, Grech P et al. Nature of depression in patients with HIV/AIDS. Aust N Z J Psychiatry 2005; 39(9):826-832.	Diagnosis not fulfilling criteria
Depression	Juergensen PH, Wuerth DB, Finkelstein SH, Kliger AS, Finkelstein FO. Comparison of the beck depression inventory (BDI), SF-36 and patient self assessed quality of life (paQoL) in CPD patients. J Am Soc Nephrol 2001; 12(Program and Abstract Issue):332A-333A.	Diagnosis not fulfilling criteria
Depression	Kaholokula JK, Haynes SN, Grandinetti A, Chang HK. Biological,	Diagnosis not

	psychosocial, and sociodemographic variables associated with depressive symptoms in persons with type 2 diabetes. J Behav Med 2003; 26(5):435-458.	fulfilling criteria
Depression	Karp JF, Weiner D, Seligman K, Butters M, Miller M, Frank E et al. Body pain and treatment response in late-life depression. Am J Geriatr Psychiatry 2005; 13(3):188-194.	Patients > 60 years
Depression	Katzelnick DJ, Kobak KA, Greist JH, Jefferson JW, Henk HJ. Effect of primary care treatment of depression on service use by patients with high medical expenditures. Psychiatr Serv 1997; 48(1):59-64.	SF-36 data without means
Depression	Keller MB, McCullough JP, Klein DN, Arnow B, Dunner DL, Gelenberg AJ et al. A comparison of nefazodone, the cognitive behavioral-analysis system of psychotherapy, and their combination for the treatment of chronic depression. N Engl J Med 2000; 342(20):1462-1470.	Multiple publication
Depression	Keller SD, Bayliss MS, Ware JE, Hsu MA, Damiano AM, Goss TF. Comparison of responses to SF-36 Health Survey questions with one-week and four-week recall periods. Health Serv Res 1997; 32(3):367-384.	Multiple publication
Depression	Kenealy PM, Beaumont GJ, Lintern T, Murrell R. Autobiographical memory, depression and quality of life in multiple sclerosis. J Clin Exp Neuropsychol 2000; 22(1):125-131.	Diagnosis not fulfilling criteria
Depression	King M, Davidson O, Taylor F, Haines A, Sharp D, Turner R. Effectiveness of teaching general practitioners skills in brief cognitive behaviour therapy to treat patients with depression: randomised controlled trial. BMJ 2002; 324(7343):947-950.	No SF-36 data
Depression	Klerman GL. Depressive disorders. Further evidence for increased medical morbidity and impairment of social functioning. Arch Gen Psychiatry 1989; 46(9):856-858.	No SF-36 data
Depression	Kollner V, Einsle F, Schade I, Maulhardt T, Gulielmos V, Joraschky P. [The influence of anxiety, depression and post traumatic stress disorder on quality of life after thoracic organ transplantation]. [German]. Z Psychosom Med Psychother 2003; 49(3):262-274.	Diagnosis not fulfilling criteria
Depression	Kongsakon R. The functioning and quality of life of depressive patients with 12 weeks of psychiatric care. J Med Assoc Thai 2005; 88(9):1261-1266.	SF-36 data without means
Depression	Kressin N, Spiro A, Skinner K. Negative Affectivity and Health-Related Quality of Life. Med Care August 2000;38(8):858-867 2000;(8):858-867.	No follow-up data
Depression	Kruijshaar ME, Hoeymans N, Bijl RV, Spijker J, Essink-Bot ML. Levels of disability in major depression: findings from the Netherlands Mental Health Survey and Incidence Study (NEMESIS). J Affect Disord 2003; 77(1):53-64.	No follow-up data
Depression	Lacasse Y, Rousseau L, Maltais F. Prevalence of depressive symptoms and depression in patients with severe oxygen-dependent chronic obstructive pulmonary disease. J Cardiopulm Rehabil 2001; 21(2):80-86.	Diagnosis not fulfilling criteria
Depression	Ladwig KH, Marten-Mittag B, Baumert J, Lowel H, Doring A, Investigators KORA. Case-finding for depressive and exhausted mood in the general population: reliability and validity of a symptom-driven diagnostic scale. Results from the prospective MONICA/KORA Augsburg Study. Ann Epidemiol 2004; 14(5):332-338.	No follow-up data
Depression	Larsson K, Sundberg HM, Karlbom U, Nordin K, Anderberg UM, Loof L. A group-based patient education programme for high-anxiety patients with Crohn disease or ulcerative colitis. Scand J Gastroenterol 2003; 38(7):763-769.	Diagnosis not fulfilling criteria
Depression	Loberiza FR, Jr., Rizzo JD, Bredeson CN, Horowitz MM, Weeks JC, Lee SJ. Depressive syndrome and early deaths following stem cell transplantation for hematologic diseases. Blood 2001; 98(11 Part 1):742a.	Diagnosis not fulfilling criteria
Depression	Loberiza FR, Jr., Rizzo JD, Bredeson CN, Antin JH, Horowitz MM,	Diagnosis not

	Weeks JC et al. Association of depressive syndrome and early deaths among patients after stem-cell transplantation for malignant diseases. J Clin Oncol 2002; 20(8):2118-2126.	fulfilling criteria
Depression	Lonnqvist J, Sintonen H, Syvalahti E, Appelberg B, Koskinen T, Mannikko T et al. Antidepressant efficacy and quality of life in depression: a double-blind study with moclobemide and fluoxetine. Acta Psychiatr Scand 1994; 89(6):363-369.	No SF-36 data
Depression	Lonnqvist J, Sihvo S, Syvalahti E, Sintonen H, Kiviruusu O, Pitkanen H. Moclobemide and fluoxetine in the prevention of relapses following acute treatment of depression. Acta Psychiatr Scand 1995; 91(3):189-194.	No SF-36 data
Depression	Lubetkin EI, Jia H, Gold MR. Depression, anxiety, and associated health status in low-income Chinese patients. Am J Prev Med 2003; 24(4):354-360.	No follow-up data
Depression	Lyons KS, Stewart BJ, Archbold PG, Carter JH, Perrin NA. Pessimism and optimism as early warning signs for compromised health for caregivers of patients with Parkinson's disease. Nurs Res 2004; 53(6):354-362.	Diagnosis not fulfilling criteria
Depression	MacPherson H, Thorpe L, Thomas K, Geddes D. Acupuncture for depression: first steps toward a clinical evaluation. J Altern Complement Med 2004; 10(6):1083-1091.	Cohort with Cohort with < 20 patients
Depression	Madero AD, Aragon MV. Paroxetine, fluoxetine, and sertraline are equally effective in treating depression in primary care. Evidence-Based Healthcare 2002; 6(2):85-86.	Follow-up duration ± > 20%
Depression	Mahendra N. Exercise and behavioural management training improves physical health and reduces depression in people with Alzheimer's disease. Evidence-Based Healthcare 2004; 8(2):77-79.	Diagnosis not fulfilling criteria
Depression	McCall NT, Parks P, Smith K, Pope G, Griggs M. The prevalence of major depression or dysthymia among aged Medicare Fee-for-Service beneficiaries. Int J Geriatr Psychiatry 2002; 17(6):557-565.	Patients > 60 years
Depression	McHorney CA, Kosinski M, Ware JE, Jr. Comparisons of the costs and quality of norms for the SF-36 health survey collected by mail versus telephone interview: results from a national survey. Med Care 1994; 32(6):551-567.	No follow-up data
Depression	McHorney CA, Ware JE, Jr., Lu JF, Sherbourne CD. The MOS 36-item Short-Form Health Survey (SF-36): III. Tests of data quality, scaling assumptions, and reliability across diverse patient groups. Med Care 1994; 32(1):40-66.	No follow-up data
Depression	Mckee MD, Cunningham M, Jankowski KR, Zayas L. Health-related functional status in pregnancy: relationship to depression and social support in a multi-ethnic population. Obstet Gynecol 2001; 97(6):988-993.	Diagnosis not fulfilling criteria
Depression	Merkelbach S, Sittinger H, Koenig J. Is there a differential impact of fatigue and physical disability on quality of life in multiple sclerosis? J Nerv Ment Dis 2002; 190(6):388-393.	Diagnosis not fulfilling criteria
Depression	Miner CM, Brown EB, Gonzales JS, Munir R. Switching patients from daily citalopram, paroxetine, or sertraline to once-weekly fluoxetine in the maintenance of response for depression. J Clin Psychiatry 2002; 63(3):232-240.	SF-36 data without means
Depression	Muller J, Kemmler G, Wissel J, Schneider A, Voller B, Grossmann J et al. The impact of blepharospasm and cervical dystonia on health-related quality of life and depression. J Neurol 2002; 249(7):842-846.	Diagnosis not fulfilling criteria
Depression	Mulsan BH, Schulberg HC, Schulz R. Major depression in older primary care patients conference abstract. 11th Annual Meeting of the American Association for Geriatric Psychiatry San Diego, California, USA 8th-11th March, 1998.	Patients > 60 years
Depression	Nayak RA, Madnani DM, Ward J. Risk of clinical depression in arthritis: A web-based co-administration of cesd-20 and sf-36 to a nationally	Diagnosis not fulfilling

	representative elderly sample. Value in Health 2002; 5(3):183.	criteria
Depression	Nease DE, Klinkman MS, Volk RJ. Improved detection of depression in primary care through severity evaluation. J Fam Pract 2002; 51(12):1065-1070.	No follow-up data
Depression	Nichols GA, Brown JB. Following Depression in Primary Care: Do Family Practice Physicians Ask About Depression at Different Rates Than Internal Medicine Physicians? Arch Fam Med 2000; 9(5):478-482.	No follow-up data
Depression	Oslin DW, Streim J, Katz IR, Edell WS, TenHave T. Change in disability follows inpatient treatment for late life depression. J Am Geriatr Soc 2000; 48(4):357-362.	Patients > 60 years
Depression	Park JM, Han SH. [The effect of exercise program on health and depression in the elderly]. [Korean]. Daehan Ganho Haghoeji 2003; 33(2):220-227.	Patients > 60 years
Depression	Paschalides C, Wearden AJ, Dunkerley R, Bundy C, Davies R, Dickens CM. The associations of anxiety, depression and personal illness representations with glycaemic control and health-related quality of life in patients with type 2 diabetes mellitus. J Psychosom Res 2004; 57(6):557-564.	Diagnosis not fulfilling criteria
Depression	Patti F, Cacopardo M, Palermo F, Ciancio MR, Lopes R, Restivo D et al. Health-related quality of life and depression in an Italian sample of multiple sclerosis patients. J Neurol Sci 2003; 211(1-2):55-62.	Diagnosis not fulfilling criteria
Depression	Peng YS, Chiang CK, Kao TW, Hung KY, Lu CS, Chiang SS et al. Sexual dysfunction in female hemodialysis patients: A multicenter study. Kidney Intl 2005; 68(2):760-765.	Diagnosis not fulfilling criteria
Depression	Peveler R, George C, Kinmonth AL, Campbell M, Thompson C. Effect of antidepressant drug counselling and information leaflets on adherence to drug treatment in primary care: randomised controlled trial. BMJ 1999; 319(7210):612-615.	Diagnosis not fulfilling criteria
Depression	Philipp M, Kohnen R, Hiller KO. Hypericum extract versus imipramine or placebo in patients with moderate depression: randomised multicentre study of treatment for eight weeks. BMJ 1999; 319(7224):1534-1538.	No baseline SF-36 data
Depression	Pihl E, Jacobsson A, Fridlund B, Stromberg A, Martensson J. Depression and health-related quality of life in elderly patients suffering from heart failure and their spouses: a comparative study. Eur J Heart Fail 2005; 7(4):583-589.	Diagnosis not fulfilling criteria
Depression	Powers JR, Young AF, Russell A, Pachana NA. Implications of non-response of older women to a short form of the Center for Epidemiologic Studies Depression Scale. Int J Aging Hum Dev 2003; 57(1):37-54.	Patients > 60 years
Depression	Prochaska JJ, Sorensen JL, Hall SM, Rossi JS, Redding CA, Rosen AB et al. Predictors of health functioning in two high-risk groups of smokers. Drug Alcohol Depend 2005; 78(2):169-175.	No follow-up data
Depression	Pukrop R, Schlaak V, Moller-Leimkuhler AM, Albus M, Czernik A, Klosterkotter J et al. Reliability and validity of Quality of Life assessed by the Short-Form 36 and the Modular System for Quality of Life in patients with schizophrenia and patients with depression. Psychiatry Res 2003; 119(1-2):63-79.	SF-36 data without means
Depression	Pyne JM, Rost KM, Zhang M, Williams DK, Smith J, Fortney J. Cost-effectiveness of a primary care depression intervention. J Gen Intern Med 2003; 18(6):432-441.	No SF-36 data
Depression	Rapaport MH, Judd LL. Minor depressive disorder and subsyndromal depressive symptoms: functional impairment and response to treatment. J Affect Disord 1998; 48(2-3):227-232.	No follow-up data
Depression	Rocchi S, Scalvini S, Boraso A, Ghidelli C, Giordano A. [Anxiety, depression, and stressed life in patients awaiting heart transplantation: are these factors to be considered?]. [Italian]. Monaldi Arch Chest Dis 2004; 62(1):1-6.	Diagnosis not fulfilling criteria

Depression	Rogers WH, Adler DA, Bungay KM, Wilson IB. Depression screening instruments made good severity measures in a cross-sectional analysis. J Clin Epidemiol 2005; 58(4):370-377.	No follow-up data
Depression	Rost K, Nutting P, Smith JL, Elliott CE, Dickinson M. Managing depression as a chronic disease: a randomised trial of ongoing treatment in primary care. BMJ 2002; 325(7370):934.	SF-36 data without means
Depression	Rothenhausler HB, Ehrentraut S, Kapfhammer HP, Lang C, Zachoval R, Bilzer M et al. Psychiatric and psychosocial outcome of orthotopic liver transplantation. Psychother Psychosom 2002; 71(5):285-297.	Diagnosis not fulfilling criteria
Depression	Rowan PJP, Al Jurdi RM, Tavakoli-Tabasi SM, Kunik MEMM*, Satrom SLB, El Serag HBM. Physical and Psychosocial Contributors to Quality of Life in Veterans With Hepatitis C Not on Antiviral Therapy. J Clin Gastroenterol 2005; 39(8):731-736.	Diagnosis not fulfilling criteria
Depression	Rudolph M, Kummer P, Eysholdt U, Rosanowski F. [Speech impaired children. Anxiety, depression and quality of life of the mothers]. [German]. HNO 2004; 52(6):561-568.	Diagnosis not fulfilling criteria
Depression	Rush AJ, Koran LM, Keller MB, Markowitz JC, Harrison WM, Miceli RJ et al. The treatment of chronic depression, part 1: study design and rationale for evaluating the comparative efficacy of sertraline and imipramine as acute, crossover, continuation, and maintenance phase therapies. J Clin Psychiatry 1998; 59(11):589-597.	Multiple publication
Depression	Russell JM, Koran LM, Rush J, Hirschfeld RM, Harrison W, Friedman ES et al. Effect of concurrent anxiety on response to sertraline and imipramine in patients with chronic depression. Depress Anxiety 2001; 13(1):18-27.	No follow-up data
Depression	Salsberry PJ, Nickel JT, Polivka BJ, Kuthy RA, Slack C, Shapiro N. Self-reported health status of low-income mothers. Image J Nurs Sch 1999; 31(4):375-380.	No SF-36 data
Depression	Sapin C, Fantino B, Nowicki ML, Kind P. Usefulness of EQ-5D in assessing health status in primary care patients with major depressive disorder. Health Qual Life Outcomes 2004; 2(1):20.	Follow-up duration ± > 20%
Depression	Schneider B, Varghese RK. Scores on the SF-36 scales and the Beck Depression Inventory in assessing mental health among patients on hemodialysis. Psychol Rep 1995; 76(3 Pt 1):719-722.	Diagnosis not fulfilling criteria
Depression	Schulberg HC, Coulehan JL, Block MR, Lave J, Rodriguez E, Scott CP et al. Clinical trials of primary care treatments for major depression: issues in design, recruitment and treatment. Int J Psychiatry Med 1993; 23(1):29-42.	Multiple publication
Depression	Sewitch MJ, McCusker J, Dendukuri N, Yaffe MJ. Depression in frail elders: impact on family caregivers. Int J Geriatr Psychiatry 2004; 19(7):655-665.	Patients > 60 years
Depression	Shanahan CW, Lincoln A, Horton NJ, Saitz R, Winter M, Samet JH. Relationship of depressive symptoms and mental health functioning to repeat detoxification. J Subst Abuse Treat 2005; 29(2):117-123.	Diagnosis not fulfilling criteria
Depression	Sheikh J. Medical comorbidity and treatment response in an elderly depression study. 14th Annual Meeting of the American Association for Geriatric Psychiatry; 2001; San Francisco, CA, USA 2001.	Patients > 60 years
Depression	Sherbourne CD, Hays RD, Wells KB. Personal and psychosocial risk factors for physical and mental health outcomes and course of depression among depressed patients. J Consult Clin Psychol 1995; 63(3):345-355.	No SF-36 data
Depression	Sherbourne CD, Wells KB, Meredith LS, Jackson CA, Camp P. Comorbid anxiety disorder and the functioning and well-being of chronically ill patients of general medical providers. Arch Gen Psychiatry 1996; 53(10):889-895.	Follow-up duration ± > 20%
Depression	Sherbourne CD, Wells KB, Sturm R. Measuring health outcomes for	Multiple

	depression. Eval Health Prof 1997; 20(1):47-64.	publication
Depression	Sherbourne CD, Unutzer J, Schoenbaum M, Duan N, Lenert LA, Sturm R et al. Can utility-weighted health-related quality-of-life estimates capture health effects of quality improvement for depression? Med Care 2001; 39(11):1246-1259.	No SF-36 data
Depression	Shi L, Namjoshi MA, Swindle R, Yu X, Risser R, Baker RW et al. Effects of olanzapine alone and olanzapine/fluoxetine combination on health-related quality of life in patients with bipolar depression: secondary analyses of a double-blind, placebo-controlled, randomized clinical trial. Clin Ther 2004; 26(1):125-134.	Diagnosis not fulfilling criteria
Depression	Shuldham C, Goodman H, Fleming S, Tattersall K, Pryse-Hawkins H. Anxiety, depression and functional capacity in older women with mitral valve stenosis. Int J Nurs Pract 2001; 7(5):322-328.	Diagnosis not fulfilling criteria
Depression	Sierra P, Livianos L, Rojo L. Quality of life for patients with bipolar disorder: relationship with clinical and demographic variables. Bipolar Disord 2005; 7(2):159-165.	No follow-up data
Depression	Silveira E, Taft C, Sundh V, Waern M, Palsson S, Steen B. Performance of the SF-36 health survey in screening for depressive and anxiety disorders in an elderly female Swedish population. Qual Life Res 2005; 14(5):1263-1274.	Multiple publication
Depression	Simon GE, von Korff M, Heiligenstein JH, Revicki DA, Grothaus L, Katon W et al. Initial antidepressant choice in primary care. Effectiveness and cost of fluoxetine vs tricyclic antidepressants. JAMA 1996; 275(24):1897-1902.	Multiple publication
Depression	Simon GE, von Korff M, Lin E. Clinical and functional outcomes of depression treatment in patients with and without chronic medical illness. Psychol Med 2005; 35(2):271-279.	Patients > 60 years
Depression	Skobel EC, Sinha AM, Norra C, Randerath W, Breithardt OA, Breuer C et al. Effect of cardiac resynchronization therapy on sleep quality, quality of life, and symptomatic depression in patients with chronic heart failure and Cheyne-Stokes respiration. Sleep Breath 2005; 9(4):159-166.	Diagnosis not fulfilling criteria
Depression	Skodol AE, Grilo CM, Pagano ME, Bender DS, Gunderson JG, Shea MT et al. Effects of personality disorders on functioning and well-being in major depressive disorder. J Psychiatr Pract 2005; 11(6):363-368.	No follow-up data
Depression	Small GW, Birkett M, Meyers BS, Koran LM, Bystritsky A, Nemeroff CB. Impact of physical illness on quality of life and antidepressant response in geriatric major depression. Fluoxetine Collaborative Study Group. J Am Geriatr Soc 1996; 44(10):1220-1225.	Patients > 60 years
Depression	Small R, Lumley J, Donohue L, Potter A, Waldenstrom U. Randomised controlled trial of midwife led debriefing to reduce maternal depression after operative childbirth. BMJ 2000; 321(7268):1043-1047.	Diagnosis not fulfilling criteria
Depression	Small R, Lumley J, Yelland J. How useful is the concept of somatization in cross-cultural studies of maternal depression? A contribution from the Mothers in a New Country (MINC) study. J Psychosom Obstet Gynaecol 2003; 24(1):45-52.	Diagnosis not fulfilling criteria
Depression	Smith GC, Trauer T, Kerr PG, Chadban SJ. Prospective psychosocial monitoring of living kidney donors using the Short Form-36 Health Survey: Results at 12 months. Transplantation 2004; 78(9):1384-1389.	Multiple publication
Depression	Smith JL, Rost KM, Nutting PA, Elliott CE. Resolving disparities in antidepressant treatment and quality-of-life outcomes between uninsured and insured primary care patients with depression. Med Care 2001; 39(9):910-922.	Diagnosis not fulfilling criteria
Depression	Souetre E, Martin P, Lozet H, Monteban H. Quality of life in depressed patients: comparison of fluoxetine and major tricyclic antidepressants. Int Clin Psychopharmacol 1996; 11(1):45-52.	No baseline SF-36 data
Depression	Spijker J, Graaf R, Bijl RV, Beekman AT, Ormel J, Nolen WA. Functional disability and depression in the general population. Results from the	Multiple publication

	Netherlands Mental Health Survey and Incidence Study (NEMESIS). Acta Psychiatr Scand 2004; 110(3):208-214.	
Depression	Stansfeld SA, Head J, Fuhrer R, Wardle J, Cattell V. Social inequalities in depressive symptoms and physical functioning in the Whitehall II study: exploring a common cause explanation. J Epidemiol Community Health 2003; 57(5):361-367.	SF-36 data without means
Depression	Stein MB, Barrett-Connor E. Quality of life in older adults receiving medications for anxiety, depression, or insomnia: findings from a community-based study. Am J Geriatr Psychiatry 2002; 10(5):568-574.	Patients > 60 years
Depression	Stoll T, Kauer Y, Buchi S, Klaghofer R, Sensky T, Villiger PM. Prediction of depression in systemic lupus erythematosus patients using SF-36 Mental Health scores. Rheumatology 2001; 40(6):695-698.	Diagnosis not fulfilling criteria
Depression	Suenkeler IH, Nowak M, Misselwitz B, Kugler C, Schreiber W, Oertel WH et al. Timecourse of health-related quality of life as determined 3, 6 and 12 months after stroke. Relationship to neurological deficit, disability and depression. J Neurol 2002; 249(9):1160-1167.	Diagnosis not fulfilling criteria
Depression	Sullivan M, Levy WC, Russo JE, Spertus JA. Depression and health status in patients with advanced heart failure: a prospective study in tertiary care. J Card Fail 2004; 10(5):390-396.	Diagnosis not fulfilling criteria
Depression	Swenson JR, O'Connor CM, Barton D, Van Zyl LT, Swedberg K, Forman LM et al. Influence of depression and effect of treatment with sertraline on quality of life after hospitalization for acute coronary syndrome. Am J Cardiol 2003; 92(11):1271-1276.	Diagnosis not fulfilling criteria
Depression	Szaflarski JP, Szaflarski M. Seizure disorders, depression, and health-related quality of life. Epilepsy Behav 2004; 5(1):50-57.	Diagnosis not fulfilling criteria
Depression	Taskapan H, Ates F, Kaya B, Emul M, Kaya M, Taskapan C et al. Psychiatric disorders and large interdialytic weight gain in patients on chronic haemodialysis. Nephrology 2005; 10(1):15-20.	Diagnosis not fulfilling criteria
Depression	Tate D, Paul RH, Flanigan TP, Tashima K, Nash J, Adair C et al. The impact of apathy and depression on quality of life in patients infected with HIV. AIDS Patient Care STDS 2003; 17(3):115-120.	Diagnosis not fulfilling criteria
Depression	Thompson C, Peveler RC, Stephenson D, McKendrick J. Compliance with antidepressant medication in the treatment of major depressive disorder in primary care: a randomized comparison of fluoxetine and a tricyclic antidepressant. Am J Psychiatry 2000; 157(3):338-343.	SF-36 data without means
Depression	Thunedborg K, Black CH, Bech P. Beyond the Hamilton depression scores in long-term treatment of manic-melancholic patients: prediction of recurrence of depression by quality of life measurements. Psychother Psychosom 1995; 64(3-4):131-140.	Diagnosis not fulfilling criteria
Depression	Toso V, Gandolfo C, Paolucci S, Provinciali L, Torta R, Grassivaro N et al. Post-stroke depression: research methodology of a large multicentre observational study (DESTRO). Neurol Sci 2004; 25(3):138-144.	Diagnosis not fulfilling criteria
Depression	Trumper A, Appleby L. Psychiatric morbidity in patients undergoing heart, heart and lung, or lung transplantation. J Psychosom Res 2001; 50(2):103-105.	Diagnosis not fulfilling criteria
Depression	Tylee A, Gastpar M, Lepine JP, Mendlewicz J. Identification of depressed patient types in the community and their treatment needs: findings from the DEPRES II (Depression Research in European Society II) survey. DEPRES Steering Committee. Int Clin Psychopharmacol 1999; 14(3):153-165.	No SF-36 data
Depression	Uguz S, Inanc BY, Yerlikaya EE, Aydin H. [Reliability and validity of Turkish form of Endicott Work Productivity Scale]. [Turkish]. Turk Psikiyatri Derg 2004; 15(3):209-214.	Language not fulfilling criteria
Depression	Vage V, Solhaug JH, Viste A, Bergsholm P, Wahl AK. Anxiety, depression and health-related quality of life after jejunoileal bypass: a 25-year follow-up study of 20 female patients. Obes Surg 2003; 13(5):706-	Diagnosis not fulfilling criteria

	713.	
Depression	van der Schaaf I, Brilstra EH, Rinkel GJ, Bossuyt PM, van Gijn J. Quality of life, anxiety, and depression in patients with an untreated intracranial aneurysm or arteriovenous malformation. Stroke 2002; 33(2):440-443.	Diagnosis not fulfilling criteria
Depression	Velanovich V. The effect of chronic pain syndromes and psychoemotional disorders on symptomatic and quality-of-life outcomes of antireflux surgery. J Gastrointest Surg 2003; 7(1):53-58.	Diagnosis not fulfilling criteria
Depression	Vernay D, Gerbaud L, Biolay S, Coste J, Debourse J, Aufauvre D et al. [Quality of life and multiple sclerosis: validation of the french version of the self-questionnaire (SEP-59)]. [French]. Rev Neurol (Paris) 2000; 156(3):247-263.	Diagnosis not fulfilling criteria
Depression	Volk RJ, Cantor SB, Steinbauer JR, Cass AR. Alcohol use disorders, consumption patterns, and health-related quality of life of primary care patients. Alcohol Clin Exp Res 1997; 21(5):899-905.	Diagnosis not fulfilling criteria
Depression	von Korff M, Ormel J, Katon W, Lin EH. Disability and depression among high utilizers of health care. A longitudinal analysis. Arch Gen Psychiatry 1992; 49(2):91-100.	No SF-36 data
Depression	von Korff M, Ustun TB, Ormel J, Kaplan I, Simon GE. Self-report disability in an international primary care study of psychological illness. J Clin Epidemiol 1996; 49(3):297-303.	No SF-36 data
Depression	von Korff M, Katon W, Rutter C, Ludman E, Simon G, Lin E et al. Effect on disability outcomes of a depression relapse prevention program. Psychosom Med 2003; 65(6):938-943.	Other
Depression	Walker V, Streiner DL, Novosel S, Rocchi A, Levine MA, Dean DM. Health-related quality of life in patients with major depression who are treated with moclobemide. J Clin Psychopharmacol 1995; 15(4 Suppl 2):60S-67S.	No follow-up data
Depression	Walters BA, Hays RD, Spritzer KL, Fridman M, Carter WB. Health-related quality of life, depressive symptoms, anemia, and malnutrition at hemodialysis initiation. Am J Kidney Dis 2002; 40(6):1185-1194.	Diagnosis not fulfilling criteria
Depression	Ware JE, Jr., Kosinski M, Bayliss MS, McHorney CA, Rogers WH, Raczek A. Comparison of methods for the scoring and statistical analysis of SF-36 health profile and summary measures: summary of results from the Medical Outcomes Study. Med Care 1995; 33(4 Suppl):AS264-AS279.	Multiple publication
Depression	Ware JE, Jr., Bayliss MS, Rogers WH, Kosinski M, Tarlov AR. Differences in 4-year health outcomes for elderly and poor, chronically ill patients treated in HMO and fee-for-service systems. Results from the Medical Outcomes Study. JAMA 1996; 276(13):1039-1047.	Patients > 60 years
Depression	Wells KB, Stewart A, Hays RD, Burnam MA, Rogers W, Daniels M et al. The functioning and well-being of depressed patients. Results from the Medical Outcomes Study. JAMA 1989; 262(7):914-919.	Multiple publication
Depression	Wells KB. Caring for depression in primary care: defining and illustrating the policy context. J Clin Psychiatry 1997; 58 Suppl 1:24-7:24-27.	Multiple publication
Depression	Williams JW, Barrett J, Oxman T, Frank E, Katon W, Sullivan M et al. Treatment of dysthymia and minor depression in primary care: A randomized controlled trial in older adults. JAMA 2000; 284(12):1519-1526.	Patients > 60 years
Depression	Williams JW, Jr., Kerber CA, Mulrow CD, Medina A, Aguilar C. Depressive disorders in primary care: prevalence, functional disability, and identification. J Gen Intern Med 1995; 10(1):7-12.	Patients > 60 years
Depression	Willrich A, Pinzur M, McNeil M, Juknelis D, Lavery L. Health related quality of life, cognitive function, and depression in diabetic patients with foot ulcer or amputation. A preliminary study. Foot Ankle Int 2005; 26(2):128-134.	Diagnosis not fulfilling criteria
Depression	Xavier FM, Ferraza MP, Argimon I, Trentini CM, Poyares D, Bertollucci	Patients > 60

	PH et al. The DSM-IV 'minor depression' disorder in the oldest-old: prevalence rate, sleep patterns, memory function and quality of life in elderly people of Italian descent in Southern Brazil. Int J Geriatr Psychiatry 2002; 17(2):107-116.	years
Depression	Yamazaki S, Fukuhara S, Green J. Usefulness of five-item and three-item Mental Health Inventories to screen for depressive symptoms in the general population of Japan. Health Qual Life Outcomes 2005; 3:48, 2005 Aug 8.	No follow-up data
Depression	Yatham LN, Lecrubier Y, Fieve RR, Davis KH, Harris SD, Krishnan AA. Quality of life in patients with bipolar I depression: data from 920 patients. Bipolar Disord 2004; 6(5):379-385.	Diagnosis not fulfilling criteria
Depression	Young M, Stuber J, Ahern J, Galea S. Interpersonal discrimination and the health of illicit drug users. Am J Drug Alcohol Abuse 2005; 31(3):371-391.	Diagnosis not fulfilling criteria
Low back pain	Abresch RT, Carter GT, Jensen MP, Kilmer DD. Assessment of pain and health-related quality of life in slowly progressive neuromuscular disease. Am J Hosp Palliat Care 2002; 19(1):39-48.	Diagnosis not fulfilling criteria
Low back pain	Al Smadi J, Warke K, Wilson I, Cramp AF, Noble G, Walsh DM et al. A pilot investigation of the hypoalgesic effects of transcutaneous electrical nerve stimulation upon low back pain in people with multiple sclerosis. Clin Rehabil 2003; 17(7):742-749.	Diagnosis not fulfilling criteria
Low back pain	Albert HB, Jensen AM, Dahl D, Rasmussen MN. [Criteria validation of the Roland Morris questionnaire. A Danish translation of the international scale for the assessment of functional level in patients with low back pain and sciatica]. [Danish]. Ugeskr Laeger 2003; 165(18):1875-1880.	Follow-up duration ± > 20%
Low back pain	Albert TJ, Purtill J, Mesa J, McIntosh T, Balderston RA. Health outcome assessment before and after adult deformity surgery. A prospective study. Spine 1995; 20(18):2002-2004.	No follow-up data
Low back pain	Albert TJ, Mesa JJ, Eng K, McIntosh TC, Balderston RA. Health outcome assessment before and after lumbar laminectomy for radiculopathy. Spine 1996; 21(8):960-962.	Diagnosis not fulfilling criteria
Low back pain	Anagnostis C, Gatchel RJ, Mayer TG. The pain disability questionnaire: a new psychometrically sound measure for chronic musculoskeletal disorders. Spine 2004; 29(20):2290-2302.	Diagnosis not fulfilling criteria
Low back pain	Andersen JH, Kaergaard A, Frost P, Thomsen JF, Bonde JP, Fallentin N et al. Physical, psychosocial, and individual risk factors for neck/shoulder pain with pressure tenderness in the muscles among workers performing monotonous, repetitive work. Spine 2002; 27(6):660-667.	Diagnosis not fulfilling criteria
Low back pain	Baker JG, Fiedler RC, Ottenbacher KJ, Czyrny JJ, Heinemann AW. Predicting follow-up functional outcomes in outpatient rehabilitation. Am J Phys Med Rehabil 1998; 77(3):202-212.	Diagnosis not fulfilling criteria
Low back pain	Baraniuk JN, Whalen G, Cunningham J, Clauw DJ. Cerebrospinal fluid levels of opioid peptides in fibromyalgia and chronic low back pain. BMC Musculoskelet Disord 2004; 5(1):48.	No follow-up data
Low back pain	Beaton DE, Bombardier C, Hogg-Johnson SA. Measuring health in injured workers: a cross-sectional comparison of five generic health status instruments in workers with musculoskeletal injuries. Am J Ind Med 1996; 29(6):618-631.	SF-36 acute form only
Low back pain	Beaton DE, Hogg-Johnson S, Bombardier C. Evaluating changes in health status: reliability and responsiveness of five generic health status measures in workers with musculoskeletal disorders. J Clin Epidemiol 1997; 50(1):79-93.	SF-36 acute form only
Low back pain	Bennett RMM, Schein JD, Kosinski MRM, Hewitt DJM, Jordan DMB, Rosenthal NRM. Impact of Fibromyalgia Pain on Health-Related Quality of Life Before and After Treatment With Tramadol/Acetaminophen. Arthritis Rheum 2005; 53(4):519-527.	Diagnosis not fulfilling criteria
Low back	Beutler WJ, Fredrickson BE, Murtland A, Sweeney CA, Grant WD, Baker	Diagnosis not

pain	D. The natural history of spondylolysis and spondylolisthesis: 45-year follow-up evaluation. Spine 2003; 28(10):1027-1035.	fulfilling criteria
Low back pain	Birrell FN, Hassell AB, Jones PW, Dawes PT. How does the short form 36 health questionnaire (SF-36) in rheumatoid arthritis (RA) relate to RA outcome measures and SF-36 population values? A cross-sectional study. Clin Rheumatol 2000; 19(3):195-199.	Diagnosis not fulfilling criteria
Low back pain	Boden SD, Kang J, Sandhu H, Heller JG. Use of recombinant human bone morphogenetic protein-2 to achieve posterolateral lumbar spine fusion in humans: a prospective, randomized clinical pilot trial: 2002 Volvo Award in clinical studies. Spine 2002; 27(23):2662-2673.	Cohort with Cohort with < 20 patients
Low back pain	Bombardier C. Outcome assessments in the evaluation of treatment of spinal disorders: summary and general recommendations. Spine 2000; 25(24):3100-3103.	No SF-36 data
Low back pain	Boucher M, Bhandari M, Kwok D. Health-related quality of life after short segment instrumentation of lumbar burst fractures. J Spinal Disord 2001; 14(5):417-426.	Diagnosis not fulfilling criteria
Low back pain	Bronfort G, Bouter LM. Responsiveness of general health status in chronic low back pain: a comparison of the COOP charts and the SF-36. Pain 1999; 83(2):201-209.	Modified SF-36
Low back pain	Brown J, Klapow J, Doleys D, Lowery D, Tutak U. Disease-specific and generic health outcomes: a model for the evaluation of long-term intrathecal opioid therapy in noncancer low back pain patients. Clin J Pain 1999; 15(2):122-131.	No baseline SF-36 data
Low back pain	Buchowski JM, Kebaish KM, Sinkov V, Cohen DB, Sieber AN, Kostuik JP. Functional and radiographic outcome of sacroiliac arthrodesis for the disorders of the sacroiliac joint. Spine J 2005; 5(5):520-528.	Cohort with < 20 patients
Low back pain	Bullinger M, Kirchberger I. SF-36 Fragebogen zum Gesundheitszustand. Handanweisung. Göttingen: Hogrefe-Verlag, 1998.	No follow-up data
Low back pain	Cakir B, Ulmar B, Koepp H, Huch K, Puhl W, Richter M. [Posterior dynamic stabilization as an alternative for dorso-ventral fusion in spinal stenosis with degenerative instability]. [German]. Z Orthop Ihre Grenzgeb 2003; 141(4):418-424.	Diagnosis not fulfilling criteria
Low back pain	Canadian European Study Group. Double-blind comparison of full and partial anemia correction in incident hemodialysis patients without symptomatic heart disease. J Am Soc Nephrol 2005; 16(7):2180-2189.	Diagnosis not fulfilling criteria
Low back pain	Chernysheva TV, Bagirova GG. [Midocalm in complex therapy of chronic low back pain syndrome]. [Russian]. Klin Med (Mosk) 2005; 83(11):45-49.	Follow-up duration ± > 20%
Low back pain	Colloca CJ, Keller TS. Stiffness and neuromuscular reflex response of the human spine to posteroanterior manipulative thrusts in patients with low back pain. J Manipulative Physiol Ther 2001; 24(8):489-500.	No follow-up data
Low back pain	Coste J, Lefrancois G, Guillemin F, Pouchot J, - French Study Group for Quality of Life in Rheumatology. Prognosis and quality of life in patients with acute low back pain: insights from a comprehensive inception cohort study. Arthritis Rheum 2004; 51(2):168-176.	Diagnosis not fulfilling criteria
Low back pain	Cote P, Cassidy JD, Carroll L. The treatment of neck and low back pain: who seeks care? who goes where? Med Care 2001; 39(9):956-967.	Diagnosis not fulfilling criteria
Low back pain	Coulter ID, Hurwitz EL, Adams AH, Genovese BJ, Hays R, Shekelle PG. Patients using chiropractors in North America: who are they, and why are they in chiropractic care? Spine 2002; 27(3):291-296.	Diagnosis not fulfilling criteria
Low back pain	Dahl B, Gehrchen PM, Kiaer T, Blyme P, Tondevold E, Bendix T. Nonorganic pain drawings are associated with low psychological scores on the preoperative SF-36 questionnaire in patients with chronic low back pain. Eur Spine J 2001; 10(3):211-214.	No follow-up data
Low back	Dallolio V. Lumbar spinal decompression with a pneumatic orthesis	No SF-36 data

pain	(Orthotrac): preliminary study. Acta Neurochir Suppl 2005; 92:133-7, 2005.:-7.	
Low back pain	Davidson M, Keating JL. A comparison of five low back disability questionnaires: reliability and responsiveness. Phys Ther 2002; 82(1):8-24.	No follow-up data
Low back pain	Davidson M, Keating JL, Eyres S. A low back-specific version of the SF-36 Physical Functioning scale. Spine 2004; 29(5):586-594.	No follow-up data
Low back pain	Derebery MJ, Berliner KI. Allergy and health-related quality of life. Otolaryngol Head Neck Surg 2000; 123(4):393-399.	Diagnosis not fulfilling criteria
Low back pain	Douglas TS, Mann NH, Hodge AL. Evaluation of preoperative patient education and computer-assisted patient instruction. J Spinal Disord 1998; 11(1):29-35.	No baseline SF-36 data
Low back pain	Ekman P, Moller H, Hedlund R. The long-term effect of posterolateral fusion in adult isthmic spondylolisthesis: a randomized controlled study. Spine J 2005; 5(1):36-44.	Diagnosis not fulfilling criteria
Low back pain	Engbers LH, Vollenbroek-Hutten MM, van Harten WH. A comparison of patient characteristics and rehabilitation treatment content of chronic low back pain (CLBP) and stroke patients across six European countries. Health Policy 2005; 71(3):359-373.	No follow-up data
Low back pain	Epstein NE. Lumbar laminectomy for the resection of synovial cysts and coexisting lumbar spinal stenosis or degenerative spondylolisthesis: an outcome study. Spine 2004; 29(9):1049-1055.	Diagnosis not fulfilling criteria
Low back pain	Ernst ME, Doucette WR, Dedhiya SD, Osterhaus MC, Kumbera PA, Osterhaus JT et al. Use of point-of-service health status assessments by community pharmacists to identify and resolve drug-related problems in patients with musculoskeletal disorders. Pharmacotherapy 2001; 21(8):988-997.	Diagnosis not fulfilling criteria
Low back pain	Ernst ME, Iyer SS, Doucette WR. Drug-related problems and quality of life in arthritis and low back pain sufferers. Value Health 2003; 6(1):51-58.	SF-36 data without means
Low back pain	Ewert T, Fuessl M, Cieza A, Andersen C, Chatterji S, Kostanjsek N et al. Identification of the most common patient problems in patients with chronic conditions using the ICF checklist. J Rehabil Med 2004;(44 Suppl):22-29.	No follow-up data
Low back pain	Fairbank J, Frost H, Wilson-MacDonald J, Yu LM, Barker K, Collins R et al. Randomised controlled trial to compare surgical stabilisation of the lumbar spine with an intensive rehabilitation programme for patients with chronic low back pain: the MRC spine stabilisation trial. BMJ 2005; 330(7502):1233.	Follow-up duration ± > 20%
Low back pain	Fanuele JC, Abdu WA, Hanscom B, Weinstein JN. Association between obesity and functional status in patients with spine disease. Spine 2002; 27(3):306-312.	Diagnosis not fulfilling criteria
Low back pain	Foran JRH, Pyeritz RE, Dietz HC, Sponseller PD. Characterization of the symptoms associated with dural ectasia in the Marfan patient. Am J Med Genet 2005; 134A(1):58-65.	Diagnosis not fulfilling criteria
Low back pain	Fritz JM, Delitto A, Erhard RE. Comparison of classification-based physical therapy with therapy based on clinical practice guidelines for patients with acute low back pain: a randomized clinical trial. Spine 2003; 28(13):1363-1371.	Diagnosis not fulfilling criteria
Low back pain	Gage H, Hendricks A, Zhang S, Kazis L. The relative health related quality of life of veterans with Parkinson's disease. J Neurol Neurosurg Psychiatry 2003; 74(2):163-169.	Diagnosis not fulfilling criteria
Low back pain	Gallagher RM, Mossey JM. (209) impact of co-morbid depression on self-reported pain and physical and emotional functioning in low back pain patients. Pain Med 2001; 2(3):242.	No SF-36 data

Low back pain	Garratt AM, Ruta DA, Abdalla MI, Buckingham JK, Russell IT. The SF36 health survey questionnaire: an outcome measure suitable for routine use within the NHS? Br Med J 1993; 306(6890):1440-1444.	Multiple publication
Low back pain	Gatchel RJ, Polatin PB, Mayer TG, Robinson R, Dersh J. Use of the SF-36 Health Status Survey with a chronically disabled back pain population: Strengths and limitations. J Occup Rehabil 1998; 8:237-245.	No follow-up data
Low back pain	Gatchel RJ, Mayer T, Dersh J, Robinson R, Polatin P. The association of the SF-36 health status survey with 1-year socioeconomic outcomes in a chronically disabled spinal disorder population. Spine 1999; 24(20):2162-2170.	Multiple publication
Low back pain	Ghoname EA, Craig WF, White PF, Ahmed HE, Hamza MA, Henderson BN et al. Percutaneous electrical nerve stimulation for low back pain: a randomized crossover study. JAMA 1999; 281(9):818-823.	Multiple publication
Low back pain	Ghoname ES, Craig WF, White PF, Ahmed HE, Hamza MA, Gajraj NM et al. The effect of stimulus frequency on the analgesic response to percutaneous electrical nerve stimulation in patients with chronic low back pain. Anesth Analg 1999; 88(4):841-846.	Multiple publication
Low back pain	Gilbert FJ, Grant AM, Gillan MG, Vale LD, Campbell MK, Scott NW et al. Low back pain: influence of early MR imaging or CT on treatment and outcome--multicenter randomized trial. Radiology 2004; 231(2):343-351.	Follow-up duration ± > 20%
Low back pain	Glassman SD, Minkow RE, Dimar JR, Puno RM, Raque GH, Johnson JR. Effect of prior lumbar discectomy on outcome of lumbar fusion: a prospective analysis using the SF-36 measure. J Spinal Disord 1998; 11(5):383-388.	Diagnosis not fulfilling criteria
Low back pain	Goetze C, Slomka A, Goetze H, G, Poetzl W, Liljenqvist U et al. [Long-term results of quality of life in patients with idiopathic scoliosis after Harrington instrumentation and their relevance for expert evidence]. [German] Z Orthop Ihre Grenzgeb 2002;(5): 492-498.	Diagnosis not fulfilling criteria
Low back pain	Gotze C, Liljenqvist UR, Slomka A, Gotze HG, Steinbeck J. Quality of life and back pain: outcome 16.7 years after Harrington instrumentation. Spine 2002; 27(13):1456-1463.	Diagnosis not fulfilling criteria
Low back pain	Grevitt M, Khazim R, Webb J, Mulholland R, Shepperd J. The short form-36 health survey questionnaire in spine surgery. J Bone Joint Surg Br 1997; 79(1):48-52.	No baseline SF-36 data
Low back pain	Grevitt MP, McLaren A, Shackleford IM, Mulholland RC. Automated percutaneous lumbar discectomy. An outcome study. J Bone Joint Surg Br 1995; 77(4):626-629.	No baseline SF-36 data
Low back pain	Grotle M, Brox JI, Vollestad NK. Cross-cultural adaptation of the Norwegian versions of the Roland-Morris Disability Questionnaire and the Oswestry Disability Index. J Rehabil Med 2003; 35(5):241-247.	Multiple publication
Low back pain	Haas M, Groupp E, Muench J, Kraemer D, Brummel-Smith K, Sharma R et al. Chronic disease self-management program for low back pain in the elderly. J Manipulative Physiol Ther 2005; 28(4):228-237.	Patients > 60 years
Low back pain	Hamza MA, Ghoname EA, White PF, Craig WF, Ahmed HE, Gajraj NM et al. Effect of the duration of electrical stimulation on the analgesic response in patients with low back pain. Anesthesiology 1999; 91(6):1622-1627.	No follow-up data
Low back pain	Hanly JG, Mitchell M, MacMillan L, Mosher D, Sutton E. Efficacy of sacroiliac corticosteroid injections in patients with inflammatory spondyloarthropathy: results of a 6 month controlled study. J Rheumatol 2000; 27(3):719-722.	Diagnosis not fulfilling criteria
Low back pain	Hanscom B, Lurie JD, Homa K, Weinstein JN. Computerized questionnaires and the quality of survey data. Spine 2002; 27(16):1797-1801.	No follow-up data
Low back pain	Hartvigsen J, Lauridsen H, Ekstrom S, Nielsen MB, Lange F, Kofoed N et al. Translation and validation of the danish version of the Bournemouth questionnaire. J Manipulative Physiol Ther 2005;	No follow-up data

	28(6):402-407.	
Low back pain	Hee HT, Whitecloud TS, III, Myers L, Gaynor J, Roesch W, Ricciardi JE. SF-36 health status of workers compensation cases with spinal disorders. Spine J 2001; 1(3):176-182.	Diagnosis not fulfilling criteria
Low back pain	Hee HT, Whitecloud TS, Myers L. The Effect of Previous Low Back Surgery on General Health Status: Results From the National Spine Network Initial Visit Survey of Patients With Low Back Pain. Spine 2004; 29(17):1931-1937.	No follow-up data
Low back pain	Hodselmans AP, Jaegers SM, Goeken LN. Short-term outcomes of a back school program for chronic low back pain. Arch Phys Med Rehabil 2001; 82(8):1099-1105.	Follow-up duration ± > 20%
Low back pain	Hollingworth W, Deyo RA, Sullivan SD, Emerson SS, Gray DT, Jarvik JG. The practicality and validity of directly elicited and SF-36 derived health state preferences in patients with low back pain. Health Econ 2002; 11(1):71-85.	No SF-36 data
Low back pain	Hoopmann M, Reichle C, Krauth C, Schwartz FW, Walter U. [Effect of a back education program by the Lower Saxony AOK in response to the development of health related quality of life and occupational disability]. [German]. Gesundheitswesen 2001; 63(3):176-182.	Multiple publication
Low back pain	Hozack WJ, Rothman RH, Albert TJ, Balderston RA, Eng K. Relationship of total hip arthroplasty outcomes to other orthopaedic procedures. Clin Orthop 1997;(344):88-93.	No follow-up data
Low back pain	Hutchinson PJ, Laing RJ, Waran V, Hutchinson E, Hollingworth W. Assessing outcome in lumbar disc surgery using patient completed measures. Br J Neurosurg 2000; 14(3):195-199.	No baseline SF-36 data
Low back pain	Indahl A, Velund L, Reikeraas O. Good prognosis for low back pain when left untampered. A randomized clinical trial. Spine 1995; 20(4):473-477.	No SF-36 data
Low back pain	Iversen MD, Fossel AH, Katz JN. Enhancing function in older adults with chronic low back pain: a pilot study of endurance training. Arch Phys Med Rehabil 2003; 84(9):1324-1331.	Patients > 60 years
Low back pain	Jay TC, Jones SL, Coe N, Breen AC. A chiropractic service arrangement for musculoskeletal complaints in industry: a pilot study. Occup Med (Lond) 1998; 48(6):389-395.	Diagnosis not fulfilling criteria
Low back pain	Jensen IB, Bergstrom G, Ljungquist T, Bodin L, Nygren AL. A randomized controlled component analysis of a behavioral medicine rehabilitation program for chronic spinal pain: are the effects dependent on gender? Pain 2001; 91(1-2):65-78.	Diagnosis not fulfilling criteria
Low back pain	Jette DU, Jette AM. Physical therapy and health outcomes in patients with spinal impairments. Phys Ther 1996; 76(9):930-941.	SF-36 data without means
Low back pain	Kapural L, Mekhail N. Radiofrequency ablation for chronic pain control. Curr Pain Headache Rep 2001; 5(6):517-525.	No SF-36 data
Low back pain	Keller S, Bann CM, Dodd SL, Schein J, Mendoza TR, Cleeland CS. Validity of the brief pain inventory for use in documenting the outcomes of patients with noncancer pain. Clin J Pain 2004; 20(5):309-318.	SF-36 data without means
Low back pain	Kjoller M, Rasmussen NK, Keiding LM. Selvrapportered sundhed og sygelighed blandt voksne danskere 1987-1994. Ugeskr Laeger 1999; 161(20):2948-2954.	No SF-36 data
Low back pain	Koch KD, Buchanan R, Birch JG, Morton AA, Gatchel RJ, Browne RH. Adolescents undergoing surgery for idiopathic scoliosis: how physical and psychological characteristics relate to patient satisfaction with the cosmetic result. Spine 2001; 26(19):2119-2124.	Diagnosis not fulfilling criteria
Low back pain	Koestler ME, Libby E, Schofferman J, Redmond T. Web-based touch-screen computer assessment of chronic low back pain: a pilot study. Comput Inform Nurs 2005; 23(5):275-284.	No follow-up data
Low back pain	Kopec JA, Esdaile JM, Abrahamowicz M, Abenhaim L, Wood-Dauphinee S, Lamping DL et al. The Quebec Back Pain Disability Scale.	No follow-up data

	Measurement properties. Spine 1995; 20(3):341-352.	
Low back pain	Korovessis P, Dimas A, Lambiris E. The significance of correlation of radiographic variables and MOS short-form health survey for clinical decision in symptomatic low back pain patients. Stud Health Technol Inform 2002; 91:325-331.	No follow-up data
Low back pain	Korovessis P, Dimas A, Iliopoulos P, Lambiris E. Correlative analysis of lateral vertebral radiographic variables and medical outcomes study short-form health survey: a comparative study in asymptomatic volunteers versus patients with low back pain. J Spinal Disord Tech 2002; 15(5):384-390.	No follow-up data
Low back pain	Korovessis P, Papazisis Z, Koureas G, Lambiris E. Rigid, semirigid versus dynamic instrumentation for degenerative lumbar spinal stenosis: a correlative radiological and clinical analysis of short-term results. Spine 2004; 29(7):735-742.	Diagnosis not fulfilling criteria
Low back pain	Krause TM. Case management through a multidisciplinary spinal evaluation. Orthop Nurs 1997; 16(2 Suppl):46-50.	Diagnosis not fulfilling criteria
Low back pain	Krousel-Wood MA, McCune TW, Abdoh A, Re RN. Predicting work status for patients in an occupational medicine setting who report back pain. Arch Fam Med 1994; 3(4):349-355.	No follow-up data
Low back pain	Kumar MN, Jacquot F, Hall H. Long-term follow-up of functional outcomes and radiographic changes at adjacent levels following lumbar spine fusion for degenerative disc disease. Eur Spine J 2001; 10(4):309-313.	Diagnosis not fulfilling criteria
Low back pain	Lang E. [Structure quality of independent physicians for pain management - is there a standard?]. [German]. Schmerz 2000; 14(4):226-230.	No SF-36 data
Low back pain	Lansky D, Butler JB, Waller FT. Using health status measures in the hospital setting: from acute care to 'outcomes management'. Med Care 1992; 30(5 Suppl):MS57-MS73.	Diagnosis not fulfilling criteria
Low back pain	Laursen BS, Bajaj P, Olesen AS, Delmar C, Arendt-Nielsen L. Health related quality of life and quantitative pain measurement in females with chronic non-malignant pain. Eur J Pain 2005; 9(3):267-275.	Cohort with < 20 patients
Low back pain	Lean ME, Han TS, Seidell JC. Impairment of health and quality of life using new US federal guidelines for the identification of obesity. Arch Intern Med 1999; 159(8):837-843.	SF-36 data without means
Low back pain	Lettice JJ, Kula TA, Derby R, Kim BJ, Lee SH, Seo KS. Does the number of levels affect lumbar fusion outcome? Spine 2005; 30(6):675-681.	Multiple publication
Low back pain	Leung AS, Lam TH, Hedley AJ, Twomey LT. Use of a subjective health measure on Chinese low back pain patients in Hong Kong. Spine 1999; 24(10):961-966.	No SF-36 data
Low back pain	Levy HI, Hanscom B, Boden SD. Three-question depression screener used for lumbar disc herniations and spinal stenosis. Spine 2002; 27(11):1232-1237.	No follow-up data
Low back pain	Licciardone JC, Stoll ST, Fulda KG, Russo DP, Siu J, Winn W et al. Osteopathic manipulative treatment for chronic low back pain: a randomized controlled trial. Spine 2003; 28(13):1355-1362.	SF-36 data without means
Low back pain	Linhardt O, Kruger A, Krodel A. [Differences between anteroposterior and posterior spondylodesis in clinical scores]. [German]. Unfallchirurg 2004; 107(7):593-600.	Diagnosis not fulfilling criteria
Low back pain	Lorish TR, Tanabe CT, Waller FT, London MR, Lansky DJ. Correlation between health outcome and length of hospital stay in lumbar microdiscectomy. Spine 1998; 23(20):2195-2200.	Diagnosis not fulfilling criteria
Low back pain	Lyle MA, Manes S, McGuinness M, Ziaei S, Iversen MD. Relationship of physical examination findings and self-reported symptom severity and physical function in patients with degenerative lumbar conditions. Phys	No follow-up data

	Ther 2005; 85(2):120-133.	
Low back pain	Mau M, Merkesdal S, Busche T, Bauer J. [Prognosis of labour force participation after multidisciplinary outpatient and inpatient rehabilitation for chronic back pain]. [German]. Rehabilitation (Stuttg) 2002; 41(2-3):160-166.	SF-36 data without means
Low back pain	McGregor AH, Hughes SP. The evaluation of the surgical management of nerve root compression in patients with low back pain: Part 1: the assessment of outcome. Spine 2002; 27(13):1465-1470.	Diagnosis not fulfilling criteria
Low back pain	McKenna PJ, Freeman BJ, Mulholland RC, Grevitt MP, Webb JK, Mehdian SH. A prospective, randomised controlled trial of femoral ring allograft versus a titanium cage in circumferential lumbar spinal fusion with minimum 2-year clinical results. Eur Spine J 2005; 14(8):727-737.	Follow-up duration ± > 20%
Low back pain	McKinnon ME, Vickers MR, Ruddock VM, Townsend J, Meade TW. Community studies of the health service implications of low back pain. Spine 1997; 22(18):2161-2166.	No follow-up data
Low back pain	McPherson K, Britton A. The impact of patient treatment preferences on the interpretation of randomised controlled trials. Eur J Cancer 1999; 35(11):1598-1602.	Cohort with < 20 patients
Low back pain	Mehling WE, Hamel KA, Acree M, Byl N, Hecht FM. Randomized, controlled trial of breath therapy for patients with chronic low-back pain. Altern Ther Health Med 2005; 11(4):44-52.	Cohort with < 20 patients
Low back pain	Menefee LA, Frank ED, Doghramji K, Picarello K, Park JJ, Jalali S et al. Self-reported sleep quality and quality of life for individuals with chronic pain conditions. Clin J Pain 2000; 16(4):290-297.	No follow-up data
Low back pain	Merkesdal S, Bernitt K, Bräuer W, Busche T, Bauer I, Mau W. Vergleich des sechsmonatigen Verlaufs nach stationärer und teilstationärer Rehabilitation an Hand des SF-36 Health Survey bei Patienten mit Dorsopathien. [German]. In: Verband Deutscher Rentenversicherungsträger, editor. 9. Rehabilitationswissenschaftliches Kolloquium: Individualität und Reha-Prozess vom 13. bis 15. März in Würzburg. Frankfurt/Main: VDR, 2000: 279-280.	No baseline SF-36 data
Low back pain	Morken T, Riise T, Moen B, Bergum O, Hauge SH, Holien S et al. Frequent musculoskeletal symptoms and reduced health-related quality of life among industrial workers. Occup Med (Lond) 2002; 52(2):91-98.	No follow-up data
Low back pain	Nguyen HV, Ludwig S, Gelb D. Osteoporotic vertebral burst fractures with neurologic compromise. J Spinal Disord Tech 2003; 16(1):10-19.	Diagnosis not fulfilling criteria
Low back pain	Nickel R, Egle uT, Eysel P, Rompe JD, Zollner J, Hoffmann SO. Health-related quality of life and somatization in patients with long-term low back pain: a prospective study with 109 patients. Spine 2001; 26(20):2271-2277.	SF-36 data without means
Low back pain	Nickel R, Egle uT, Rompe J, Eysel P, Hoffmann SO. Somatisation predicts the outcome of treatment in patients with low back pain. J Bone Joint Surg Br 2002; 84(2):189-195.	SF-36 data without means
Low back pain	Nickel R, Egle UT. [Predictors of quality of life after orthopedic treatment of lower back pain due to lumbar intervertebral disc disorders]. [German]. Z Psychosom Med Psychother 2003; 49(1):49-62.	SF-36 data without means
Low back pain	Nork SE, Hu SS, Workman KL, Glazer PA, Bradford DS. Patient outcomes after decompression and instrumented posterior spinal fusion for degenerative spondylolisthesis. Spine 1999; 24(6):561-569.	Diagnosis not fulfilling criteria
Low back pain	Oh WS, Shim JC. A randomized controlled trial of radiofrequency denervation of the ramus communicans nerve for chronic discogenic low back pain. Clin J Pain 2004; 20(1):55-60.	Follow-up duration ± > 20%
Low back pain	Osterhaus JT, Townsend RJ, Gandek B, Ware JE. Measuring the functional status and well-being of patients with migraine headache. Headache 1994; 34(6):337-343.	No follow-up data

Low back pain	Osterhaus JT, Dedhiya SD, Ernst ME, Osterhaus M, Mehta SS, Townsend RJ. Health outcomes assessment in community pharmacy practices: a feasibility project. Arthritis Rheum 2002; 47(2):124-131.	No follow-up data
Low back pain	Padua R, Padua S, Aulisa L, Ceccarelli E, Padua L, Romanini E et al. Patient outcomes after Harrington instrumentation for idiopathic scoliosis: a 15- to 28-year evaluation. Spine 2001; 26(11):1268-1273.	Diagnosis not fulfilling criteria
Low back pain	Palangio M, Morris E, Doyle RT, Jr., Dornseif BE, Valente TJ. Combination hydrocodone and ibuprofen versus combination oxycodone and acetaminophen in the treatment of moderate or severe acute low back pain. Clin Ther 2002; 24(1):87-99.	Diagnosis not fulfilling criteria
Low back pain	Pauza KJ, Howell S, Dreyfuss P, Peloza JH, Dawson K, Bogduk N. A randomized, placebo-controlled trial of intradiscal electrothermal therapy for the treatment of discogenic low back pain. Spine J 2004; 4(1):27-35.	No follow-up data
Low back pain	Pither C. Is lumbar spinal surgery more effective than intensive rehabilitation for management of low back pain? Nat Clin Pract Rheumatol 2005; 1(1):20-21.	Follow-up duration ± > 20%
Low back pain	Porchet F, Wietlisbach V, Burnand B, Daeppen K, Villemure JG, Vader JP. Relationship between severity of lumbar disc disease and disability scores in sciatica patients. Neurosurgery 2002; 50(6):1253-1259.	No follow-up data
Low back pain	Raak R, Wikblad K, Raak A, Sr., Carlsson M, Wahren LK. Catastrophizing and health-related quality of life: a 6-year follow-up of patients with chronic low back pain. Rehabil Nurs 2002; 27(3):110-116.	No follow-up data
Low back pain	Ren XS, Kazis L, Lee A, Miller DR, Clark JA, Skinner K et al. Comparing generic and disease-specific measures of physical and role functioning: results from the Veterans Health Study. Med Care 1997; 36(2):155-166.	Diagnosis not fulfilling criteria
Low back pain	Ren XS, Selim AJ, Fincke G, Deyo RA, Linzer M, Lee A et al. Assessment of functional status, low back disability, and use of diagnostic imaging in patients with low back pain and radiating leg pain. J Clin Epidemiol 1999; 52(11):1063-1071.	No follow-up data
Low back pain	Ren XS, Kazis LE, Lee A, Rogers WH. The role of generic and disease-specific measures of physical and role functioning in assessing patient outcomes: a longitudinal study. J Ambul Care Manage 2005; 28(2):157-166.	Multiple publication
Low back pain	Resnik L, Dobrzykowski E. Guide to outcomes measurement for patients with low back pain syndromes. J Orthop Sports Phys Ther 2003; 33(6):307-316.	No SF-36 data
Low back pain	Resnik L, Dobrykowski E. Outcomes measurement for patients with low back pain. Orthop Nurs 2005; 24(1):14-24.	No follow-up data
Low back pain	Revicki DA, Wood M. Patient-assigned health state utilities for depression-related outcomes: differences by depression severity and antidepressant medications. J Affect Disord 1998; 48(1):25-36.	No follow-up data
Low back pain	Riddle DL, Lee KT, Stratford PW. Use of SF-36 and SF-12 health status measures: a quantitative comparison for groups versus individual patients. Med Care 2001; 39(8):867-878.	No follow-up data
Low back pain	Ruta DA, Garratt AM, Leng M, Russell IT, MacDonald LM. A new approach to the measurement of quality of life. The Patient-Generated Index. Med Care 1994; 32(11):1109-1126.	Multiple publication
Low back pain	Ruta DA, Garratt AM, Wardlaw D, Russell IT. Developing a valid and reliable measure of health outcome for patients with low back pain. Spine 1994; 19(17):1887-1896.	Multiple publication
Low back pain	Ruta DA, Garratt AM, Russell IT. Patient centred assessment of quality of life for patients with four common conditions. Qual Health Care 1999; 8(1):22-29.	Multiple publication
Low back pain	Saal JS, Saal JA. Management of chronic discogenic low back pain with a thermal intradiscal catheter. A preliminary report. Spine 2000; 25(3):382-388.	Diagnosis not fulfilling criteria

Low back pain	Saal JSM, Saal JAM. Management of Chronic Discogenic Low Back Pain With a Thermal Intradiscal Catheter: A Preliminary Report. Spine 2000; 25(3):382-388.	Multiple publication
Low back pain	Salaffi F, De Angelis R, Stancati A, Grassi W, MArche P, Prevalence INvestigation Group (. Health-related quality of life in multiple musculoskeletal conditions: a cross-sectional population based epidemiological study. II. The MAPPING study. Clin Exp Rheumatol 2005; 23(6):829-839.	No follow-up data
Low back pain	Sasso RC, Kitchel SH, Dawson EG. A prospective, randomized controlled clinical trial of anterior lumbar interbody fusion using a titanium cylindrical threaded fusion device. Spine 2004; 29(2):113-122.	Diagnosis not fulfilling criteria
Low back pain	Schultz IZ, Crook J, Berkowitz J, Milner R, Meloche GR. Predicting return to work after low back injury using the Psychosocial Risk for Occupational Disability Instrument: a validation study. J Occup Rehabil 2005; 15(3):365-376.	Diagnosis not fulfilling criteria
Low back pain	Schwab F, Dubey A, Pagala M, Gamez L, Farcy JP. Adult scoliosis: a health assessment analysis by SF-36. Spine 2003; 28(6):602-606.	Diagnosis not fulfilling criteria
Low back pain	Selim AJ, Fincke G, Ren XS, Deyo RA, Lee A, Skinner K et al. Patient characteristics and patterns of use for lumbar spine radiographs: results from the Veterans Health Study. Spine 2000; 25(19):2440-2444.	Multiple publication
Low back pain	Selim AJ, Fincke G, Ren XS, Deyo RA, Lee A, Skinner K et al. Racial differences in the use of lumbar spine radiographs: results from the Veterans Health Study. Spine 2001; 26(12):1364-1369.	Multiple publication
Low back pain	Selim AJ, Fincke G, Berlowitz DR, Miller DR, Qian SX, Lee A et al. Comprehensive health status assessment of centenarians: results from the 1999 large health survey of veteran enrollees. J Gerontol A Biol Sci Med Sci 2005; 60(4):515-519.	Patients > 60 years
Low back pain	Smith BH, Elliott AM, Hannaford PC, Chambers W. Factors related to the onset and persistence of chronic back pain in the community Results from a general population follow-up study. Spine 29(9) May 2004; 1,2004.1032-1040.	SF-36 data without means
Low back pain	Spine Stabilisation Trial Group. Randomised controlled trial to compare surgical stabilisation of the lumbar spine with an intensive rehabilitation programme for patients with chronic low back pain: the MRC spine stabilisation trial. BMJ 2005; 330(7502):1233-1239.	Multiple publication
Low back pain	Spruit M, Jacobs WC. Pain and function after intradiscal electrothermal treatment (IDET) for symptomatic lumbar disc degeneration. Eur Spine J 2002; 11(6):589-593.	Cohort with < 20 patients
Low back pain	Stucki G, Stucki S, Sangha O. [Patient-centered evaluation of illness outcome in musculoskeletal diseases: adaptation and revision of outcome instruments]. [German]. Z Rheumatol 1997; 56(5):266-275.	No SF-36 data
Low back pain	Swiontkowski MF, Engelberg R, Martin DP, Agel J. Short musculoskeletal function assessment questionnaire: validity, reliability, and responsiveness. J Bone Joint Surg Am 1999; 81(9):1245-1260.	Diagnosis not fulfilling criteria
Low back pain	Synnot A, Williams M. Low back pain in individuals with chronic airflow limitation and their partners--a preliminary prevalence study. Physiother Res Int 2002; 7(4):215-227.	Diagnosis not fulfilling criteria
Low back pain	Taylor SJ, Taylor AE, Foy MA, Fogg AJ. Responsiveness of common outcome measures for patients with low back pain. Spine 1999; 24(17):1805-1812.	SF-36 data without means
Low back pain	Thomas KJ, Fitter M, Brazier J, MacPherson H, Campbell M, Nicholl JP et al. Longer-term clinical and economic benefits of offering acupuncture to patients with chronic low back pain assessed as suitable for primary care management. Complement Ther Med 1999; 7(2):91-100.	Multiple publication
Low back	Underwood MR, Barnett AG, Vickers MR. Evaluation of two time-specific	No follow-up

pain	back pain outcome measures. Spine 1999; 24(11):1104-1112.	data
Low back pain	Vogt MT, Hanscom B, Lauerman WC, Kang JD. Influence of smoking on the health status of spinal patients: the National Spine Network database. Spine 2002; 27(3):313-319.	SF-36 data without means
Low back pain	Walsh DA, Radcliffe JC. Pain beliefs and perceived physical disability of patients with chronic low back pain. Pain 2002; 97(1-2):23-31.	SF-36 data without means
Low back pain	Walters BA, Hays RD, Spritzer KL, Fridman M, Carter WB. Health-related quality of life, depressive symptoms, anemia, and malnutrition at hemodialysis initiation. Am J Kidney Dis 2002; 40(6):1185-1194.	No follow-up data
Low back pain	Wenban AB, Nielsen MK. Chiropractic maintenance care and quality of life of a patient presenting with chronic low back pain. J Manipulative Physiol Ther 2005; 28(2):136-142.	Cohort with < 20 patients
Low back pain	White PF, Ghoname EA, Ahmed HE, Hamza MA, Craig WF, Vakharia AS. The effect of montage on the analgesic response to percutaneous neuromodulation therapy. Anesth Analg 2001; 92(2):483-487.	No follow-up data
Low back pain	Wiesinger GF, Nuhr M, Quittan M, Ebenbichler G, Wolfl G, Fialka-Moser V. Cross-cultural adaptation of the Roland-Morris questionnaire for German-speaking patients with low back pain. Spine 1999; 24(11):1099-1103.	No follow-up data
Low back pain	Wittink H, Rogers W, Gascon C, Sukiennik A, Cynn D, Carr DB. Relative contribution of mental health and exercise-related pain increment to treadmill test intolerance in patients with chronic low back pain. Spine 2001; 26(21):2368-2374.	Multiple publication
Low back pain	Wittink H, Rogers W, Sukiennik A, Carr DB. Physical functioning: self-report and performance measures are related but distinct. Spine 2003; 28(20):2407-2413.	No follow-up data
Low back pain	Wittink H, Turk D, Carr D, Sukiennik A, Rogers W. Comparison of the Redundancy, Reliability, and Responsiveness to Change Among SF-36, Oswestry Disability Index, and Multidimensional Pain Inventory. Clin J Pain 2004; 20(3):133-142.	Diagnosis not fulfilling criteria
Low back pain	Zwingmann C, Metzger D, Jäckel WH. [Short Form-36 health survey (SF-36): Psychometric analysis of the German version in rehabilitants of chronic lower back pain]. [German]. Diagnostica 1998; 44(4):209-219.	No follow-up data
Migraine	Aaronson NK, Muller M, Cohen PD, Essink-Bot ML, Fekkes M, Sanderman R et al. Translation, validation, and norming of the Dutch language version of the SF-36 Health Survey in community and chronic disease populations. J Clin Epidemiol 1998; 51(11):1055-1068.	No follow-up data
Migraine	Aksu S, Kurne A, Duger T, Oksuz C, Turk C, Gedizoglu T et al. Are results of the SF-36 health survey and the Nottingham health profile similar in migraine patients? The Pain Clinic 2004; 16(2):167-171.	No follow-up data
Migraine	Babiak LM, Miller DW, MacMillan JH, Sprang.G. Migraine-specific quality of life: a comparison of U.S. and Canadian results. Qual Life Res 1994; 3(58).	No SF-36 data
Migraine	Barton-Donovan K, Blanchard EB. Psychosocial aspects of chronic daily headache. J Headache Pain 2005; 6(1):30-39.	No follow-up data
Migraine	Becker PM, Schwartz JR, Feldman NT, Hughes RJ. Effect of modafinil on fatigue, mood, and health-related quality of life in patients with narcolepsy. Psychopharmacology 2004; 171(2):133-139.	No follow-up data
Migraine	Becker WJ. Assessing health-related quality of life in patients with migraine. Can J Neurol Sci 2002; 29(Suppl2):16-22.	Diagnosis not fulfilling criteria
Migraine	Betchen SA, Walsh J, Post KD. Self-assessed quality of life after acoustic neuroma surgery. J Neurosurg 2003; 99(5):818-823.	Diagnosis not fulfilling criteria
Migraine	Bingefors K, Isacson D. Epidemiology, co-morbidity, and impact on health-related quality of life of self-reported headache and	Diagnosis not fulfilling

	musculoskeletal pain--a gender perspective. Eur J Pain 2004; 8(5):435-450.	criteria
Migraine	Black DW, Okiishi C, Schlosser S. The Iowa follow-up of chemically sensitive persons. Ann N Y Acad Sci 2001; 933:48-56, 2001 Mar.:-56.	Diagnosis not fulfilling criteria
Migraine	Boline PD, Kassak K, Bronfort G, Nelson C, Anderson AV. Spinal manipulation vs. amitriptyline for the treatment of chronic tension-type headaches: a randomized clinical trial. J Manipulative Physiol Ther 1995; 18(3):148-154.	SF-36 data without means
Migraine	Boureau F, Chazot G, Emile J, Bertin L, d'Allens H. Comparison of subcutaneous sumatriptan with usual acute treatments for migraine. French Sumatriptan Study Group. Eur Neurol 1995; 35(5):264-269.	No SF-36 data
Migraine	Chang ST, Chen CL, Chen CC, Hung KC. Clinical events occurrence and the changes of quality of life in chronic haemodialysis patients with dry weight determined by echocardiographic method. Int J Clin Pract 2004; 58(12):1101-1107.	Diagnosis not fulfilling criteria
Migraine	Chatterton ML, Lofland JH, Shechter A, Curtice WS, Hu XH, Lenow J et al. Reliability and validity of the migraine therapy assessment questionnaire. Headache 2002; 42(10):1006-1015.	No follow-up data
Migraine	Cohen JA, Beall DG, Miller DW, Beck A, Pait G, Clements BD. Subcutaneous sumatriptan for the treatment of migraine: humanistic, economic, and clinical consequences. Fam Med 1996; 28(3):171-177.	Multiple publication
Migraine	Colas R, Munoz P, Temprano R, Gomez C, Pascual J. Chronic daily headache with analgesic overuse: epidemiology and impact on quality of life. Neurology 2004; 62(8):1338-1342.	Diagnosis not fulfilling criteria
Migraine	D'Amico D, Rigamonti A, Solari A, Leone M, Usai S, Grazzi L et al. Health-related quality of life in patients with cluster headache during active periods. Cephalalgia 2002; 22(10):818-821.	No SF-36 data
Migraine	Dahlof CG. Health-related quality of life under six months' treatment of migraine--an open clinic-based longitudinal study. Cephalalgia 1995; 15(5):414-422.	Diagnosis not fulfilling criteria
Migraine	Dowson AJ, D'Amico D, Tepper SJ, Baos V, Baudet F, Kilminster S. Identifying patients who require a change in their current acute migraine treatment: the Migraine Assessment of Current Therapy (Migraine-ACT) questionnaire. Neurol Sci 2004; 25(Suppl3):276-278.	Follow-up duration ± > 20%
Migraine	Durham CF, Dalton JA, Carlson J, Neelon V, Alden KR, Englebardt S. Migraine headache. J Am Acad Nurse Pract 1997; 9(4):179-185.	No SF-36 data
Migraine	Durham CF, Alden KR, Dalton JA, Carlson J, Miller DW, Englebardt SP et al. Quality of life and productivity in nurses reporting migraine. Headache 1998; 38(6):427-435.	No follow-up data
Migraine	Ertsey C, Manhalter N, Bozsik G, Afra J, Jelencsik I. Health-related and condition-specific quality of life in episodic cluster headache. Cephalalgia 2004; 24(3):188-196.	Diagnosis not fulfilling criteria
Migraine	Essink-Bot ML, van Royen L, Krabbe P, Bonsel GJ, Rutten FF. The impact of migraine on health status. Headache 1995; 35(4):200-206.	No follow-up data
Migraine	Essink-Bot ML, Krabbe PF, Bonsel GJ, Aaronson NK. An empirical comparison of four generic health status measures. The Nottingham Health Profile, the Medical Outcomes Study 36-item Short-Form Health Survey, the COOP/WONCA charts, and the EuroQol instrument. Med Care 1997; 35(5):522-537.	Multiple publication
Migraine	Frank L, Kleinman L, Rentz A, Ciesla G, Kim JJ, Zacker C. Health-related quality of life associated with irritable bowel syndrome: comparison with other chronic diseases. Clin Ther 2002; 24(4):675-689.	Diagnosis not fulfilling criteria
Migraine	Freitag FG, Kozma CM, Slaton T, Osterhaus JT, Barron R. Characterization and prediction of emergency department use in chronic daily headache patients. Headache 2005; 45(7):891-898.	No follow-up data

Migraine	Gerbaud L, Navez ML, Couratier P, Lejeune ML, Vernay D, Aufauvre D et al. [Validation of the combined SF36/MSQOL test of evaluation of quality of life in migraine patients in France]. [French]. Rev Neurol (Paris) 2002; 158(6-7):719-727.	No follow-up data
Migraine	Gerbershagen HU, Lindena G, Korb J, Kramer S. [Health-related quality of life in patients with chronic pain]. [German]. Schmerz 2002; 16(4):271-284.	No follow-up data
Migraine	Guitera V, Munoz P, Castillo J, Pascual J. Quality of life in chronic daily headache: a study in a general population. Neurology 2002; 58(7):1062-1065.	No follow-up data
Migraine	Harpole LH, Samsa GP, Jurgelski AE, Shipley JL, Bernstein A, Matchar DB. Headache management program improves outcome for chronic headache. Headache 2003; 43(7):715-724.	Diagnosis not fulfilling criteria
Migraine	Hernandez MR, Fernandez Lopez JA, Rancano G, I, Cueto EA. [Quality of life and neurologic diseases]. [Spanish]. Neurologia 2001; 16(1):30-37.	Diagnosis not fulfilling criteria
Migraine	Heywood J, Bouchard J, Cortelli P, Dahlof C, Jansen JP, Pham S et al. A multinational investigation of the impact of subcutaneous sumatriptan. I: Design, methods and clinical findings. Pharmacoeconomics 1997; 11 Suppl 1:11-23.	Multiple publication
Migraine	Holroyd KA, Stensland M, Lipchik GL, Hill KR, O'Donnell FS, Cordingley G. Psychosocial correlates and impact of chronic tension-type headaches. Headache 2000; 40(1):3-16.	No SF-36 data
Migraine	Kaegi L. Medical Outcomes Trust Conference presents dramatic advances in patient-based outcomes assessment and potential applications in accreditation. Jt Comm J Qual Improv 1999; 25(4):207-218.	No SF-36 data
Migraine	Kawata AK, Coeytaux RR, Devellis RF, Finkel AG, Mann JD, Kahn K. Psychometric properties of the HIT-6 among patients in a headache-specialty practice. Headache 2005; 45(6):638-643.	No follow-up data
Migraine	Kirchberger I, Bullinger M, Bruggenjurgen B. Quality of life in migraine treatment. Results of a longitudinal study using the SF-36 health survey. Qual Life Res 1994; 3:83.	No SF-36 data
Migraine	Lainez Andres JM. [Migraine and quality of life]. [Spanish]. Neurologia 1998; 13 Suppl 2:1-8, 1998 Oct.:-8.	No follow-up data
Migraine	Lang E, Kastner S, Neundorfer B, Bickel A. [Effects of recommendations and patient seminars on effectivity of outpatient treatment for headache]. [German]. Schmerz 2001; 15(4):229-240.	Multiple publication
Migraine	Lee PP, Spritzer K, Hays RD. The impact of blurred vision on functioning and well-being. Ophthalmology 1997; 104(3):390-396.	Diagnosis not fulfilling criteria
Migraine	Linzer M, Spitzer R, Kroenke K, Williams JB, Hahn S, Brody D et al. Gender, quality of life, and mental disorders in primary care: results from the PRIME-MD 1000 study. Am J Med 1996; 101(5):526-533.	No SF-36 data
Migraine	Lipton RB, Hamelsky SW, Kolodner KB, Steiner TJ, Stewart WF. Migraine, quality of life, and depression: A population-based case-control study. Neurology 2000; 55(5):629-635.	No SF-36 data
Migraine	Lipton RB, Liberman JN, Kolodner KB, Bigal ME, Dowson A, Stewart WF. Migraine headache disability and health-related quality-of-life: a population-based case-control study from England. Cephalalgia 2003; 23(6):441-450.	No follow-up data
Migraine	Litaker DG, Solomon GD, Genzen JR. Using pretreatment quality of life perceptions to predict response to sumatriptan in migraineurs. Headache 1997; 37(10):630-634.	No follow-up data
Migraine	Lyons RA, Temple JM, Evans D, Fone DL, Palmer SR. Acute health effects of the Sea Empress oil spill. J Epidemiol Community Health 1999;	Diagnosis not fulfilling

	53(5):306-310.	criteria
Migraine	Magnusson JE, Riess CM, Becker WJ. Effectiveness of a multidisciplinary treatment program for chronic daily headache. Can J Neurol Sci 2004; 31(1):72-79.	Diagnosis not fulfilling criteria
Migraine	Mannix LK, Solomon GD, Kippes CM, Kunkel RS. Impact of headache education program in the workplace. Neurology 1999; 53(4):868-871.	No follow-up data
Migraine	Mariano HS, Garbelini RP, Nogueria RC, Tafuri MJ, Gomes VG, Bordini CA et al. Migraine and quality of life: utilization of SF-36 in an employee population of a hospital in Brazil. Cephalalgia 2001; 21(4):306-307.	No SF-36 data
Migraine	Martin BC, Pathak DS, Sharfman MI, Adelman JU, Taylor F, Kwong WJ et al. Validity and reliability of the migraine-specific quality of life questionnaire (MSQ Version 2.1). Headache 2000; 40(3):204-215.	SF-36 data without means
Migraine	Meletiche DM, Lofland JH, Young WB. Quality-of-life differences between patients with episodic and transformed migraine. Headache 2001; 41(6):573-578.	No follow-up data
Migraine	Michel P, Dartigues JF, Lindoulsi A, Henry P. Loss of productivity and quality of life in migraine sufferers among French workers: results from the GAZEL cohort. Headache 1997; 37(2):71-78.	No follow-up data
Migraine	Money SR, Herd JA, Isaacsohn JL, Davidson M, Cutler B, Heckman J et al. Effect of cilostazol on walking distances in patients with intermittent claudication caused by peripheral vascular disease. J Vasc Surg 1998; 27(2):267-274.	Diagnosis not fulfilling criteria
Migraine	Monzon MJ, Lainez MJ. Quality of life in migraine and chronic daily headache patients. Cephalalgia 1998; 18(9):638-643.	No follow-up data
Migraine	Muscari-Tomaioli G, Allegri F, Miali E, Pomposelli R, Tubia P, Targhetta A et al. Observational study of quality of life in patients with headache, receiving homeopathic treatment. Br Homeopath J 2001; 90(4):189-197.	Diagnosis not fulfilling criteria
Migraine	Mushet GR, Miller D, Clements B, Pait G, Gutterman DL. Impact of sumatriptan on workplace productivity, nonwork activities, and health-related quality of life among hospital employees with migraine. Headache 1996; 36(3):137-143.	SF-36 data without means
Migraine	Nelson CF, Bronfort G, Evans R, Boline P, Goldsmith C, Anderson AV. The efficacy of spinal manipulation, amitriptyline and the combination of both therapies for the prophylaxis of migraine headache. J Manipulative Physiol Ther 1998; 21(8):511-519.	SF-36 data without means
Migraine	Nortvedt MW, Riise T, Myhr KM, Nyland HI, Hanestad BR. Type I interferons and the quality of life of multiple sclerosis patients. Results from a clinical trial on interferon alfa-2a. Mult Scler 1999; 5(5):317-322.	Diagnosis not fulfilling criteria
Migraine	Ohbu S, Igarashi H, Okayasu H, Sakai F, Green J, Heller RF et al. Development and testing of the Japanese version of the migraine-specific quality of life instrument. Qual Life Res 2004; 13(8):1489-1493.	No follow-up data
Migraine	Parfrey PS, Foley RN, Wittreich BH, Sullivan DJ, Zagari MJ, Frei D. Double-blind comparison of full and partial anemia correction in incident hemodialysis patients without symptomatic heart disease. J Am Soc Nephrol 2005; 16(7):2180-2189.	Diagnosis not fulfilling criteria
Migraine	Patrick DL, Martin ML, Bushmell DM, Pesa J. Measuring satisfaction with migraine treatment: expectations, importance, outcomes, and global ratings. Clin Ther 2003; 25(11):2920-2935.	SF-36 data without means
Migraine	Riess CM, Meckling SK, Rose MS, Becker WJ. Quality of life as measured by the SF-36 in migraine and transformed migraine patients. Cephalalgia 2000; 20(4):304.	No SF-36 data
Migraine	Rollnik JD, Karst M, Fink M, Dengler R. Coping strategies in episodic and chronic tension-type headache. Headache 2001; 41(3):297-302.	No SF-36 data
Migraine	Sabatowski R, Galvez R, Cherry DA, Jacquot F, Vincent E, Maisonobe P et al. Pregabalin reduces pain and improves sleep and mood disturbances in patients with post-herpetic neuralgia: results of a	Diagnosis not fulfilling criteria

	randomised, placebo-controlled clinical trial. Pain 2004; 109(1-2):26-35.	
Migraine	Schonfeld WH, Verboncoeur CJ, Fifer SK, Lipschutz RC, Lubeck DP, Buesching DP. The functioning and well-being of patients with unrecognized anxiety disorders and major depressive disorder. J Affect Disord 1997; 43(2):105-119.	Diagnosis not fulfilling criteria
Migraine	Shadick NA, Phillips CB, Sangha O, Logigian EL, Kaplan RF, Wright EA et al. Musculoskeletal and neurologic outcomes in patients with previously treated Lyme disease. Ann Intern Med 1999; 131(12):919-926.	Diagnosis not fulfilling criteria
Migraine	Si JM, Wang LJ, Chen SJ, Sun LM, Dai N. Irritable bowel syndrome consulters in Zhejiang province: the symptoms pattern, predominant bowel habit subgroups and quality of life. World J Gastroenterol 2004; 10(7):1059-1064.	Diagnosis not fulfilling criteria
Migraine	Silva HM, Jr., Garbelini RP, Teixeira SO, Bordini CA, Speciali JG. Effect of episodic tension-type headache on the health-related quality of life in employees of a Brazilian public hospital. Arq Neuropsiquiatr 2004; 62(3B):769-773.	No follow-up data
Migraine	Solomon GD, Skobieranda FG, Gragg LA. Quality of life and well-being of headache patients: measurement by the medical outcomes study instrument. Headache 1993; 33(7):351-358.	No follow-up data
Migraine	Solomon GD. Quality-of-life assessment in patients with headache. Pharmacoeconomics 1994; 6(1):34-41.	No SF-36 data
Migraine	Solomon GD, Skobieranda FG, Gragg LA. Does quality of life differ among headache diagnoses? Analysis using the Medical Outcomes Study instrument. Headache 1994; 34(3):143-147.	No SF-36 data
Migraine	Solomon GD. Evolution of the measurement of quality of life in migraine. Neurology 1997; 48(Suppl 3):S10-S15.	Multiple publication
Migraine	Terwindt GM, Ferrari MD, Tijhuis M, Groenen SMA, Picavet HSJ, Launer LJ. The impact of migraine on quality of life in the general population: The GEM study. Neurology 2000; 55(5):624-629.	Modified SF-36
Migraine	Toal CB, Mahon WA, Barnes C, Burelle D. Nifedipine gastrointestinal therapeutic system (GITS) for hypertensive patients in a primary care setting: results of the Extended Release Adalat Canadian Trial (EXACT). Clin Ther 1997; 19(5):924-935.	Diagnosis not fulfilling criteria
Migraine	Turner-Bowker DM, Bayliss MS, Ware JE, Jr., Kosinski M. Usefulness of the SF-8 Health Survey for comparing the impact of migraine and other conditions. Qual Life Res 2003; 12(8):1003-1012.	No SF-36 data
Migraine	Wagner TH, Patrick DL, Galer BS, Berzon RA. A new instrument to assess the long-term quality of life effects from migraine: development and psychometric testing of the MSQOL. Headache 1996; 36(8):484-492.	No follow-up data
Migraine	Waldie KE, Poulton R. The burden of illness associated with headache disorders among young adults in a representative cohort study. Headache 2002; 42(7):612-619.	No follow-up data
Migraine	Wang SJ, Fuh JL, Lu SR, Juang KD. Quality of life differs among headache diagnoses: analysis of SF-36 survey in 901 headache patients. Pain 2001; 89(2-3):285-292.	No follow-up data
Migraine	Xuan J, Kirchdoerfer LJ, Boyer JG, Norwood GJ. Effects of comorbidity on health-related quality-of-life scores: an analysis of clinical trial data. Clin Ther 1999; 21(2):383-403.	No follow-up data
Neck pain	Anderson JJ, Ruwe M, Miller DR, Kazis L, Felson DT, Prashker M. Relative costs and effectiveness of specialist and general internist ambulatory care for patients with 2 chronic musculoskeletal conditions. J Rheumatol 2002; 29(7):1488-1495.	No follow-up data
Neck pain	Becker-Witt C, Jena S, Brinkhaus B, Selim D, Liecker B, Willich S. 018 Effectiveness of acupuncture treatment for chronic neck pain: The	No SF-36 data

	acupuncture in routine care (Arc) Study. [Abstract]. J Epidemiol Community Health 2004; 58 Supplement 1:A69.	
Neck pain	Blount KJ, Krompinger WJ, Maljanian R, Browner BD. Moving Toward a Standard for Spinal Fusion Outcomes Assessment. J Spinal Disord Tech 2002; 15(1):16-23.	No SF-36 data
Neck pain	Bono G, Antonaci F, Ghirmai S, D'Angelo F, Berger M, Nappi G. Whiplash injuries: clinical picture and diagnostic work-up. Clin Exp Rheumatol 2000; 18(2 Suppl 19):S23-S28.	Diagnosis not fulfilling criteria
Neck pain	Bronfort G, Evans R, Nelson B, Aker PD, Goldsmith CH, Vernon H. A randomized clinical trial of exercise and spinal manipulation for patients with chronic neck pain. Spine 2001; 26(7):788-797.	Modified SF-36
Neck pain	Carroll L, Mercado AC, Cassidy JD, Cjte P. A population-based study of factors associated with combinations of active and passive coping with neck and low back pain. J Rehabil Med 2002; 34(2):67-72.	No SF-36 data
Neck pain	Daffner SD, Hilibrand AS, Hanscom BS, Brislin BT, Vaccaro AR, Albert TJ. Impact of neck and arm pain on overall health status. Spine 2003; 28(17):2030-2035.	Multiple publication
Neck pain	Di Fabio RP, Boissonnault W. Physical therapy and health-related outcomes for patients with common orthopaedic diagnoses. J Orthop Sports Phys Ther 1998; 27(3):219-230.	No SF-36 data
Neck pain	Fanuele JC, Birkmeyer NJ, Abdu WA, Tosteson TD, Weinstein JN. The impact of spinal problems on the health status of patients: have we underestimated the effect? Spine 2000; 25(12):1509-1514.	No follow-up data
Neck pain	Ferrari R, Russell AS, Carroll LJ, Cassidy JD. A re-examination of the whiplash associated disorders (WAD) as a systemic illness. Ann Rheum Dis 2005; 64(9):1337-1342.	Diagnosis not fulfilling criteria
Neck pain	Gerard S, Smith BH, Simpson JA. A randomized controlled trial of spiritual healing in restricted neck movement. J Altern Complement Med 2003; 9(4):467-477.	Follow-up duration ± > 20%
Neck pain	Giles LG, Muller R. Chronic spinal pain: a randomized clinical trial comparing medication, acupuncture, and spinal manipulation. Spine 2003; 28(14):1490-1502.	Follow-up duration ± > 20%
Neck pain	Hee HT, III WT, III, Myers L, Roesch W, Ricciardi JE. Do worker's compensation patients with neck pain have lower SF-36 scores? Eur Spine J 2002; 11(4):375-381.	No follow-up data
Neck pain	Herman PM, Craig BM, Caspi O. Is complementary and alternative medicine (CAM) cost-effective? A systematic review. BMC Complement Altern Med 2005; 5:11, 2005.	No SF-36 data
Neck pain	Hurwitz E, Morgenstern H, Arber P, ominski G, u F, dams A. A Randomized Trial of Chiropractic Manipulation and Mobilization for Patients With Neck Pain: Clinical Outcomes From the UCLA Neck-Pain Study. Am J Public Health 2002; 92(10):1634-1641.	No SF-36 data
Neck pain	Irnich D, Behrens N, Molzen H, Konig A, Gleditsch J, Krauss M et al. Randomised trial of acupuncture compared with conventional massage and "sham" laser acupuncture for treatment of chronic neck pain. BMJ 2001; 322(7302):1574-1578.	SF-36 data without means
Neck pain	Kohlmann T. [Epidemiology of orofacial pain]. [German]. Schmerz 2002; 16(5):339-345.	Diagnosis not fulfilling criteria
Neck pain	Koller M, Kienapfel H, Hinder D, Sabau E, Wingert G, Pfeiffer M et al. [A scale for measuring symptoms related to degenerative diseases of the cervical spine. A reference in determining indications and evaluating surgical outcome]. [German]. Chirurg 1999; 70(11):1364-1373.	No SF-36 data
Neck pain	Laing RJ, Ng I, Seeley HM, Hutchinson PJ. Prospective study of clinical and radiological outcome after anterior cervical discectomy. Br J Neurosurg 2001; 15(4):319-323.	No SF-36 data

Neck pain	Latimer M, Haden N, Seeley HM, Laing RJ. Measurement of outcome in patients with cervical spondylotic myelopathy treated surgically. British J Neurosurg 2002; 16(6):545-549.	Diagnosis not fulfilling criteria
Neck pain	Ljungquist T, Jensen IB, Nygren A, Harms-Ringdahl K. Physical performance tests for people with long-term spinal pain: Aspects of construct validity. J Rehabil Med 2003; 35(2):69-75.	No SF-36 data
Neck pain	Peolsson M, Gerdle B. Coping in patients with chronic whiplash-associated disorders: A descriptive study. J Rehabil Med 2004; 36(1):28-35.	Diagnosis not fulfilling criteria
Neck pain	Richter M, Ferrari R, Otte D, Kuensebeck HW, Blauth M, Krettek C. Correlation of clinical findings, collision parameters, and psychological factors in the outcome of whiplash associated disorders. J Neurol Neurosurg Psychiatry 2004; 75(5):758-764.	Diagnosis not fulfilling criteria
Neck pain	Riddle DL, Stratford PW. Use of generic versus region-specific functional status measures on patients with cervical spine disorders. Phys Ther 1998; 78(9):951-963.	No follow-up data
Neck pain	Stoll T, Huber E, Bachmann S, Baumeler HR, Mariacher S, Rutz M et al. Validity and Sensitivity to Change of the NASS Questionnaire for Patients With Cervical Spine Disorders. Spine 2004; 29(24):2851-2855.	Diagnosis not fulfilling criteria
Neck pain	Terrell JE, Welsh DE, Bradford CR, Chepeha DB, Esclamado RM, Hogikyan ND et al. Pain, quality of life, and spinal accessory nerve status after neck dissection. Laryngoscope 2000; 110(4):620-626.	Diagnosis not fulfilling criteria
Neck pain	Walker-Bone K, Reading I, Coggon D, Cooper C, Palmer KT. The anatomical pattern and determinants of pain in the neck and upper limbs: an epidemiologic study. Pain 2004; 109(1-2):45-51.	No follow-up data
Neck pain	White P, Lewith G, Prescott P, Conway J. Acupuncture versus Placebo for the Treatment of Chronic Mechanical Neck Pain: A Randomized, Controlled Trial. Ann Intern Med 2004; 141(12):911-919.	No baseline SF-36 data
Neck pain	White PF, Craig WF, Vakharia AS, Ghoname E, Ahmed HE, Hamza MA. Percutaneous neuromodulation therapy: does the location of electrical stimulation effect the acute analgesic response? Anesth Analg 2000; 91(4):949-954.	No follow-up data
Neck pain	Yeung PL, Chiu TT, Leung AS. Use of modified Northwick Park Neck Pain Questionnaire in patients with postirradiation neck disability: Validation study. Head Neck 2004; 26(12):1031-1037.	Diagnosis not fulfilling criteria

*Reasons for exclusion were coded and ranked in the following order (if more than one reason applied, the reason with the highest rank was coded):

1. Diagnosis not fulfilling criteria
2. No SF-36 data
3. Patients > 60 years
4. Cohort with < 20 patients
5. No follow-up data
6. Follow-up duration ± > 20% [from 3, 6, and 12 months, respectively]
7. No trial
8. Multiple publication
9. Language not fulfilling criteria
10. SF-36 acute form only
11. No baseline SF-36 data
12. SF-36 data without means
13. Modified SF-36
14. Other
