# Supplementary material for: Assessing the order of magnitude of outcomes in single-arm cohorts through systematic comparison with corresponding cohorts: An example from the AMOS study
Source: BMC Med Res Methodol. 2008 Mar 19;8:11. doi: 10.1186/1471-2288-8-11 (PMC2323398; doi:10.1186/1471-2288-8-11)
Supplement: Additional file 2 — Included publications. List of included publications [file 1471-2288-8-11-S2.pdf]

Included publications

Asthma

1. Adams RJ, Wilson D, Smith BJ, Ruffin RE: Impact of coping and socioeconomic factors on quality of life in adults with asthma. Respirology 2004, 9: 87-95.
2. Hopman WM, Garvey N, Olajos-Clow J, White-Markham A, Lougheed MD: Outcomes of asthma education: results of a multisite evaluation. Can Respir J 2004, 11: 291-297.
3. Lee TA, Hollingworth W, Sullivan SD: Comparison of directly elicited preferences to preferences derived from the SF-36 in adults with asthma. Med Decis Making 2003, 23: 323-334.
4. Oga T, Nishimura K, Tsukino M, Sato S, Hajiro T, Mishima M: A comparison of the responsiveness of different generic health status measures in patients with asthma. Qual Life Res 2003, 12: 555-563.
5. Okamoto LJ, Noonan M, DeBoisblanc BP, Kellerman DJ: Fluticasone propionate improves quality of life in patients with asthma requiring oral corticosteroids. Ann Allergy Asthma Immunol 1996, 76: 455-461.
6. Olajos-Clow J, Costello E, Lougheed MD: Perceived control and quality of life in asthma: impact of asthma education. J Asthma 2005, 42: 751-756.
7. Rutherford C, Mills R, Gibson PG, Price MJ: Improvement in health-related quality of life with fluticasone propionate compared with budesonide or beclomethasone dipropionate in adults with severe asthma. Respirology 2003, 8: 371-375.
8. Sato S, Nishimura K, Tsukino M, Oga T, Hajiro T, Ikeda A et al.: Possible maximal change in the SF-36 of outpatients with chronic obstructive pulmonary disease and asthma. J Asthma 2004, 41: 355-365.
9. Schmier J, Leidy NK, Gower R: Reduction in oral corticosteroid use with mometasone furoate dry powder inhaler improves health-related quality of life in patients with severe persistent asthma. J Asthma 2003, 40: 383-393.
10. Schulz M, Verheyen F, Muhlig S, Muller JM, Muhlbauer K, Knop-Schneickert E et al.: Pharmaceutical care services for asthma patients: a controlled intervention study. J Clin Pharmacol 2001, 41: 668-676.
11. Vegazo GO, Martin FJ, Barcina SC, Jimenez Jimenez FJ, Estiarte NR: [Impact of the long active beta-2 agonists inhaled therapy on the quality of life in asthmatic patients]. [Spanish]. An Med Interna 2004, 21: 272-278.

Depression

12. Beusterien KM, Buesching DP, Robison RN, Keats MM, Tomlinson JR, Cofran KW et al.: Evaluation of an information exchange program for primary care patients with depression. Dis Manag 2000, 3: 1-9.
13. Ceroni GB, Rucci P, Berardi D, Ceroni FB, Katon W: Case review vs. usual care in primary care patients with depression: a pilot study. Gen Hosp Psychiatry 2002, 24: 71-80.
14. Hedrick SC, Chaney EF, Felker B, Liu CF, Hasenberg N, Heagerty P et al.: Effectiveness of collaborative care depression treatment in Veterans' Affairs primary care. J Gen Intern Med 2003, 18: 9-16.
15. Hirschfeld RM, Dunner DL, Keitner G, Klein DN, Koran LM, Kornstein SG et al.: Does psychosocial functioning improve independent of depressive symptoms? A comparison of nefazodone, psychotherapy, and their combination. Biol Psychiatry 2002, 51: 123-133.
16. King M, Davidson O, Taylor F, Haines A, Sharp D, Turner R: Effectiveness of teaching general practitioners skills in brief cognitive behaviour therapy to treat patients with depression: randomised controlled trial. BMJ 2002, 324: 947-950.

17. Kroenke K, West SL, Swindle R, Gilsenan A, Eckert GJ, Dolor R et al.: Similar effectiveness of paroxetine, fluoxetine, and sertraline in primary care: a randomized trial. JAMA 2001, 286: 2947-2955.

18. Lin EH, von Korff M, Russo J, Katon W, Simon GE, Unutzer J et al.: Can depression treatment in primary care reduce disability? A stepped care approach. Arch Fam Med 2000, 9: 1052-1058.

19. Miller IW, Keitner GI, Schatzberg AF, Klein DN, Thase ME, Rush AJ et al.: The treatment of chronic depression, part 3: psychosocial functioning before and after treatment with sertraline or imipramine. J Clin Psychiatry 1998, 59: 608-619.

20. Nickel C, Lahmann C, Tritt K, Muehlbacher M, Kaplan P, Kettler C et al.: Topiramate in treatment of depressive and anger symptoms in female depressive patients: a randomized, double-blind, placebo-controlled study. J Affect Disord 2005, 87: 243-252.

21. Revicki DA, Simon GE, Chan K, Katon W, Heiligenstein J: Depression, health-related quality of life, and medical cost outcomes of receiving recommended levels of antidepressant treatment. J Fam Pract 1998, 47: 446-452.

22. Simon GE, Revicki DA, Grothaus L, von Korff M: SF-36 summary scores: are physical and mental health truly distinct? Med Care 1998, 36: 567-572.

23. Smith JL, Rost KM, Nutting PA, Elliott CE, Dickinson LM: Impact of ongoing primary care intervention on long term outcomes in uninsured and insured patients with depression. Med Care 2002, 40: 1210-1222.

24. Valenstein M, Ritsema T, Green L, Blow FC, Mitchinson A, McCarthy JF et al.: Targeting quality improvement activities for depression. Implications of using administrative data. J Fam Pract 2000, 49: 721-728.

Low back pain

25. Bronfort G, Bouter LM: Responsiveness of general health status in chronic low back pain: a comparison of the COOP charts and the SF-36. Pain 1999, 83: 201-209.

26. Derby R, Lettice JJ, Kula TA, Lee SH, Seo KS, Kim BJ: Single-level lumbar fusion in chronic discogenic low-back pain: psychological and emotional status as a predictor of outcome measured using the 36-item Short Form. J Neurosurg Spine 2005, 3: 255-261.

27. Freeman BJC, Fraser RD, Cain CMJ, Hall DJ, Chapple DCL: A randomized, double-blind, controlled trial - Intradiscal electrothermal therapy versus placebo for the treatment of chronic discogenic low back pain. Spine 2005, 30: 2369-2377.

28. Frost H, Lamb SE, Doll HA, Carver PT, Stewart-Brown S: Randomised controlled trial of physiotherapy compared with advice for low back pain. BMJ 2004, 329: 708.

29. Garratt AM, Ruta DA, Abdalla MI, Russell IT: SF 36 health survey questionnaire: II. Responsiveness to changes in health status in four common clinical conditions. Qual Health Care 1994, 3: 186-192.

30. Goebel S, Stephan A, Freiwald J: [Strength training in chronic low back pain. Results of a longitudinal study]. [German]. Dtsch Z Sportmed 2005, 56: 388-392.

31. Graz B, Wietlisbach V, Porchet F, Vader JP: Prognosis or "curabo effect?": physician prediction and patient outcome of surgery for low back pain and sciatica. Spine 2005, 30: 1448-1452.

32. Grotle M, Brox JI, Vollestad NK: Concurrent comparison of responsiveness in pain and functional status measurements used for patients with low back pain. Spine 2004, 29: E492-E501.

33. Haid RW, Jr., Branch CL, Jr., Alexander JT, Burkus JK: Posterior lumbar interbody fusion using recombinant human bone morphogenetic protein type 2 with cylindrical interbody cages. - Spine J 2004, 4: 527-538.

34. Hollingworth W, Dixon AK, Todd CJ, Bell MI, Antoun NM, Arafat Q et al.: Self reported health status and magnetic resonance imaging findings in patients with low back pain. Eur Spine J 1998, 7: 369-375.

35. Jarvik JG, Maravilla KR, Haynor DR, Levitz M, Deyo RA: Rapid MR imaging versus plain radiography in patients with low back pain: initial results of a randomized study. Radiology 1997, 204: 447-454.

36. Jarvik JG, Hollingworth W, Martin B, Emerson SS, Gray DT, Overman S et al.: Rapid magnetic resonance imaging vs radiographs for patients with low back pain: a randomized controlled trial.[see comment]. JAMA 2003, 289: 2810-2818.

37. Kagaya H, Takahashi H, Sugawara K, Kuroda T, Takahama M: Quality of life assessment before and after lumbar disc surgery. J Orthop Sci 2005, 10: 486-489.

38. Kosinski MR, Schein JR, Vallow SM, Ascher S, Harte C, Shikiar R et al.: An observational study of health-related quality of life and pain outcomes in chronic low back pain patients treated with fentanyl transdermal system. Curr Med Res Opin 2005, 21: 849-862.

39. Lang E, Liebig K, Kastner S, Neundorfer B, Heuschmann P: Multidisciplinary rehabilitation versus usual care for chronic low back pain in the community: effects on quality of life. Spine J 2003, 3: 270-276.

40. Leggett S, Mooney V, Matheson LN, Nelson B, Dreisinger T, Van Zytveld J et al.: Restorative exercise for clinical low back pain. A prospective two-center study with 1-year follow-up. Spine 1999, 24: 889-898.

41. Patrick DL, Deyo RA, Atlas SJ, Singer DE, Chapin A, Keller RB: Assessing health-related quality of life in patients with sciatica. Spine 1995, 20: 1899-1908.

42. Ruoff GE, Rosenthal N, Jordan D, Karim R, Kamin M, Protocol CAPS: Tramadol/acetaminophen combination tablets for the treatment of chronic lower back pain: a multicenter, randomized, double-blind, placebo-controlled outpatient study. Clin Ther 2003, 25: 1123-1141.

43. Saal JA, Saal JS: Intradiscal electrothermal treatment for chronic discogenic low back pain: prospective outcome study with a minimum 2-year follow-up. Spine 2002, 27: 966-973.

44. Shaughnessy M, Caulfield B: A pilot study to investigate the effect of lumbar stabilisation exercise training on functional ability and quality of life in patients with chronic low back pain. Int J Rehabil Res 2004, 27: 297-301.

45. Suarez-Almazor ME, Kendall C, Johnson JA, Skeith K, Vincent D: Use of health status measures in patients with low back pain in clinical settings. Comparison of specific, generic and preference-based instruments. Rheumatology (Oxford) 2000, 39: 783-790.

46. Thomas KJ, MacPherson H, Ratcliffe J, Thorpe L, Brazier J, Campbell M et al.: Longer term clinical and economic benefits of offering acupuncture care to patients with chronic low back pain. Health Technol Assess 2005, 9: 1-126.

47. Thome C, Barth M, Scharf J, Schmiedek P: Outcome after lumbar sequestrectomy compared with microdiscectomy: a prospective randomized study. J Neurosurg Spine 2005, 2: 271-278.

48. van Wijk RM, Geurts JW, Wynne HJ, Hammink E, Buskens E, Lousberg R et al.: Radiofrequency denervation of lumbar facet joints in the treatment of chronic low back pain: a randomized, double-blind, sham lesion-controlled trial. Clin J Pain 2005, 21: 335-344.

Migraine

49. Bordini CA, Mariano dS, Garbelini RP, Teixeira SO, Speciali JG: Effect of preventive treatment on health-related quality of life in episodic migraine. J Headache Pain 2005, 6: 387-391.

50. Bullinger M: [Assessment of health related quality of life with the SF-36 Health Survey]. [German]. Rehabilitation (Stuttg) 1996, 35: XVII-XXVII.

51. Cohen JA, Beall D, Beck A, Rawlings J, Miller DW, Clements B et al.: Sumatriptan treatment for migraine in a health maintenance organization: economic, humanistic, and clinical outcomes. Clin Ther 1999, 21: 190-204.

52. Dahlof C, Bouchard J, Cortelli P, Heywood J, Jansen JP, Pham S et al.: A multinational investigation of the impact of subcutaneous sumatriptan. II: Health-related quality of life. Pharmacoeconomics 1997, 11 Suppl 1: 24-34.

53. Gerth WC, Ruggles KH, Stark SR, Davies GM, Santanello NC: Improvement in health-related quality of life with rizatriptan 10mg compared with standard migraine therapy. Clin Drug Investig 2001, 21: 853-860.

54. Jhingran P, Cady RK, Rubino J, Miller D, Grice RB, Gutterman DL: Improvements in health-related quality of life with sumatriptan treatment for migraine. J Fam Pract 1996, 42: 36-42.

55. Lainez MJ, Lopez A, Pascual AM: Effects on productivity and quality of life of rizatriptan for acute migraine: a workplace study. Headache 2005, 45: 883-890.

56. Lang E, Kastner S, Neundorfer B, Bickel A: [Effects of recommendations and patient seminars on effectivity of outpatient treatment for headache]. [German]. Schmerz 2001, 15: 229-240.

57. Lofland JH, Johnson NE, Batenhorst AS, Nash DB: Changes in resource use and outcomes for patients with migraine treated with sumatriptan: a managed care perspective. Arch Intern Med 1999, 159: 857-863.

58. Patrick DL, Hurst BC, Hughes J: Further development and testing of the migraine-specific quality of life (MSQOL) measure. Headache 2000, 40: 550-560.

59. Solomon GD, Skobieranda FG, Genzen JR: Quality of life assessment among migraine patients treated with sumatriptan. Headache 1995, 35: 449-454.

60. Soon YY, Siow HC, Tan CY: Assessment of migraineurs referred to a specialist headache clinic in Singapore: diagnosis, treatment strategies, outcomes, knowledge of migraine treatments and satisfaction. Cephalalgia 2005, 25: 1122-1132.

61. Vickers AJ, Rees RW, Zollman CE, McCarney R, Smith CM, Ellis N et al.: Acupuncture for chronic headache in primary care: large, pragmatic, randomised trial. BMJ 2004, 328: 744.

Neck pain

62. Hacker RJ, Cauthen JC, Gilbert TJ, Griffith SL: A prospective randomized multicenter clinical evaluation of an anterior cervical fusion cage. Spine 2000, 25: 2646-2654.

63. Klaber Moffett JA, Jackson DA, Richmond S, Hahn S, Coulton S, Farrin A et al.: Randomised trial of a brief physiotherapy intervention compared with usual physiotherapy for neck pain patients: outcomes and patients' preference. BMJ 2004, 330: 75.
