# Supplementary material for: Assessing the order of magnitude of outcomes in single-arm cohorts through systematic comparison with corresponding cohorts: An example from the AMOS study
Source: BMC Med Res Methodol. 2008 Mar 19;8:11. doi: 10.1186/1471-2288-8-11 (PMC2323398; doi:10.1186/1471-2288-8-11)
Supplement: Additional file 3 — Description of AMOS cohorts and corresponding cohorts, stratified by diagnosis. Descriptive data for AMOS cohorts and for corresponding cohorts on gender, age, study design, setting, disease duration at baseline, study treatment, last follow-up and follow-up rates [file 1471-2288-8-11-S3.pdf]

# Description of AMOS cohorts and corresponding cohorts, stratified by diagnosis

## Table 1 - Percent women

| Diagnosis | AMOS cohorts | | Corresponding cohorts | | |
|---|---|---|---|---|---|
| | N (Women/Total) | Percent | N (Women/Total) | Percent | N (evaluable/ all cohorts) |
| Asthma | 39 / 56 | 70% | 1233 / 2030 | 61% | 12 / 12 |
| Depression | 145 / 174 | 83% | 2796 / 4440 | 63% | 19 / 19 |
| Low back pain | 69 / 80 | 86% | 1777 / 4207 | 42% | 26 / 30 |
| Migraine | 38 / 42 | 90% | 2684 / 3494 | 77% | 18 / 21 |
| Neck pain | 35 / 40 | 88% | 407 / 756 | 54% | 2 / 2 |
| Total | 326 / 392 | 83% | 8897 / 14927 | 60% | 77 / 84 |

## Table 2 - Age

*of patients **of cohorts

| Diagnosis | AMOS cohorts | | Corresponding cohorts | | |
|---|---|---|---|---|---|
| | Mean* | SD* | Mean** | SD** | N (evaluable/ all cohorts) |
| Asthma | 43.78 | 11.35 | 47.18 | 6.72 | 11 / 12 |
| Depression | 43.48 | 11.04 | 44.77 | 5.40 | 17 / 19 |
| Low back pain | 48.34 | 12.03 | 45.97 | 4.85 | 27 / 30 |
| Migraine | 40.00 | 11.71 | 41.21 | 2.77 | 19 / 21 |
| Neck pain | 45.92 | 11.60 | 46.35 | 2.78 | 2 / 2 |
| Total | 44.39 | 11.62 | 44.70 | 5.20 | 76 / 84 |

## Table 3 - Study design of corresponding cohorts

| Diagnosis | Randomised controlled trials | Non-randomised comparative studies | Cohort studies | Total |
|---|---|---|---|---|
| | N (%) | N (%) | N (%) | N (%) |
| Asthma | 5 (42) | 0 (0) | 7 (58) | 12 (100) |
| Depression | 16 (84) | 0 (0) | 3 (16) | 19 (100) |
| Low back pain | 13 (43) | 6 (20) | 11 (37) | 30 (100) |
| Migraine | 4 (19) | 3 (14) | 14 (67) | 21 (100) |
| Neck pain | 2 (100) | 0 (0) | 0 (0) | 2 (100) |
| Total | 40 (48) | 9 (11) | 35 (42) | 84 (100) |

## Table 4 - Setting of corresponding cohorts

| Diagnosis | Primary care / Health Maintenance Organization | Non-academic hospital or clinic | Academic hospital or clinic | Other or not specified | Total |
|---|---|---|---|---|---|
| | N (%) | N (%) | N (%) | N (%) | N (%) |
| Asthma | 1 (8) | 4 (33) | 5 (42) | 2 (17) | 12 (100) |
| Depression | 14 (74) | 4 (21) | 0 (0) | 1 (5) | 19 (100) |
| Low Back Pain | 5 (17) | 9 (30) | 12 (40) | 4 (13) | 30 (100) |
| Migraine | 6 (29) | 2 (10) | 9 (43) | 4 (19) | 21 (100) |
| Neck pain | 1 (50) | 0 (0) | 1 (50) | 0 (0) | 2 (100) |
| Total | 27 (32) | 19 (23) | 27 (32) | 11 (13) | 84 (100) |

**Table 5 - Setting of patients**

*AMOS patients: setting unknown: n = 3

| Diagnosis | AMOS patients* | | | | Corresponding cohort patients | | | | |
|---|---|---|---|---|---|---|---|---|---|
| | Primary care | Referral practice | Out-patient clinic | Total | Primary care / HMO | Non-academic hospital or clinic | Academic hospital or clinic | Other or not specified | Total |
| | N (%) | N (%) | N (%) | N (%) | N (%) | N (%) | N (%) | N (%) | N (%) |
| Asthma | 27 (48) | 29 (52) | 0 (0) | 56 (100) | 304 (15) | 849 (42) | 620 (31) | 257 (13) | 2030 (100) |
| Depression | 158 (91) | 8 (5) | 8 (5) | 174 (100) | 3111 (70) | 0 (0) | 1297 (29) | 32 (1) | 4440 (100) |
| Low back pain | 77 (97) | 1 (1) | 1 (1) | 79 (100) | 857 (18) | 1754 (37) | 1592 (34) | 498 (11) | 4701 (100) |
| Migraine | 36 (88) | 3 (7) | 2 (5) | 41 (100) | 887 (21) | 1139 (27) | 81 (2) | 2133 (50) | 4240 (100) |
| Neck pain | 39 (100) | 0 (0) | 0 (0) | 39 (100) | 268 (35) | 488 (65) | 0 (0) | 0 (0) | 756 (100) |
| Total | 337 (87) | 41 (11) | 11 (3) | 389* (100) | 5427 (34) | 4230 (26) | 3590 (22) | 2920 (18) | 16167 (100) |

**Table 6 - Disease duration in years at baseline**

*of patients. **of cohorts

| Diagnosis | AMOS cohorts | | Corresponding cohorts | | |
|---|---|---|---|---|---|
| | Median* | Interquartile range* | Median** | Interquartile range** | N (evaluable/ all cohorts) |
| Asthma | 14.50 | 6.00-22.25 | 14.50 | 10.35-20.01 | 6 / 12 |
| Depression | 4.00 | 1.50-10.00 | 16.33 | 16.33-16.33 | 1 / 19 |
| Low back pain | 7.00 | 2.00-16.00 | 5.06 | 3.46-10.00 | 3 / 30 |
| Migraine | 12.50 | 6.38-20.00 | 21.33 | 5.25-21.92 | 3 / 21 |
| Neck pain | 4.50 | 0.63-10.00 | 2.32 | 2.32-2.32 | 1 / 2 |
| Total | 6.00 | 2.00-15.75 | 12.08 | 5.20-20.01 | 14 / 84 |

**Table 7 - Study treatment in corresponding cohorts**

N and percentages of cohorts

| Diagnosis | Drugs | Treatment-as-usual | Surgery | Physio-therapy | Other physical therapy | Educa-tional | Mixed or other | Total |
|---|---|---|---|---|---|---|---|---|
| | N (%) | N (%) | N (%) | N (%) | N (%) | N (%) | N (%) | N (%) |
| Asthma | 7 (58) | 2 (17) | 0 (0) | 0 (0) | 0 (0) | 3 (25) | 0 (0) | 12 (100) |
| Depression | 8 (42) | 3 (16) | 0 (0) | 0 (0) | 0 (0) | 2 (11) | 6 (32) | 19 (100) |
| Low Back Pain | 2 (7) | 9 (30) | 7 (23) | 3 (10) | 5 (17) | 1 (3) | 3 (10) | 30 (100) |
| Migraine | 15 (71) | 3 (14) | 0 (0) | 0 (0) | 0 (0) | 1 (5) | 2 (7) | 21 (100) |
| Neck pain | 0 (0) | 0 (0) | 1 (50) | 1 (50) | 0 (0) | 0 (0) | 0 (0) | 2 (100) |
| Total | 32 (38) | 17 (20) | 8 (10) | 4 (5) | 5 (7) | 7 (8) | 11 (13) | 84 (100) |

**Table 8 - Study treatment in corresponding cohort patients**

N and percentages of patients

| Diagnosis | Drugs | Treatment-as-usual | Surgery | Physio-therapy | Other physical therapy | Educa-tional | Mixed or other | Total |
|---|---|---|---|---|---|---|---|---|
| | N (%) | N (%) | N (%) | N (%) | N (%) | N (%) | N (%) | N (%) |
| Asthma | 1062 (52) | 429 (21) | 0 (0) | 0 (0) | 0 (0) | 539 (27) | 0 (0) | 2030 (100) |
| Depression | 2569 (58) | 352 (8) | 0 (0) | 0 (0) | 0 (0) | 323 (7) | 1196 (27) | 4440 (100) |
| Low back pain | 412 (8) | 1302 (28) | 961 (20) | 1360 (29) | 232 (5) | 50 (1) | 384 (8) | 4701 (100) |
| Migraine | 3612 (85) | 326 (8) | 0 (0) | 0 (0) | 0 (0) | 46 (1) | 256 (6) | 4240 (100) |
| Neck pain | 0 (0) | 0 (0) | 488 (65) | 268 (35) | 0 (0) | 0 (0) | 0 (0) | 756 (100) |
| Total | 7655 (47) | 2409 (15) | 1449 (9) | 1628 (10) | 232 (1) | 958 (6) | 1836 (11) | 16167 (100) |

**Table 9 - Last follow-up of corresponding cohorts**

N and percentages of cohorts. Last follow-up was after 12 months for all A-patients.

| Diagnosis | 3 Months | 6 Months | 12 Months | Total |
|---|---|---|---|---|
| | N (%) | N (%) | N (%) | N (%) |
| Asthma | 3 (25) | 4 (33) | 5 (42) | 12 (100) |
| Depression | 9 (47) | 8 (42) | 2 (11) | 19 (100) |
| Low Back Pain | 8 (27) | 8 (27) | 14 (47) | 30 (100) |
| Migraine | 3 (14) | 12 (57) | 6 (29) | 21 (100) |
| Neck pain | 0 (0) | 0 (0) | 2 (100) | 2 (100) |
| All | 23 (27) | 32 (38) | 29 (35) | 84 (100) |

**Table 10 - Follow-up rates**

*****of patients. **of cohorts. Number of evaluable corresponding cohorts at each follow-up: See Table 9

| Diagnosis | 3 Months | | 6 Months | | 12 Months | |
|---|---|---|---|---|---|---|
| | AMOS cohorts* | Corresponding cohorts** | AMOS cohorts* | Corresponding cohorts** | AMOS cohorts* | Corresponding cohorts** |
| Asthma | 80.4% | 58.0% | 67.9% | 70.7% | 73.2% | 73.8% |
| Depression | 87.9% | 86.4% | 85.1% | 82.1% | 78.7% | 87.0% |
| Low Back Pain | 86.3% | 82.1% | 83.8% | 82.8% | 80.0% | 70.0% |
| Migraine | 90.5% | 88.1% | 78.6% | 79.6% | 73.8% | 76.1% |
| Neck pain | 95.0% | Not applicable | 90.0% | Not applicable | 90.0% | 74.7% |
| All | 87.5% | 83.0% | 82.1% | 79.1% | 78.8% | 72.2% |
