# Supplementary material for: Assessing the order of magnitude of outcomes in single-arm cohorts through systematic comparison with corresponding cohorts: An example from the AMOS study
Source: BMC Med Res Methodol. 2008 Mar 19;8:11. doi: 10.1186/1471-2288-8-11 (PMC2323398; doi:10.1186/1471-2288-8-11)
Supplement: Additional file 4 — Comparative analyses, stratified by diagnosis and SF-36 scales. Baseline scores of AMOS cohorts and of corresponding cohorts as well as between-group outcome differences, stratified by diagnosis and SF-36 scales [file 1471-2288-8-11-S4.pdf]

Comparative analyses, stratified by diagnosis and SF-36 scales

Table 1 - Asthma: Baseline scores and outcome comparisons of SF-36 scales

*****of patients. **of cohorts. ***of differences. N: Number of cohorts. 25-per: 25-percentile. 75-per: 75-percentile. Each outcome difference refers to one SF-36 scale at the last evaluable follow-up of the corresponding cohort: Mean difference from baseline in AMOS cohort minus mean difference from baseline in corresponding cohort. A positive difference indicates that AMOS cohorts show larger improvements than corresponding cohorts.

SF-36 scale	Baseline							Follow-up				
	AMOS cohort			Corresponding cohorts				Outcome differences				
	Mean *	SD *	Median *	N **	Mean **	SD **	Median **	Mean ***	SD ***	25-per ***	Median ***	75-per ***
Physical Function	73.0	24.8	78.9	9	66.0	8.7	66.4	0.3	7.3	-5.7	0.2	5.7
Role Physical	53.3	41.6	50.0	9	50.9	10.0	55.3	13.7	21.4	-3.4	6.4	28.5
Role Emotional	72.6	35.5	100.0	9	65.7	10.3	63.3	-10.0	11.8	-17.4	-9.6	0.5
Social Functioning	69.4	26.4	75.0	9	71.5	6.1	72.1	4.9	7.0	-0.9	5.9	9.8
Mental Health	63.4	20.2	68.0	9	68.0	6.3	68.3	0.8	4.3	-3.6	2.2	4.4
Bodily Pain	72.2	29.8	84.0	9	67.6	6.6	67.6	1.5	9.7	-5.8	1.6	9.4
Vitality	45.1	17.2	40.0	9	50.6	3.7	50.8	2.2	7.4	-2.9	1.6	8.1
General Health	52.5	20.0	55.0	9	49.9	6.4	51.5	-2.1	6.0	-8.2	-2.4	4.0
Physical Component	43.7	10.7	46.2	5	40.9	5.5	39.7	2.1	5.5	-2.5	3.9	5.8
Mental Component	44.9	10.8	46.3	4	45.3	3.0	44.4	0.8	3.4	-2.7	1.5	3.7

Table 2 - Depression: Baseline scores and outcome comparisons of SF-36 scales

*****of patients. **of cohorts. ***of differences. N: Number of cohorts. 25-per: 25-percentile. 75-per: 75-percentile. Each outcome difference refers to one SF-36 scale at the last evaluable follow-up of the corresponding cohort: Mean difference from baseline in AMOS cohort minus mean difference from baseline in corresponding cohort. A positive difference indicates that AMOS cohorts show larger improvements than corresponding cohorts.

SF-36 scale	Baseline							Follow-up				
	AMOS cohort			Corresponding cohorts				Outcome differences				
	Mean *	SD *	Median *	N **	Mean **	SD **	Median **	Mean ***	SD ***	25-per ***	Median ***	75-per ***
Physical Function	75.4	22.6	80.0	8	72.7	17.3	74.8	4.8	9.8	-0.1	1.9	6.2
Role Physical	34.0	37.2	25.0	7	53.0	8.6	50.0	13.2	6.8	8.8	11.3	16.0
Role Emotional	24.0	32.0	0.0	8	31.0	15.3	27.0	3.8	10.8	-4.2	4.5	10.5
Social Functioning	44.5	22.5	43.8	14	48.8	7.8	50.1	1.3	8.8	-3.7	-0.4	8.2
Mental Health	38.1	16.5	36.0	9	42.2	3.2	42.9	-0.4	10.1	-10.3	3.4	9.7
Bodily Pain	52.4	28.6	41.0	8	58.9	9.9	58.6	6.6	6.4	2.0	3.7	10.3
Vitality	26.4	15.5	25.0	8	29.9	6.7	29.9	0.3	10.7	-8.3	0.0	10.9
General Health	44.1	19.7	45.0	10	57.6	10.4	60.1	2.8	3.3	-0.1	2.2	5.1
Physical Component	44.0	10.4	42.5	5	42.5	6.6	47.0	4.4	1.7	3.2	3.5	6.0
Mental Component	27.8	9.5	26.2	8	29.3	8.7	28.3	-1.0	7.6	-7.6	-3.5	6.1

Table 3 - Low back pain: Baseline scores and outcome comparisons of SF-36 scales

*of patients. **of cohorts. ***of differences. N: Number of cohorts. 25-per: 25-percentile. 75-per: 75-percentile. Each outcome difference refers to one SF-36 scale at the last evaluable follow-up of the corresponding cohort: Mean difference from baseline in AMOS cohort minus mean difference from baseline in corresponding cohort. A positive difference indicates that AMOS cohorts show larger improvements than corresponding cohorts.

SF-36 scale	Baseline							Follow-up				
	AMOS cohort			Corresponding cohorts				Outcome differences				
	Mean *	SD *	Median *	N **	Mean **	SD **	Median **	Mean ***	SD ***	25-per ***	Median ***	75-per ***
Physical Function	60.9	22.9	65.0	26	47.6	12.6	44.8	-1.8	12.4	-7.8	0.6	7.6
Role Physical	28.8	34.9	25.0	24	22.5	12.3	20.1	2.8	11.3	-6.1	5.4	12.0
Role Emotional	49.4	42.4	33.3	23	56.3	12.1	57.0	0.0	10.2	-4.3	-2.0	8.9
Social Functioning	60.3	24.5	62.5	24	57.4	12.6	56.9	-0.7	8.8	-8.5	1.9	5.4
Mental Health	55.5	18.7	56.0	25	62.5	6.5	63.4	2.1	7.2	-0.5	2.3	5.5
Bodily Pain	32.1	18.5	32.0	28	32.1	9.1	31.1	-0.6	12.0	-9.9	1.7	6.1
Vitality	36.5	15.7	35.0	24	43.2	5.3	42.6	3.7	6.0	-0.8	4.5	8.7
General Health	50.7	19.1	50.0	25	60.0	8.8	59.9	5.3	4.0	2.3	5.4	8.9
Physical Component	35.5	8.3	35.1	9	29.1	3.6	28.5	1.0	4.5	-2.2	2.7	5.1
Mental Component	41.3	11.1	42.4	10	43.7	6.9	43.4	0.0	2.8	-2.2	-0.5	1.8

Table 4 - Migraine: Baseline scores and outcome comparisons of SF-36 scales

*of patients. **of cohorts. ***of differences. N: Number of cohorts. 25-per: 25-percentile. 75-per: 75-percentile. Each outcome difference refers to one SF-36 scale at the last evaluable follow-up of the corresponding cohort: Mean difference from baseline in AMOS cohort minus mean difference from baseline in corresponding cohort. A positive difference indicates that AMOS cohorts show larger improvements than corresponding cohorts.

SF-36 scale	Baseline							Follow-up				
	AMOS cohort			Corresponding cohorts				Outcome differences				
	Mean *	SD *	Median *	N **	Mean **	SD **	Median **	Mean ***	SD ***	25-per ***	Median ***	75-per ***
Physical Function	82.3	22.3	95.0	18	85.9	4.0	85.2	2.4	1.9	1.1	2.6	3.9
Role Physical	42.9	39.2	50.0	18	51.3	13.3	55.8	-3.0	11.3	-12.3	-4.3	5.6
Role Emotional	58.7	44.1	66.7	18	71.2	8.8	72.1	1.8	10.5	-7.4	-0.5	9.4
Social Functioning	55.4	20.5	56.3	18	69.4	6.5	69.3	6.6	9.8	-0.1	6.1	17.2
Mental Health	56.4	19.5	58.0	18	67.3	7.5	67.2	3.1	4.8	-0.4	0.5	7.0
Bodily Pain	41.5	19.9	41.0	18	50.6	8.9	51.0	6.6	6.4	0.1	6.9	11.6
Vitality	41.7	18.4	40.0	18	53.2	5.8	51.6	4.6	6.0	0.1	3.6	9.7
General Health	55.6	19.7	55.0	18	67.2	7.4	67.3	1.6	6.0	-3.9	2.2	6.8
Physical Component	42.5	8.9	43.3	7	42.0	4.4	41.0	0.6	1.6	0.1	0.8	1.5
Mental Component	39.8	12.1	43.2	7	45.4	4.4	46.4	2.3	5.2	-2.8	3.1	5.6

Table 5 - Neck pain: Baseline scores and outcome comparisons of SF-36 scales

*of patients. **of cohorts. ***of differences. N: Number of cohorts. 25-per: 25-percentile. 75-per: 75-percentile. Each outcome difference refers to one SF-36 scale at the last evaluable follow-up of the corresponding cohort: Mean difference from baseline in AMOS cohort minus mean difference from baseline in corresponding cohort. A positive difference indicates that AMOS cohorts show larger improvements than corresponding cohorts.

SF-36 scale	Baseline							Follow-up				
	AMOS cohort			Corresponding cohorts				Outcome differences				
	Mean *	SD *	Median *	N **	Mean **	SD **	Median **	Mean ***	SD ***	25-per ***	Median ***	75-per ***
Physical Function	74.8	23.4	85.0	1	76.2	-	76.2	0.2	-	0.2	0.2	0.2
Role Physical	41.9	39.4	25.0	1	67.7	-	67.7	11.8	-	11.8	11.8	11.8
Role Emotional	48.3	44.6	50.0	1	78.2	-	78.2	17.2	-	17.2	17.2	17.2
Social Functioning	68.1	20.0	68.8	1	72.9	-	72.9	9.4	-	9.4	9.4	9.4
Mental Health	58.2	20.2	56.0	1	70.0	-	70.0	9.5	-	9.5	9.5	9.5
Bodily Pain	41.9	19.7	41.0	1	44.6	-	44.6	7.8	-	7.8	7.8	7.8
Vitality	40.8	18.3	40.0	1	49.7	-	49.7	9.7	-	9.7	9.7	9.7
General Health	48.9	19.1	48.5	1	66.6	-	66.6	10.7	-	10.7	10.7	10.7
Physical Component	40.4	8.7	40.9	1	34.5	-	34.5	-4.2	-	-4.2	-4.2	-4.2
Mental Component	41.0	12.4	40.7	1	42.9	-	42.9	-3.1	-	-3.1	-3.1	-3.1
